# Measurement of the Universal Gas Constant R Using a Spherical Acoustic Resonator

**DOI:** 10.6028/jres.093.010

**Published:** 1988-04-01

**Authors:** M. R. Moldover, J. P. M. Trusler, T. J. Edwards, J. B. Mehl, R. S. Davis

**Affiliations:** National Bureau of Standards, Gaithersburg, MD 20899; University of Delaware, Newark, DE 19716; National Bureau of Standards, Gaithersburg, MD 20899

**Keywords:** argon, fundamental constants, ideal gas, mercury, molar gas constant, *R*, resonator, speed of sound, spherical resonator, temperature, thermometry, Universal Gas Constant

## Abstract

We report a new determination of the Universal Gas Constant *R:* (8.314 471 ±0.000 014) J·mol^−1^K^−1^. The uncertainty in the new value is 1.7 ppm (standard error), a factor of 5 smaller than the uncertainty in the best previous value. The gas constant was determined from measurements of the speed of sound in argon as a function of pressure at the temperature of the triple point of water. The speed of sound was measured with a spherical resonator whose volume was determined by weighing the mercury required to fill it at the temperature of the triple point. The molar mass of the argon was determined by comparing the speed of sound in it to the speed of sound in a standard sample of argon of accurately known chemical and isotoptic composition.

## 1. Introduction

With readily available technology (such as triple point cells and platinum resistance thermometers), it is possible to define and reproduce states of particular temperatures with much greater precision than it is possible to measure the fundamental statistical mechanical quantities characterizing such states (such as the average energy in each degree of freedom of a many body system or the derivative of the internal energy with respect to the entropy at constant volume). Accordingly, the International System of Units (SI) has defined temperature as an independent physical quantity and the kelvin (K) as the unit of that quantity. The definition specifies that the temperature *T*_t_ of the triple point of water is exactly 273.16 K. Having made this specification, one can define the Boltzmann constant *k*, as the ratio 2*E/T*_t_ and one can define the universal gas constant *R*, as the ratio 2*EN*_A_/*T*_t_. (Here *E* is the average kinetic energy in a single mechanical degree of freedom at *T*_t_ and *N*_A_ is the Avogadro constant.) In this work we have re-determined *R* with the result:

R=(8.314471±0.000014)J/(mol⋅K)(1.7ppm), (1.1)

where the error quoted is a standard deviation. From this new value of *R* one can obtain improved values of the Boltzmann constant:

$$k=R/N_{A}=(1.380\;651\;3\pm 0.000\;002\;5)\times 10^{-23}J/K\;(1.8\;\text{ppm}),$$ (1.2)

and the Stefan-Boltzmann constant *σ*:

$$\begin{array}{l} \sigma =2\pi^{5}k^{4}/(15h^{3}c^{2})=2\pi^{5}R^{4}/[15N_{A}{\left(N_{A}h\right)}^{3}c^{2}]\\ =(5.670\;399\pm 0.000\;038)\times 10^{-8}W/(m^{2}\cdot K^{4})\;(6.8\;\text{ppm}). \end{array}$$ (1.3)

Here, we have used the values of *N*_A_, the Planck constant *h*, and the more accurately known molar Planck constant *N*_A_*h* resulting from the 1986 adjustment of the fundamental physical constants [1].

**Figure 1:**
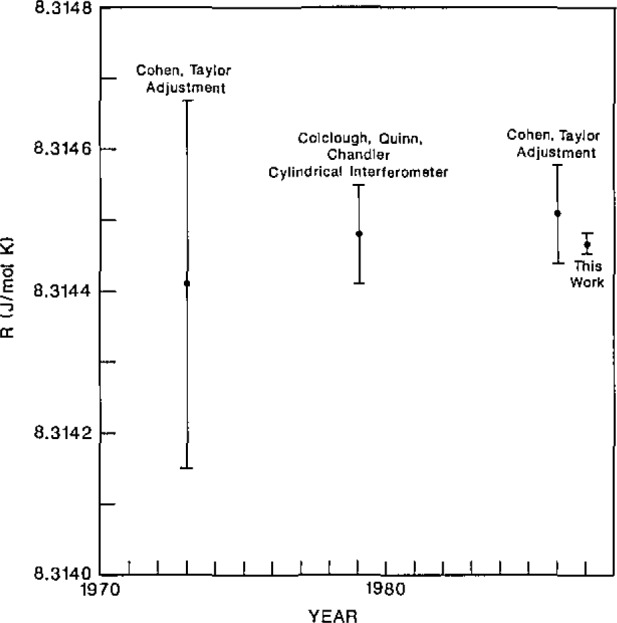
Recent values of *R*. The values from Cohen and Taylor [1] are adjustments. The value from Colclough et al. [13] was obtained with a cylindrical acoustic resonator.

The present value of *R* is compared with other recent determinations of *R* in figure 1. Our value is consistent with previous values; however, it is 5 times more accurate than the best previous value. [The same is true for the values of *k* and *σ* given in eqs (1.4) and (1.5).] This higher accuracy will be most useful for primary thermometry (e.g., gas, noise, acoustic, and radiation thermometry) with thermometers whose design does not permit them to be used at *T*_t_ where the temperature scale is defined. Further details concerning the roles of *R* in metrology and the methods used in prior determinations of *R* have been reviewed by Colclough [2] in 1984 and will not be repeated here.

We conclude this introduction with a summary of the errors in our redetermination of *R*, the prospects for further improvements, and a brief list of the technical advances developed in this work. For these purposes it is useful to present a highly simplified equation relating *R* to the quantities actually measured in the laboratory.

Our redetermination of *R* is based on a new measurement of the speed of sound in a well-characterized sample of argon at *T*_t_. Elementary considerations of hydrodynamics and the kinetic theory of dilute gases lead to the relations:

$$\frac{1}{2}mv_{\text{rms}}^{2}=\frac{3}{2}kT,\;c^{2}=\frac{\gamma }{3}v_{\text{rms}}^{2}.$$ (1.4)

(Here $v_{\text{rms}}^{2}$ is the mean square speed of the molecules, *m* is the mass of one molecule, *c* is the speed of sound, and *γ* is the ratio of the specific heat capacities *C_p_/C_v_* and has the value *γ*_0_=5/3 for dilute monatomic gases.) Thus, a measurement of the speed of sound in a dilute monatomic gas at *T*_t_ is a measurement of the *v*_rms_ at *T*_t_ and it would be a measurement of *k* if *m* were known accurately. In practice the relative values of isotopic masses are extremely well known on a scale of atomic mass units but the knowledge of *m* for any *pure* gas is limited by the uncertainty in *N*_A_, the constant which relates the atomic mass unit to the kilogram and also relates the mass *m* of a molecule to the molar mass *M* [3].

In this work the speed of sound was deduced from measurements of the internal volume of a spherical shell and the frequencies *f*_0_*_n_* of the radially-symmetric acoustic resonances when the shell was filled with argon. There is a well-developed theory for such resonances [4–10] which has been confirmed by detailed experiments [9–11]. The frequencies of the radially-symmetric modes are insensitive to geometric imperfections that leave the internal volume of the shell unchanged [4–6]. Thus, accurate internal dimensional measurements were not required. The internal volume *V* was determined by weighing the quantity of mercury required to fill the shell completely at *T*_t_. *R* is related to the frequencies, volume, and the molar mass by the equations:

$$R=\frac{c_{0}^{2}M}{T_{t}\gamma_{0}}=\frac{1}{T_{t}}{\left(f_{0n}/v_{0n}\right)}^{2}V^{2/3}\frac{M}{\gamma_{0}}.$$ (1.5)

(*v*_0_*_n_* is the eigenvalue *z*_0_*_n_* multiplied by the factor (6*π*^2^)^−1/3^ and is known exactly and $c_{0}^{2}$ is the zero- pressure limit of the speed of sound.)

Table 1 lists the important contributions to the standard error of *R* from the measurements of the quantities in eq (1.5). We now consider them in turn.

The uncertainty in the volume determination is dominated by our imperfect knowledge of the thermal expansion of mercury between *T*_t_ and 20 °C. The random error of the volume determinations is only 0.29 ppm and it contributes 0.20 ppm to the standard error in *R*. It is unusual for an assembled artifact of this size (3 liters) to have such a reproducible internal volume.

In the present work, capsule platinum resistance thermometers were calibrated at *T*_t_ and then inserted in the enclosed acoustic apparatus. The dominant uncertainty in the thermometry resulted from drifts in the thermometers and/or the bridge used with them during the weeks between calibrations. This uncertainty could be greatly reduced by designing an acoustic apparatus which permitted rapid insertion and removal of calibrated thermometers.

In the present work, *M/γ*_0_ was deduced from measurements of the concentrations of the isotopic argon species and noble gas impurities in a standard sample of nearly monoisotopic ^40^Ar. Routine gas chromatographic techniques were used to measure the concentrations of the noble gas impurities in the standard sample. The detection limit for xenon led to the 0.7 ppm uncertainty in *M/γ*_0_. This source of uncertainty could certainly be reduced in future work. The spherical acoustic resonator was used to compare the speed of sound in the standard sample with that in working samples of argon. The comparison had an imprecision of only 0.2 ppm; thus, the ratio of the average molar masses of the argon samples could be determined with an imprecision of 0.4 ppm.

The errors in the measurements of the resonance frequencies are quite small; however, the zero- pressure limit of (*f*_0_*_n_/v*_0_*_n_*)^2^ was determined by fitting a 4-parameter function of pressure to the measured frequencies in the pressure range 25–500 kPa. The correlations among the 4 parameters contributed an uncertainty of 0.68 ppm to $c_{0}^{2}$ [which is the zero-pressure limit of (*f*_0_*_n_*/*v*_0_*_n_*)^2^ multiplied by *V*^2/3^]. This uncertainty could be reduced by increasing the signal-to-noise ratio of the acoustic measurements, particularly at the lower pressures.

The resonance frequencies are perturbed by the presence of a thermal boundary layer (roughly 50 μm thick) in the gas in contact with the shell. This perturbation is proportional to the square root of the thermal diffusivity. Thus, it varies as (*f*_0_*_n_p*)^−1/2^ at low pressures and it ranges from 40–360 ppm. The perturbation has been calculated from independent information about the thermal conductivity of argon. We estimate the uncertainty in the thermal conductivity to be 0.3% and this propagates into a 0.30 ppm uncertainty in the zero-pressure limit of (*f*_0_*_n_*/*v*_0_*_n_*)^2^ and in *R*. The same boundary layer makes the dominant contribution to the widths of the resonances. At low pressures the measured and calculated widths of the resonances are in agreement, which confirms the perturbation calculation.

The final contribution to the uncertainty in the zero-pressure limit of (*f*_0_*_n_*/*v*_0_*_n_*)^2^ resulted from a possible problem in defining the location of one transducer during some of the frequency measurements and could have been eliminated if the opportunity to repeat these measurements were available.

In summary, straightforward modifications of the present measurements might reduce the uncertainty in *R* somewhat, but probably by less than a factor of 2. Further improvement would require two developments: 1. new transducers with improved signal-to-noise characteristics (without degrading the other characteristics required by the measurement), and 2. either a better value for the density of mercury at *T*_t_ or a better method of measuring the resonator’s volume. Microwave measurements are a promising alternative to weighing mercury for volume measurements. A theorem derived by two of us (JBM and MRM) suggests a strategy for doing this with high accuracy using comparatively few microwave resonances in a spherical cavity constructed with ordinary machine shop tolerances [12].

Prior to the present measurements, the most accurate determination of *R* was that of Colclough, Quinn, and Chandler [13]. Their work was also based on measurements of the speed of sound in dilute argon. We shall briefly contrast the two acoustic measurements.

Colclough et al. used a variable-pathlength, cylindrical interferometer operating at 5.6 kHz. They used optical techniques to measure the displacement required of one end of the interferometer to achieve successive longitudinal resonances. In contrast, we have used a spherical resonator of fixed dimensions which was operated near five different radially-symmetric modes at frequencies in the range 2.5–9.5 kHz. Our gravimetric volume determination takes the place of their displacement measurement.

The corrections to the resonance frequencies arising from boundary layers were a factor of 10 smaller for the radial modes in the 18-cm diameter sphere than for the longitudinal modes in the 3-cm diameter cylinder. Because the radial corrections were smaller they could be calculated with sufficient accuracy from independent measurements of the transport properties. The calculations were confirmed by acoustic measurements of the half-widths of the resonances.

The resonances in the sphere were a factor of 10 narrower than in the cylinder. This enabled us to use smaller (6-mm diameter) transducers which perturbed the radially symmetric resonances in a minor and easily calculable fashion while attaining a satisfactory signal-to-noise ratio. In contrast, a larger electroacoustic transducer formed one end of the cylindrical interferometer. The large transducer exhibited nonlinear behavior which caused problems in interpretation of the acoustic data.

In the present work, we have accounted for the effects of the finite elastic compliance of the resonator’s wall and for the incomplete thermal accommodation of the gas at the wall, two phenomena which were not considered by Colclough et al. (The compliance produces a perturbation which is linear in the pressure; thus it does not affect *R*.)

The primary divisions of the remainder of this manuscript are: 2. Theoretical Basis of the Measurement, 3. Fabrication and Characterization of the Resonator, 4. Measuring Resonance Frequencies, 5. Thermometry, 6. Determination of the Resonator’s Volume, 7. Determination of *M/γ*_0_, 8. The Pressure and Other Thermodynamic and Transport Properties, 9. Determination of $c_{0}^{2}$ in the Working Gas, 10. Other Tests for Systematic Errors, and 11. Summary.

## 2. Theoretical Basis of Measurement

### 2.1 Introduction

In this section we describe the acoustical model of the spherical resonator. The model includes a calculation of the response of the gas and the shell to excitation by a steady sinusoidal source, and also includes calculation of the fundamental resonance parameters which appear in the response function. It is convenient to assume that all of the “small” quantities of linear acoustics are proportional to *e^iωt^*, and to obtain solutions in the form of linear combinations of appropriate eigenfunctions. In the following development, we assume the eventual inclusion of a source term whose strength and frequency remain constant long enough for the system to reach a steady state.

We begin with a zero-order description of the acoustics of the gas-filled resonator. This description is not complete enough for calculations of the required accuracy, but it is the simplest way to introduce the basic concept and language used in the complete model. Let the acoustic field in the gas be described by a velocity potential Ψ(*r*), which is related to the particle velocity *u* through

u(r)=∇Ψ(r). (2.1)

If the dynamics of the gas are governed by the Euler equation, and an adiabatic equation of state is used for the gas, then the acoustic pressure *p*′ is related to the velocity potential through

$$p^{\prime }(r)=-i\omega \rho \Psi (r),$$ (2.2)

where *ρ* is the mass density of the gas. The velocity potential satisfies the steady-state wave equation

$$(\nabla^{2}+k^{2})\Psi (r)=0,$$ (2.3)

where *c* is the speed of sound, and *k = ω/c*. The regular solutions of eq (2.3) in spherical coordinates have the form

$$\Psi (r)\propto j_{l}(kr)Y_{lm}(\theta ,\phi ),$$ (2.4)

where *j_l_*(*z*) is a spherical Bessel function and *Y_lm_* is a spherical harmonic. In a spherical shell which is perfectly rigid and insulating, free vibrations of the gas are permitted at frequencies such that the radial particle velocity vanishes at the inner shell wall (*r=a*). These frequencies, which will be referred to as the unperturbed eigenfrequencies, are given by

$$f_{ln}^{0}=cz_{ln}/(2\pi a).$$ (2.5)

where *z_ln_=k_ln_a* is the *n* th root of the equation $\frac{dj_{l}(z)}{dz}=0$. Note that the unperturbed eigenfrequencies are independent of the mode index *m*; for each pair of indices *ln* there are 2*l* + 1 modes with the same frequency $f_{ln}^{0}$. The modes with *l* = 0 are non- degenerate; they will be referred to as the radial modes. They have numerous special properties which make them most suitable for use in high-accuracy acoustical measurements. According to the conventional numbering of roots of Bessel functions, the first *l* = 0 root is designated z_01_ = 0. The (0,2) through (0,6) radial modes were used in the present work. For argon at 273.16 K in the resonator used in this work, the range of frequencies is between $f_{02}^{0}=2476\;\text{Hz}$ and $f_{06}^{0}=9490\;\text{Hz}$. In specifying the numerical values of quantities for argon, it will be convenient to use the dimensionless quantities $\tilde{f}\equiv f/f_{02}^{0}$ and $\tilde{p}\equiv p/(100\;\text{kPa})$. The present measurements span the range $1<\tilde{f}<4.5$ and $0.25<\tilde{p}<5.0$.

The unperturbed eigenfrequencies are proportional to the ratio of the speed of sound to the radius of the resonator. Measurement of an unperturbed eigenfrequency and the radius of the sphere gives a value for the speed of sound. This is the basic principle of our measurement. We made high-precision measurements of the experimental eigenfrequencies of the system. These must be related to the speed of sound and the mean resonator radius by a more complete model of the acoustical system. A description of this model is given in the remainder of this section. The model predicts complex eigenfrequencies *F_N_=f_N_ + ig_N_*, where *N* is shorthand for the notation using multiple indices. The complex eigenfrequency differs from the corresponding unperturbed value $f_{ln}^{0}$ in both the real and imaginary parts. The imaginary part represents the losses; it can be observed experimentally either as the halfwidth of a resonance curve or the decay constant of free oscillations.

The most complete derivation of the relevant theory is presented in Moldover, Mehl, and Greenspan [9]. Their model is incomplete in one respect, however. They use a boundary condition for the temperature at the shell boundary which is not sufficiently accurate at the lowest experimental pressures. This effect was analyzed by Ewing, McGlashan, and Trusler [10]. It is incorporated in the description of the theoretical model presented here.

### 2.2 Basic Equations

The basis of the theoretical model is a set of equations first derived by Kirchhoff in 1868 [14,15]. For completeness, we indicate the nature of the fundamental assumption and the use of constitutive relations in the following. The dynamics of the gas are described by the Navier-Stokes equation, which consists of Newton’s second law and a constitutive relation giving the stresses in terms of the spatial derivatives of the velocity of the gas. The relevant kinetic coefficient is the shear viscosity η. Heat flow in the gas is assumed to be governed by Fourier’s law; the relevant kinetic coefficient is the coefficient of thermal conductivity λ. Two additional equations are statements of the equation of continuity for mass flow and for heat flow. An equation of state for the gas is used to relate changes in pressure, density, and temperature. The second law of thermodynamics is also used to relate changes in the entropy of the gas to temperature and pressure variations. These principles are used to construct a linear acoustic theory. That is, the squares and products of certain “small” quantities are neglected in the equations. Let the pressure, temperature, and density be represented by *p+p′*(*r*), *T* + *τ*(*r*), and *ρ*+*ρ*′(*r*), where *p, T*, and *ρ* are the ambient quantities, and *p*′(*r*), *τ*(*r*) and *ρ*′(*r*) are small terms with the assumed time dependence. The equations of motion couple these fields with each other and with the longitudinal particle velocity *u*(*r*). Kirchhoff found that *τ*(*r*) was governed by a fourth order partial differential equation which may be written in the form

$$(i\delta_{t}^{2}/2)\left[1+\left(i\gamma /2\right){\left(\omega \delta_{v}^{\prime }/c\right)}^{2}\right]\nabla^{4}\tau +\left[1+\left(i/2\right){\left(\omega /c\right)}^{2}\left(\gamma \delta_{t}^{2}+\delta_{v}^{\prime 2}\right)\right]\nabla^{2}\tau +{\left(\omega /c\right)}^{2}\tau =0.$$ (2.6)

The characteristic lengths in this equation are the thermal penetration length

$$\delta_{t}=\sqrt{2D_{t}/\omega },$$ (2.7)

the viscous penetration length

$$\delta_{v}=\sqrt{2D_{v}/\omega },$$ (2.8)

and a supplementary quantity $\delta_{v}^{\prime }$ which is related to the viscous penetration length and the bulk viscosity *η*_b_ by

$$\delta_{v}^{\prime 2}=\frac{4}{3}\delta_{v}^{2}+\eta_{b}/\rho .$$ (2.9)

In these equations the thermal diffusivity *D*_t_ is equal to the ratio of the thermal conductivity to the constant-pressure specific heat capacity per unit volume λ/(*ρC_ρ_*), and the viscous diffusivity *D*_v_=η/*ρ* is equal to the ratio of the viscosity to the density. For argon at 273.16 K. approximate values for these lengths are $\delta_{t}=47.6\;\mu m{\left(\tilde{f}\tilde{p}\right)}^{-1/2}$, $\delta_{v}=38.8\;\mu m{\left(\tilde{f}\tilde{p}\right)}^{-1/2}$, and $\delta_{v}^{\prime }=44.8\;\mu m{\left(\tilde{f}\tilde{p}\right)}^{-1/2}$. (For argon and other monatomic gases, the bulk viscosity term is negligible; it is included here for completeness.)

The pressure is related to the temperature through

$$p^{\prime }(r)=\frac{\gamma \alpha }{\gamma -1}\left[1-\frac{\delta_{t}^{2}}{2i}\nabla^{2}\right]\tau (r),$$ (2.10)

where *γ=C_p_/C_v_* is the ratio of the specific heat capacities and *α* = (∂*p*/∂*T)_v_*. The longitudinal particle velocity *u*(*r*) is related to the pressure and temperature by

$$i\omega \rho u=-\nabla \left[p^{\prime }+\left(i\gamma /2\right){\left(\omega \delta_{v}^{\prime }/c\right)}^{2}\left(p^{\prime }-\alpha \tau \right)\right].$$ (2.11)

A divergence-free component of the velocity is also needed in the complete theory. This component is necessary to describe the shear waves which couple to nonradial modes through the boundary conditions. It is not needed in the present discussion, which will be restricted to radial modes.

Equation (2.6) separates into

$$(\nabla^{2}+k_{p}^{2})(\nabla^{2}+k_{t}^{2})\tau (r)=0,$$ (2.12)

where $k_{p}^{2}$ and $k_{t}^{2}$ are the roots of a bi-quadratic equation whose coefficients can be determined from eq (2.6). The quantities *k*_p_ and *k*_t_ will be referred to as the propagation parameters for the acoustic and thermal modes. Exact expressions for $k_{p}^{2}$ and $k_{t}^{2}$ can be obtained from the bi-quadratic equation. The following series expansions of the exact solutions are, however, more useful:

$$k_{p}^{2}={\left(\omega /c\right)}^{2}\left\{1-\left(i/2\right){\left(\omega /c\right)}^{2}\left[\left(\gamma -1\right)\delta_{t}^{2}+\delta_{v}^{\prime 2}\right]+O{\left(\omega \delta /c\right)}^{4}\right\}$$ (2.13)

$$k_{t}^{2}=\left(-2i/\delta_{t}^{2}\right)\left\{1+\left(i/2\right){\left(\omega /c\right)}^{2}\left(\gamma -1\right)\left(\delta_{t}^{2}-\delta_{v}^{\prime 2}\right)+O{\left(\omega \delta /c\right)}^{4}\right\}.$$ (2.14)

The notation here indicates that the solutions are correct to fourth order in the ratio of any of the characteristic lengths *δ* to the wavelength of an acoustic wave.

Solutions of eq (2.12) for the temperature which are finite at the origin and radially symmetric have the form

$$\tau (r)=\tau_{p}j_{0}(k_{p}r)+\tau_{t}j_{0}(k_{t}r).$$ (2.15)

The propagation parameter for the thermal mode is approximately equal to (1−*i*)/*δ*_t_. Thus the argument *k*_l_*r* is generally sufficiently large that the asymptotic form for *j*_0_(*k*_t_*r*) can be used; the magnitude of the asymptotic form is approximately exp(*r*/*δ*_t_)/(r/*δ*_t_), which decays rapidly with decreasing *r*. The thermal wave solution is thus significant only within a few thermal penetration lengths *δ*_t_ of the shell wall. The pressure corresponding to eq (2.15) is

$$p^{\prime }(r)=\frac{\gamma \alpha }{\gamma -1}\left[\tau_{p}\left(1+\frac{\delta_{t}^{2}k_{p}^{2}}{2i}\right)j_{0}(k_{p}r)+\tau_{t}\left(1+\frac{\delta_{t}^{2}k_{t}^{2}}{2i}\right)j_{0}(k_{t}r)\right],$$ (2.16)

and the longitudinal particle velocity is given by

$$\rho cu(r)=\frac{ic}{\omega }\frac{\gamma \alpha }{\gamma -1}\nabla [F(k_{p}^{2})\tau_{p}j_{0}(k_{p}r)+F(k_{t}^{2})\tau_{t}j_{0}(k_{t}r)],$$ (2.17)

where

$$F(k^{2})\equiv 1+\left(i/2\right){\left(\omega \delta_{v}^{\prime }/c\right)}^{2}-\left(i/2\right){\left(k\delta_{t}\right)}^{2}+\left(\gamma /4\right){\left(\omega \delta_{v}^{\prime }\delta_{t}k/c\right)}^{2}.$$ (2.18)

Equation (2.13) can be inverted to give a classical Navier-Stokes dispersion relation for *ω* as a function of *k*_p_. Corrections to the Navier-Stokes dispersion relation have been derived from approximate solutions to the Boltzmann equation in the form of an ascending series in the variable *x≡*Λ*k*, where Λ is the mean free path [16]. These corrections are consistent with ultrasonic measurements in gases at low pressures [17], and if they were applied to the pressure regime of the present experiment they would be negligible. More recently, it has been argued that the scaled dispersion relation (*ω* divided by a collision frequency considered as a function of *x*) has corrections proportional to *x*^1/2^ with an amplitude on the order of (density/close-packed density)^2^ for a hard-sphere gas [18]. If the hard-sphere estimate is roughly applicable to argon, the more recent corrections are also negligible in the regime of the present work where *x* ranges from 10^−6^ to 5 × 10^−5^ and the density ranges from 3 × 10^−4^ to 6 × 10^−3^ of the density of liquid argon.

### 2.3 Boundary Conditions

There are four boundary conditions to be satisfied by radial modes at the gas-shell interface (*r=a*). First, the radial components of the gas and shell velocities must match. Second, the radial component of the shell velocity is proportional to the radial force per unit area exerted on it by the gas times an effective acoustic admittance *β*_sh_. Two additional boundary conditions deal with thermal effects at the gas-shell interface. These effects occur uniformly over the boundary. In this section it will be shown that these effects lead to a single equation which determines the complex eigenfrequencies. Shell motion and thermal boundary effects shift the eigenfrequencies from the unperturbed values $f_{0n}^{0}$. Other sources of eigenfrequencies shifts, such as transducers, small openings, and imperfect geometry will be discussed in subsequent sections.

The force per unit area acting on the shell differs from the acoustic pressure by a fractional amount of order (*ωδ*/*c*)^2^, which we neglect [9]. The first two boundary conditions can then be written

$${\left(\rho cu_{r}/p\right)}_{r=a}=\beta_{\text{sh}}(\omega ).$$ (2.19)

An expression for the admittance of an idealized shell was derived in reference [9]. Several assumptions made in reference [9] differ from the conditions of the experiment. The derivation is based on the theory of elasticity for isotropic materials. It applies to a uniform spherical shell, not a composite structure fabricated from parts. The experimental shell does not have a uniform thickness. The derivation neglects mechanical coupling between the shell and its environment. (Radiation of sound from the outer boundary of the shell was included in the calculation, however, and shown to be negligible.) Despite the differences between the idealized shell and the present experimental shell, the predicted effects of shell motion are sufficiently small that an approximate calculation is adequate for the determination of *R*. [The calculation was tested by measurements of the shell’s compliance (secs. 6.8.1–6.8.3) and the frequency of its breathing mode (sec. 3.8).]

The acoustic admittance of the shell was found to be

$$\beta_{\text{sh}}=[-i\rho \omega ca/(\rho_{\text{sh}}c_{\text{sh}}^{2})]S_{0}(k_{\text{sh}}a),$$ (2.20)

where *ρ*_sh_ is the shell density, *c*_sh_ is the speed of longitudinal waves in the bulk shell material, *k*_sh_ = *ω*/*c*_sh_, and the function *S*_0_ is

$$S_{0}=-q\times \frac{(1+AB-qB^{2})\tan(B-A)-(B-A)-qAB^{2}}{\left[(qA^{2}-1)(qB^{2}-1)+AB]\tan(B-A)-(1+qAB)(B-A\right)}$$ (2.21)

where the outer shell radius is *b, A* = *k*_sh_*a*, *B = k*_sh_*b*, and the parameter *q* is related to Poisson’s ratio *σ* through *q*=(1/2)(1−*σ*)/(1−2*σ*). The radial shell resonances occur at frequencies for which the denominator of eq (2.21) vanishes. The lowest such resonance will be referred to as the breathing resonance of the shell; its frequency will be designated *f*_br_. For the shell used in this work the breathing resonance occurs at approximately 13.58 kHz. All other shell resonances occur at much higher frequencies. Accordingly, an excellent approximation to eq (2.21) is obtained by taking the zero-frequency limit of eq (2.21) and dividing by the resonance term 1−(*f/f*_br_)^2^. This approximation gives

$$S_{0}=-q\frac{3qab^{2}-3bh(a-qb)-h^{3}}{3qh(a^{2}+ab+b^{2})-3abh-h^{3}}\frac{1}{1-{\left(f/f_{\text{br}}\right)}^{2}}.$$ (2.22)

The third boundary condition at the gas-shell interface deals with the temperature of the gas and the shell. In previous work, Moldover, Mehl, and Greenspan assumed that the temperatures of the gas and wall were equal at the interface [9]. However, Ewing, McGlashan, and Trusler [10] have shown that at low densities a temperature discontinuity should be included in the boundary condition. According to kinetic theory, [19–21] the temperature of the gas (extrapolated to the wall) should exceed that of the shell by

$$\Delta \tau =J_{t}l_{a}/\lambda_{g},$$ (2.23)

where *J*_t_ is the normal heat flux across the interface, *l*_a_ is the accommodation length in the gas, and *λ*_g_ is the thermal conductivity of the gas. The accommodation length is given by

$$l_{a}=\frac{\lambda_{g}}{P}\sqrt{\frac{\pi MT_{g}}{2R}}\frac{(2-h)/h}{C_{v}/R+1/2},$$ (2.24)

where *M* is the molar mass of the gas, *T*_g_ is the temperature of the gas, *R* is the gas constant, *C_v_* is the molar specific heat at constant volume of the gas, and *h* is the thermal accommodation coefficient. Ewing, McGlashan, and Trusler found that the thermal accommodation coefficient between argon and the machined aluminum wall of their resonator was 0.84±0.05. Values near unity are apparently typical for heavier gases and machined surfaces which have not been vacuum-flashed in ultra-high vacuums [20–23]. With *h* = 1, the accommodation length of argon at 273.16 K and 100 kPa is 118 nm.

For the first thermal boundary condition at the gas-shell interface, we thus assume that our equation for the gas temperature extrapolated to *r=a* must equal the shell temperature at *r=a* plus the temperature jump, or

$$\tau_{p}j_{0}(k_{p}a)+\tau_{t}j_{0}(k_{t}a)=\tau_{\text{sh}}+\Delta \tau .$$ (2.25)

The remaining thermal boundary condition at the gas-shell interface is continuity of heat flow. The thermal current in the shell can be expressed in terms of a thermal wave in the shell. Let *λ*_sh_, *δ*_sh_, and *k*_t, Sh_ = (1 − *i*)/*δ*_sh_ he the thermal conductivity, thermal penetration length, and propagation parameter for thermal waves in the shell, respectively. Radial thermal waves in the shell should be described by a Hankel function; however, owing to the small value of *δ*_sh_ an exponential form *τ*_sh_ exp[*k*_t, sh_(*r*−*a*)] is an excellent approximation. Continuity of heat flow at the interface can be expressed as

$$-\lambda_{g}[k_{p}\tau_{p}j_{0}^{\prime }(k_{p}a)+k_{t}\tau_{t}j_{0}^{\prime }(k_{t}a)]=\lambda_{\text{sh}}k_{t,\text{sh}}\tau_{\text{sh}}.$$ (2.26)

Equations (2.25) and (2.26) give the ratio of the amplitudes *τ*_p_ and τ_t_:

$$\tau_{p}j_{0}(k_{p}a)\left[1+\left(k_{p}l_{a}+\frac{\lambda_{g}k_{p}}{\lambda_{\text{sh}}k_{t,\text{sh}}}\right)\frac{j_{0}^{\prime }(k_{p}a)}{j_{0}(k_{p}a)}\right]-\tau_{t}j_{0}(k_{t}a)\left[1+\left(k_{t}l_{a}+\frac{\lambda_{g}\delta_{\text{sh}}}{\lambda_{\text{sh}}\delta_{t}}\right)\frac{j_{0}^{\prime }(k_{t}a)}{j_{0}(k_{t}a)}\right].$$ (2.27)

The final step in obtaining an equation for the eigenfrequencies is to substitute eqs (2.16) and (2.17) into eq (2.25), and to use eq (2.27) to eliminate the ratio of the temperature amplitudes. As in reference [9], it is possible to do this without introducing any approximations, which leads to an equation which can be solved numerically to determine the eigenfrequencies. For the range of parameters used in this work, however, it is possible to identify certain small terms and to obtain a sufficiently accurate approximate equation for the eigenfrequencies. The result is

$$-i(\omega a/c)\beta_{\text{sh}}=\frac{k_{p}aj_{0}^{\prime }(k_{p}a)}{j_{0}(k_{p}a)}+\frac{\omega^{2}a^{2}}{c^{2}}\left[-(1-i)\frac{\gamma -1}{2}\frac{\delta_{t}}{a}+(\gamma -1)\frac{l_{a}}{a}-(1+i)\frac{\gamma -1}{2}\frac{\delta_{\text{sh}}}{a}\frac{\lambda_{g}}{\lambda_{\text{sh}}}\right],$$ (2.28)

where products of various “small” quantities have been omitted. Numerical values of the “small” quantities are given below for argon at 273.16 K in a stainless steel resonator. The quantities are all dimensionless; frequency and pressure dependence is indicated in terms of the dimensionless frequency $\tilde{f}$ and the dimensionless pressure $\tilde{p}$.

$$\begin{array}{l} k_{p}l_{a}=6.0\times 10^{-6}\tilde{f}\\ (\lambda_{g}/\lambda_{\text{sh}})(k_{p}\delta_{\text{sh}})=1.4\times 10^{-6}{\left(\tilde{f}/\tilde{p}\right)}^{1/2}\\ k_{t}l_{a}=2.5\times 10^{-3}(1-i){\left(\tilde{p}\tilde{f}\right)}^{1/2}\\ (\lambda_{g}/\lambda_{\text{sh}})(\delta_{\text{sh}}/\delta_{t})=5.6\times 10^{-4}{\left(\tilde{p}\right)}^{1/2}\\ \delta_{t}/a=5.4\times 10^{-4}{\left(\tilde{f}\tilde{p}\right)}^{-1/2}\\ {\left(\omega \delta_{t}/c\right)}^{2}=5.8\times 10^{-6}(\tilde{f}/\tilde{p}) \end{array}$$ (2.29)

The expression in eq (2.28) involving the spherical Bessel functions is also “small.” In obtaining eq (2.28) the products (δ_t_/*a*)(*k*_t_*a*) and (δ_t_/*a*)[(λ_g_/λ_sh_)(*k*_p_δ_sh_)] were retained; all other products were omitted.

The next step is to obtain an approximate solution to eq (2.28). Equation (2.13) gives a relation between *k*_p_ and *ω* correct to order (*ω*δ/*c*)^4^:

$$k_{p}a=(2\pi a/c)(f_{0n}+\Delta f-ig_{\text{bulk}})=z_{0n}+\Delta ka,$$ (2.30)

where *f*_0_*_n_ = cz*_0_*_n_*/(2*πa*), and

$$g_{\text{bulk}}=\frac{\pi^{2}f^{3}}{c^{2}}\left[(\gamma -1)\delta_{t}^{2}+\frac{4}{3}\delta_{v}^{2}+\frac{\eta_{b}}{\rho \omega }\right]$$ (2.31)

can be identified as the contribution to the imaginary part of the resonance frequency which is proportional to the bulk attenuation of sound. In eq (2.31) and similar equations which give the value of small terms, *f* represents either the mean source frequency in steady-state measurements or the real part of the mode frequency in free decay. As noted above, the bulk viscosity term *η*_b_ is negligible for monatomic gases. By expanding the spherical Bessel functions in eq (2.28) and using the notation of eq (2.30), approximate solutions for the complex eigenfrequencies $F_{0n}=f_{0n}^{0}+\Delta f_{0n}+ig_{0n}$ can be found:

$$\begin{array}{l} \frac{\Delta f_{0n}+ig_{0n}}{f_{0n}}=\frac{g_{\text{bulk}}}{f}+\frac{\Delta f_{\text{sh}}}{f}+\frac{\Delta f_{t}+g_{t}}{f}=i{\left(\pi f/c\right)}^{2}[(\gamma -1)\delta_{t}^{2}+(4/3)\delta_{v}^{2}]+\frac{i\beta_{\text{sh}}}{z_{0n}}\\ -(1-i)\frac{\gamma -1}{2}\frac{\delta_{t}}{a}+(\gamma -1)\frac{l_{a}}{a}+(1+i)\frac{\gamma -1}{2}\frac{\delta_{\text{sh}}}{a}\frac{\lambda_{g}}{\lambda_{\text{sh}}}. \end{array}$$ (2.32)

The first term on the second line is the bulk loss term. The second is the shell correction. The sum of the remaining terms is the thermal boundary correction. The third term is the usual thermal boundary layer term. The fourth term accounts for the discontinuity of temperature at the boundary. The last term accounts for the penetration of the thermal wave into the shell.

The effect of lack of smoothness of the shell surface on the thermal boundary layer has been considered in some preliminary modeling calculations [24]. The surface was assumed to have a sinusoidal profile of amplitude *d* ≪δ_t_. (Such a finish might be left by machine tools.) The results seem to be sensitive mainly to the amplitude *d* and not to the horizontal spacing of the surface undulations, at least over the reasonable range of surface profiles which were investigated. The calculations suggest that the magnitude of Δ*f*_t_ is increased by a fractional amount of order *d*/δ_t_ and that *g*, is not affected.

### 2.4 Imperfect Spherical Geometry

Consider a resonator whose shape differs from a perfect sphere by an amount of order ϵ. Let the surface be described by

r(θ,ϕ)=a[1−ϵf(θ,ϕ)], (2.33)

where *f*(*θ*,ϕ) is a smoothly-varying function of order unity. Greenspan [5,25] showed that constant- volume shape deformations do not affect the frequencies of radial modes to order *ϵ*. His argument was based on the Ehrenfest adiabatic principle and on an exact calculation for spheroidal deformations. Mehl [6,8] later applied boundary- shape perturbation theory to radial and non-radial modes. He confirmed that the effect of geometry on radial modes can be described to lowest-order in ϵ by

$$\Delta f_{\text{geom}}/f_{0n}=C_{n}\epsilon^{2},$$ (2.34)

and calculated values of the constants *C_n_* for some sample shape functions *f*(*θ*,ϕ). The results suggest that, for the values of ϵ obtainable with high-quality machining, the frequencies of the first seven radial modes will not be shifted by geometric effects by more than one part in 10^6^. The internal consistency of experimental values of the speed of sound determined with different modes is an experimental check on this effect.

In summary, boundary shape perturbation theory predicts that the frequencies of the radial modes of a set of resonators with a common volume *V* are all the same to order ϵ. The results of an experiment done in a resonator with volume *V* will be equivalent to results obtained in an experiment with a perfect sphere of radius *a*, provided that

$$a={\left[3V/(4\pi )\right]}^{1/3}.$$ (2.35)

The volume, and hence the mean radius, of our resonator was determined by filling it with a measured quantity of mercury.

### 2.5 Transducers

The mechanical boundary impedance differs from the value for a uniform shell on the surface of the source and detector transducers. The two transducers have nominal resonance frequencies of 40 kHz. All acoustic measurements were taken at sufficiently low frequencies that the motion of the transducer membranes was limited by stiffness. Let the transducers have area *A*_tr_ and compliance per unit area χ. The acoustic admittance of the transducers is thus

$$\beta_{\text{tr}}=i\omega \rho c\chi$$ (2.36)

at low frequencies. The additional frequency perturbation due to the transducers can be calculated using boundary perturbation theory [9,26]. The result is

$$\frac{\Delta f_{\text{tr}}}{f_{0n}}=\frac{i\beta_{\text{tr}}}{z_{0n}}\frac{2A_{\text{tr}}}{4\pi a^{2}}=-\frac{\rho c^{2}\chi A_{\text{tr}}}{2\pi a^{3}}.$$ (2.37)

We used transducers with a nominal value of χ (specified by the manufacturer) of 1.5×10^−10^ m/Pa. The corresponding fractional shift is $-0.16\times 10^{-6}\tilde{p}$.

### 2.6 Openings in Resonator Wall

Owing to imperfect fit, there are small annular slits surrounding each microphone adapter. The slits have widths *d*_slit_ on the order of 10 μm, and lengths equal to the circumference of the adapters 2*πa*_tr_ ≈ 2.98 cm. According to boundary perturbation theory, if the slits have an acoustic admittance *β*_slit_, the frequency perturbations are

$$\frac{\Delta f_{\text{slit}}+ig_{\text{slit}}}{f_{0n}}=\frac{i\beta_{\text{slit}}}{z_{0n}}\frac{4\pi a_{\text{tr}}d_{\text{slit}}}{4\pi a^{2}}\approx 1.3\times 10^{-6}\frac{i\beta_{\text{slit}}}{\tilde{f}},$$ (2.38)

where the numerical value applies to a 10 μm slit width. Trusler [27] has calculated the input admittance of a slit bounded by semi-infinite parallel flat surfaces. For a slit of depth *D*, rigidly terminated at the end, the result is

$$\beta_{\text{slit}}=\frac{(1+i)\sqrt{3\gamma }}{6\delta_{v}/d_{\text{slit}}}\tanh[(1+i)(\delta_{v}/d_{\text{slit}})kD\sqrt{3\gamma }].$$ (2.39)

With a depth of 8.7 mm, typical numerical values for the real and imaginary parts of this expression range from 0.05 to 1.

### 2.7 Steady State Response

The steady state response of the resonator has been calculated using a Green’s function technique. A source region *S*′ on the inner surface of the shell is assumed to have a radial velocity *u*_s_ relative to the rest of the shell. The steady-state acoustic pressure at any point *r*′ in the resonator is

$$p^{\prime }(r^{\prime })=\sum_{N}\frac{i\omega \rho c^{2}\Psi_{N}(r^{\prime })}{V\Lambda_{N}(\omega^{2}-F_{N}^{2})}\int_{S^{\prime }}\Psi_{N}(r)u_{s}(r)dS,$$ (2.40)

where

$$\Phi_{N}(r)\approx j_{l}(k_{ln}r)Y_{lm}(\theta ,\phi )$$ (2.41)

is a general eigenfunction, *F_N_* is the corresponding eigenfrequency, Λ*_N_* is the average value of $\Phi_{N}^{2}$ over the resonator volume, and *N* stands for the triplet (*l, n, m*). The detector is typically a pressure transducer whose complex output voltage *u* + *iv* is proportional to *p*′(*r*′), and is hence proportional to the summation in eq (2.40). In normal experimental practice, only one or a small number of modes whose eigenfrequencies lie within a small range are excited. The contribution of the excited modes can be described in detail by including one or a small number of terms in the summation in eq (2.40). The remaining terms of the summation can be approximated using a Taylor series in frequency. The detector output can then be written

$$u+iv=\sum_{N}\frac{ifA_{N}}{\left(f^{2}-F_{N}^{2}\right)}+B+C(f-f_{0}),$$ (2.42)

where *A_N_, B*, and *C* are complex constants, and the sum is now over only one mode or over a small number of modes of interest.

### 2.8 A Working Equation for Determination of *R*

A “working equation” is useful for analysis of the various limits on determination of the gas constant *R* from measurements of the speed of sound. The speed of sound can be expressed as a virial series in the pressure

$$c^{2}=A_{0}+A_{1}P+A_{2}P^{2}+A_{3}P^{3},$$ (2.43)

where we have truncated the series above the last marginally significant term. The first term is

$$A_{0}=\gamma_{0}RT/M$$ (2.44)

where *γ*_0_ is the ratio of the specific heat capacities in the ideal-gas limit. For monatomic gases it has the exact value 5/3 at temperatures where electronic excitations are not important. The other coefficients are temperature dependent; they can be related to the coefficients of a volumetric virial series and the temperature derivatives of those coefficients. In this work *A*_1_ and *A*_2_ will be determined in the experiment; a value of *A*_3_ determined experimentally elsewhere will be used [28]. With approximate values to show the magnitudes of the various terms, eq (2.43) is

$$c^{2}=A_{0}[1+2.37\times 10^{-4}\tilde{p}+5.6\times 10^{-6}{\tilde{p}}^{2}+1.5\times 10^{-8}{\tilde{p}}^{3}].$$ (2.45)

Experimental determinations of the resonance frequencies can be related to the speed of sound through

$$f_{0n}=\frac{cz_{0n}}{2\pi a}+\Delta f_{\text{sh}}+\Delta f_{t},$$ (2.46)

where only the major corrections due to shell motion and the thermal boundary layer have been included. This can be rearranged to give experimental values of *c*^2^:

$$\begin{array}{l} c_{\exp}^{2}={\left(\frac{2\pi }{z_{0n}}\right)}^{2}{\left(\frac{3V}{4\pi }\right)}^{2/3}f_{0n,\exp}^{2}\left[1-\frac{2\Delta f_{\text{sh}}}{f_{0n}}-\frac{2\Delta f_{t}}{f_{0n}}\right]\\ ={\left(\frac{2\pi }{z_{0n}}\right)}^{2}{\left(\frac{3V}{4\pi }\right)}^{2/3}f_{0n,\exp}^{2}\left[1+3.4\times 10^{-6}\tilde{p}+3.4\times 10^{-4}{\left(\tilde{f}\tilde{p}\right)}^{-1/2}-1.8\times 10^{-6}{\tilde{p}}^{-1}+2.0\times 10^{-7}{\left(\tilde{f}\right)}^{-1/2}\right]. \end{array}$$ (2.47)

For the numerical expressions in eq (2.47), the low frequency form for the effect of the shell was used; a more accurate expression including the resonance denominator was used in the data analysis.

The effects of the various terms on a determination of *R* can be obtained by combining eqs (2.45) and (2.47):

$$\begin{array}{l} R=\left(\frac{M}{\gamma_{0}T}\right){\left(\frac{2\pi }{z_{0n}}\right)}^{2}{\left(\frac{3V}{4\pi }\right)}^{2/3}f_{0n,\exp}^{2}\\ \times \left[1-2.38\times 10^{-4}\tilde{p}-5.6\times 10^{-6}{\tilde{p}}^{2}-1.5\times 10^{-8}{\tilde{p}}^{3}+3.4\times 10^{-6}\tilde{p}+3.4\times 10^{-4}{\left(\tilde{f}\tilde{p}\right)}^{-1/2}-1.8\times 10^{-6}{\tilde{p}}^{-1}+2.0\times 10^{-7}{\left(\tilde{f}\right)}^{-1/2}\right]. \end{array}$$ (2.48)

### 2.9 Discussion of Working Equation for *R*

Equation (2.48) demonstrates that, to first order, the present redetermination of *R* depends upon the accurate measurement of four quantities: the molar mass of the monatomic gas used, the thermodynamic temperature, the volume of the resonator, and a set of resonance frequencies. Upon inspection of the higher order terms in eq (2.48), it becomes evident that the redetermination of *R* is best accomplished in a limited range of pressures on the order of $\tilde{p}=1$.

If the pressure were much lower than $\tilde{p}=1$, the redetermination of *R* would depend strongly on terms which vary as ${\left(\tilde{f}\tilde{p}\right)}^{-1/2}$ and as ${\left(\tilde{p}\right)}^{-1}$. These terms are proportional to the thermal conductivity of the gas and the accommodation length, respectively. With state-of-the-art techniques, the thermal conductivity can be measured with an accuracy on the order of tenths of a percent. The accommodation length is a property of both the gas and the particular surface of our resonator: thus it must be determined by fitting the pressure dependence of the resonance frequencies. The accuracy of this procedure is limited because the signal-to-noise ratio of the measurements of $f_{0n,\exp}^{2}$ varies as ${\left(\tilde{p}\right)}^{-2}$ at pressures below $\tilde{p}\approx 1$. Furthermore, as the pressure is reduced, the problem of contamination of the gas under study becomes increasingly difficult.

If the pressure were much higher than $\tilde{p}=1$, the redetermination of *R* would depend strongly on the terms in eq (2.48) which vary as $\tilde{p}$, ${\left(\tilde{p}\right)}^{2}$, and ${\left(\tilde{p}\right)}^{3}$. These terms depend upon the virial coefficients of argon and the elastic properties of the resonator. They cannot be predicted with the necessary accuracy; thus, these terms must also be determined by fitting the pressure dependence of the resonance frequencies. As the pressure is increased, the correlations between these terms in the multiparameter fit limit the accuracy with which each term can be determined.

As discussed below, we have chosen to use other measurements to obtain the coefficients of ${\left(\tilde{p}\tilde{f}\right)}^{-1/2}$ and the very small coefficients of ${\left(\tilde{p}\right)}^{3}$ and ${\left(\tilde{f}\right)}^{-1/2}$. We have fitted our own data to obtain *R* and the coefficients of ${\left(\tilde{p}\right)}^{-1}$, $\tilde{p}$ and ${\left(\tilde{p}\right)}^{2}$. We have also chosen to carry out our measurements very near *T*_t_. Therefore, we have avoided problems associated with the imperfect knowledge of the relation between the thermodynamic temperature scale and the practical temperature scale.

## 3. Fabrication and Characterization of Resonator

In this section, we describe the manufacture and characterization of the spherical acoustic resonator. The description commences with the design, fabrication, and final finishing of two hemispherical shells. It includes a table of their dimensions. We continue by discussing the assembly of the hemispheres into a hollow spherical shell and we give a description of the fixtures and the ports used to install electroacoustic transducers into the shell. This section concludes with a description of the acoustic measurements which characterize the completed resonator. These measurements determine the frequency of the breathing mode of the completed resonator.

### 3.1 Design and Fabrication

**Figure 2:**
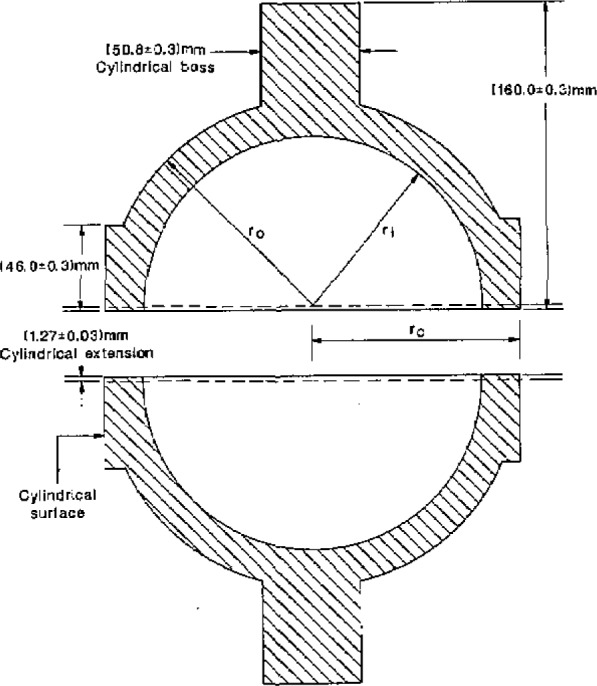
Cross-section of hemispheres.

The spherical resonator was assembled from two stainless-steel hemispheres. Figure 2 shows the hemispheres and gives the dimensions specified in our design. In earlier work with spherical resonators, the shells were fabricated from aluminium or brass. These materials are incompatible with mercury. The resonator of the present study was fabricated from type 316L stainless-steel bar stock. This alloy has excellent corrosion resistance and good machining properties.

To facilitate precise alignment of the two hemispheres, the equatorial sections of the outer surfaces were made accurately cylindrical and concentric with the spherical surfaces. Cylindrical bosses were machined at the pole of each hemisphere. These were used to hold the pieces during machining of the internal surfaces and were subsequently modified to accept mounting blocks for the two platinum resistance thermometers. The boss on the upper (“northern”) hemisphere was also machined to accommodate the gas-inlet line and valve mechanism. Previous experience had shown that polishing the interior surfaces of the hemispheres rounded their equatorial edges. This created an equatorial groove with curved sides when the hemispheres were fastened together. To prevent this, each hemisphere was initially machined with a cylindrical extension from the equatorial plane. The extension was removed after final polishing.

Two ports were machined in the northern hemisphere to accept adaptors housing the electroacoustic transducers used to excite and detect the sound field. In order to enhance the discrimination between the (3,1) non-radial mode and the (0,2) radial mode, these ports were placed 90° apart on the spherical surfaces. Their orientation at 45° with respect to the polar axis was chosen for convenience. We now describe the machining sequence required to turn the cylindrical blanks into the hemispheres as shown in figure 2, and the additional operations performed to obtain the finished resonator.

All turning operations were conducted on a numerically-controlled turret lathe. In this lathe, the turret is able to travel continuously and independently perpendicular and parallel to the turning axis. Position read-out resolution was 2.5 μm. The spindle speed was also varied under numerical control so that the surface speed of the work past the cutting tool was as constant as possible. The following operations were performed:

1. Each cylindrical blank was held in a three- jaw chuck and, after rough machining, the boss and the spherical portion of the outer surface were turned in one pass to the final size.
2. The piece was then reversed and held by the boss for the remainder of the machining.
3. The rough machining of the internal surface took place via a series of steps at 0.75 mm increments. The rough diameter was smaller than the finish diameter by approximately 1.5 mm. At this stage, the outer cylindrical surface was turned to within 0.75 mm of final size.
4. The hemispheres were then given stress-relieving heat treatment consisting of 40 hours at 400 °C in air, followed by a slow cool down over 24 hours. This operation was intended to promote dimensional stability by relieving stress originally present in the billet.
5. The stepped inner surface then received the first cut in which the cutting tool followed, in one pass, the desired profile. This profile passed along the outer cylindrical surface, across the rim, and along the inner surface of the cylindrical extension; it then proceeded along a 90° circular arc to form a hemispherical chamber. After this pass, the inner surface was within 0.25 mm of final dimensions.
6. The hemispheres were heat treated, similar to (4).
7. Prior to final machining of the spherical surface, the tool piece was reground. Since the cutting tool traced out a quarter circle cutting arc, the point of contact between it and the work piece also moved by 90°. Consequently, the tool tip was required to have an accurate semi-circular cross-section. The Stellite tool was ground to a tip radius of 0.18 mm; the radius and shape were then checked using a shadow-graph projector.
8. Each hemisphere was then chucked, trued, and given the final cut. In each case, the cutting tool followed precisely the same path, thereby ensuring nearly identical hemispheres.

### 3.2 Polishing

Visual inspection of the internal surfaces after initial machining showed that the finish was unsatisfactory. Turning marks were evident. Thus, the hemispheres were mounted in a lathe and hand polished to a near mirror finish using successively lighter grades of emery paper and cutting oil in the initial stages of polishing. The final finish was achieved using *α*- and *γ*-alumina paste (0.3 and 0.05 μm particle sizes). After these steps, almost all of the tooling marks that had initially been present were no longer visible.

### 3.3 Final Machining

After completion of the polishing and dimensional measurements, final machining was performed. This work consisted of the following five operations: (1) facing and drilling of the bosses in preparation for the attachment of stainless-steel blocks containing the platinum resistance thermometers, (2) fabrication of the gas-inlet-valve port in the boss of the “northern” hemisphere, (3) fabrication of transducer ports (also in the northern hemisphere), (4) drilling of 18 bolt holes vertically through the equatorial joint so that the hemispheres could be bolted together to form the finished resonator, and finally, (5) removal of the cylindrical extensions by turning and then lapping of the mating surfaces to a local flatness of 0.5 μm.

**Figure 3:**
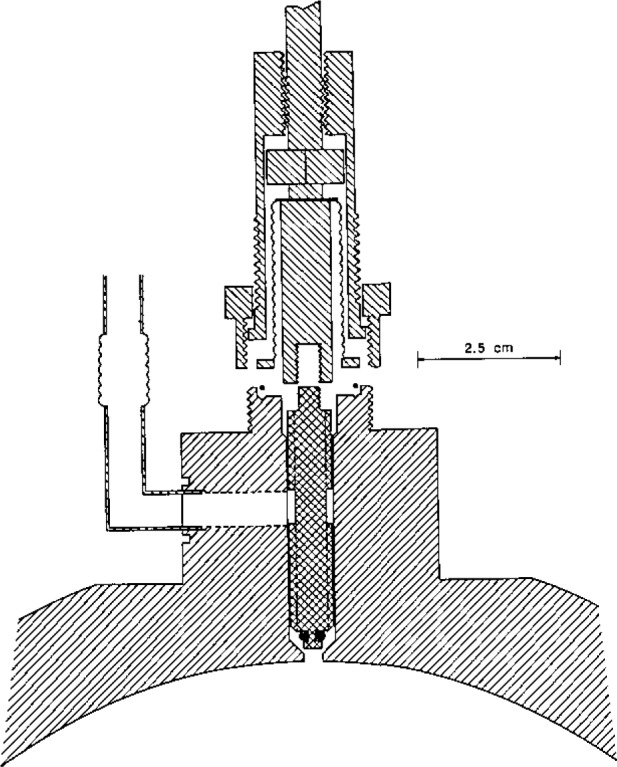
Cross-section of isolation valve.

The isolation valve is shown in detail in figure 3. It consists of the bellows assembly and valve driver mechanism from a commercial 316 stainless-steel valve (Nupro bellows valve, type SS-6BG [29]) and is clamped to the boss by a retaining nut. A vacuum tight seal is effected by the compression of a stainless-steel O-ring. The valve stem and tip supplied with the valve were removed and replaced by a specially fabricated shaft. The shaft was made from 316 stainless-steel and was polished so that there was a snug sliding fit in the passage bored in the top of the boss, ensuring that the tip of the shaft would always enter the 3.2 mm diameter aperture in the sphere wall without catching or fouling. When closed, the sloping shoulder of the stem made metal-to-metal contact with a mating surface in the passage, thus ensuring reproducible positioning of the stem tip relative to the inner spherical surfce. At the same time, a “Viton” O-ring [29] was compressed on the sloping surface and isolated the sample gas. A number of axial slits were milled along the shaft to provide pathways for gas flow when evacuating the resonator (and the dead space within the bellows). The dimensions of these slits were such that when the valve was fully open the vacuum pumping speed of the resonator was largely determined by the conductance of the aperture in the spherical wall.

In addition to having accurate placement, the transducer holes through the spherical wall must be of known depth and diameter so that the transducer housings, and the stainless steel plugs that sealed the ports during the volume determination could be machined for tight fit. Accordingly, this operation was performed using a precision jig boring machine and a micrometer-adjustable variable radius boring bar. To maintain a sharp edge, free of burrs, at the intersection of the hole and the spherical surface, cutting was performed with the rotating tool moving radially outwards from the resonator. The jig borer was also used to mill the features of the recess that located the transducer housing. These features therefore were highly concentric with the axis of the transducer port.

### 3.4 Dimensional Measurements

A Bendix [29] coordinate measuring machine (CMM) was frequently employed during machining to monitor the progress of metal removal and to determine the relationship between the cutting edge of the tool and the coordinate scales of the lathe. It was also used for the final dimensional measurements. The CMM was housed in a thermostatted room maintained at 22.07 °C. The machine had a resolution and repeatibility of 2.5 μm, and an accuracy of approximately twice that figure.

Each hemisphere was held vertically by the boss in a precision dividing-head chuck mounted on the CMM flat bed, so that coordinate measurements could be taken at different angular orientations of the hemispheres. Before commencing dimensional measurements, the hemispheres were brought into thermal equilibrium with the room. A measuring sequence consisted of: (1) determination of the coordinates of the (imaginary) plane bounding the open face of the cylindrical extension to the hemisphere from four approximately equally spaced points on the flat face of the extension, (2) co-ordinate measurements made at 12 equally spaced points around the outside of the cylindrical section, from which the least-squares-best-fit circle (center (*x*_c_,*y*_c_), radius *r*_c_) was obtained, and (3) co-ordinate measurements made at 16 equally spaced points along the arc of a great circle. Three more such arcs were traced out at 45°, 90°, and 135° to the original arc; thus there were 64 coordinate points from which the best fit spherical surface was determined. The radius *r*_s_, radial deviations δ*r*_s_, root mean square radial deviation $<\delta r_{s}^{2}{>}^{1/2}$, and coordinates (*x*_s_,*y*_s_,*z*_s_) of the center of the best fit spherical surface were then determined. This gave directly the height *h* of the cylindrical section abutting the hemisphere. In addition, the differences δ*x* and δ*y* between the centers of the spherical and cylindrical surfaces gave a measure of concentricity.

The results of dimensional measurements performed both after the initial machining and after polishing (but prior to final machining and lapping) are summarized in table 2. Repeated measurements demonstrated that the *repeatability* of mean radius determinations was about an order of magnitude smaller than the resolution of the CMM. The external radii are particularly well matched (to within 5 μm) and the difference was unchanged over the period of about 3 months between the two sets of measurements reported in table 2. The internal radii match to 20 μm, or about 220 ppm in *r*_s_. Such a difference leads to a very small, second-order, perturbation to the resonance frequencies of the radial modes used in this work. The perturbation can be calculated from table II of reference [6] and is −0.7 ppm for the (0,2) mode; it is less than 0.1 ppm for the (0,3) through (0,6) modes.

The volume of the spherical chamber that would have been formed from the two hemispheres assuming perfect removal of the cylindrical extensions is (2945.8_1_±0.5_4_) cm^3^ at 22.07 °C. This volume differs by (148±194) ppm from that obtained from weighing mercury as described in section 6. Unfortunately, we had no opportunity to monitor the removal of the cylindrical extensions using the coordinate measuring machine. Such an extension may have been the largest geometric imperfection in the assembled resonator; however, it could not have been very large without destroying the agreement of the volumes determined from dimensional measurements and from weighing.

An analysis of the deviations of the 64 data points from the best-fit spherical surface reveals that the deviations from sphericity are not random. There are two reasons why the deviations are likely to vary with latitude. Firstly, if the tool tip is not ground with a precisely constant radius of curvature, then departures from sphericity will be produced. Secondly, the surface speed past the polishing cloth depends upon the latitude; thus it is difficult to hand polish a spherical surface in a uniform way.

**Figure 4:**
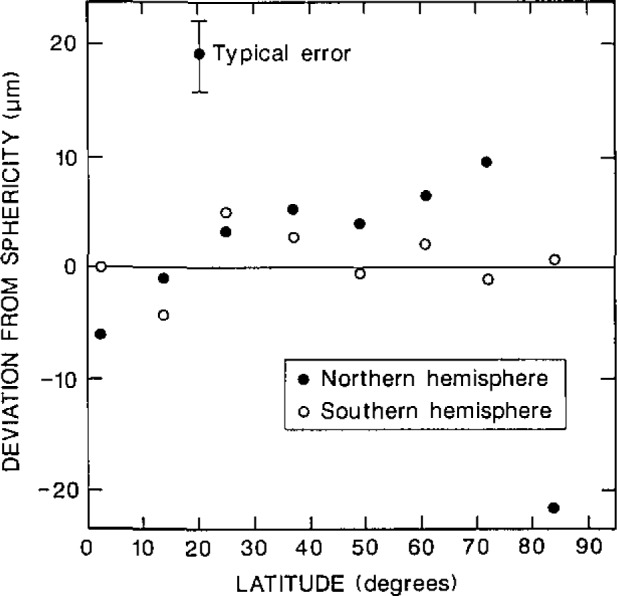
Deviations from sphericity of hemispheres after polishing.

On the assumption that the radial deviations are a function of angle alone, the 16 points on each measuring arc may be reduced to 8 mean measurements at evenly spaced latitudes between 2° and 84°. As there were 4 measuring arcs there were 8 measurements of the radial deviation at each latitude. The mean and a typical standard deviation of the 8 points at each latitude are displayed in figure 4 for both finished hemispheres.

The dimensions of the transducer ports were also measured with a calibrated coordinate measuring machine. The diameter was measured at several locations along the axis of the port. In all cases we obtained the same diameter, confirming that the transducer ports were not tapered and were circular to within the 2.5 μm resolution and repeatibility of the CMM.

The length of the transducer hole was measured by standing the hemisphere on a gage block placed within the inner recess prepared for the transducer housing. After locating the center coordinates of the port, the vertical coordinate of the gage block was measured there, and the coordinates of a number of contact points on the spherical surface were also obtained at a fixed height above the gage block. Knowing the radius *r*_s_ of the sphere and the radius of the port it is then a matter of simple geometry to obtain the length of the wall of the transducer port.

The dimensional measurements conducted on the transducer ports gave the following results:
Diameter, mmLength, mmTransducer port 1  9.545±0.0059.624±0.005Transducer port 2  9.520±0.0059.733±0.005

### 3.5 Assembly of the Resonator

In order to use the resonator for accurate measurements of the speed of sound, its volume must be stable and nearly spherical. It is particularly important to avoid a gap at the equatorial joint between the hemispheres. (Such a gap would perturb the acoustic resonance frequencies in a hard-to-predict fashion and could permit irreproducible intrusion of the mercury used for the volume measurement.) A prototype resonator had been assembled by electron-beam welding; however, it did not have a stable volume. The alternative that was ultimately used, was to “solder” the two hemispheres together with an extremely thin layer of “Apiezon-W” vacuum wax [29]. The resulting resonator had a volume which did not change by more than 0.8 ppm during the six months between two volume measurements. (0.8 ppm was the range of the volume difference measurement.) The resonator did not have a void at the joint. We shall now describe the procedure for “soldering” the hemispheres together.

The mating surfaces had been lapped flat in the optical shop to a nominal tolerance of 0.5 μm. The hemispheres had been ultrasonically cleaned and vapor degreased by the shop and finally cleaned by us with distilled trichloroethane. A solution of the wax (3 mg/cm^3^) was prepared using freshly distilled 1,1,1-trichloroethane. It was applied to one of the hemispheres using a homemade atomizer with helium as the propellant. The spraying was carried out while the hemisphere was in a fume hood and was maintained at 110 °C with a heating mantle. Under these conditions, the solvent and volatile impurities in the wax evaporated quickly, and the wax completely wetted the steel surface. Thus, the wax formed a uniform layer with no tendency to coagulate. The layer did not appear to contain embedded dust when it was examined after cooling. If there were no overspray, the 20 cm^3^ of solution would have deposited a wax layer 5 μm thick. Examination of the masks used to protect the inner surface of the hemisphere and the mantle during spraying suggests that 2/3 or more of the wax remained on the joint.

The cooled, waxed hemisphere was set on a table and the other hemisphere was lowered on it with a hoist. During this procedure, the joint was cushioned and lubricated with a layer of freshly distilled isopropyl alcohol. A coarse alignment of the hemispheres was maintained with four PTFE rods which passed through the bolt holes in both hemispheres. The final alignment was achieved manually with the aid of three aluminum blocks used at 120° intervals on the cylindrical portions of the hemispheres around the equator. The 18 stainless-steel bolts (1/4″−24) were drawn up finger tight, torqued to 1.7 N·m (15 in·lb), and then torqued to 14 N·m (10 ft·lb). The assembled resonator was heated in an oven to 60 °C and the bolts were re-torqued to 14 N·m; it was heated to 100 °C and re-torqued to 14 N·m; and, finally it was maintained at 120 °C for 15 h. After these steps, tiny beads of wax were visible at the seam on both the interior and the exterior surfaces of the resonator. (The interior was inspected with a borescope.) This indicates that the wax did flow as required and that there was no crack where the joint meets the interior surface of the resonator. We believe that the small burr that remained after lapping prevented the formation of a continuous bead at the joint. It is not surprising that we never found evidence of leakage at the joint with a helium leak detector. The tiny beads at the joint within the resonator were not removed.

### 3.6 Transducers and Transducer Housings

**Figure 5:**
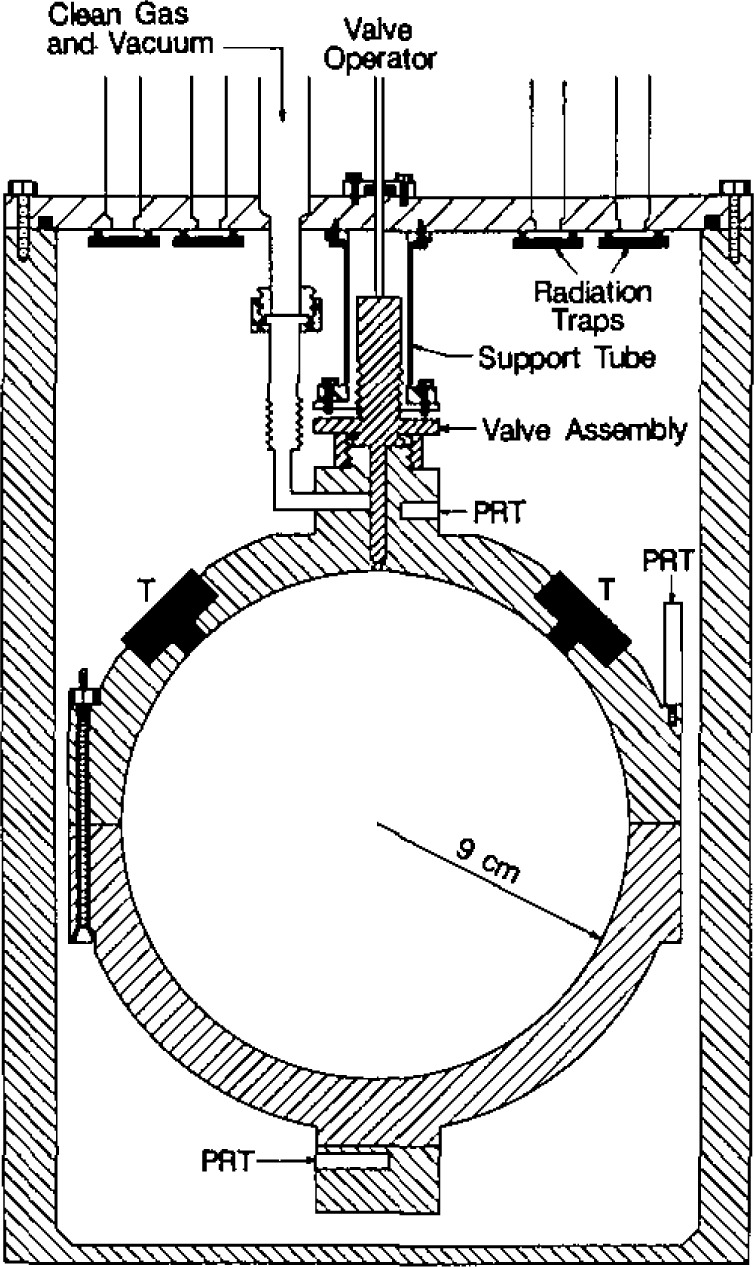
Cross-section of resonator and pressure vessel. The transducer assemblies are indicated by “T” and the locations of the capsule thermometers are indicated by “PRT.”

Two electroacoustic transducers were used to measure the resonance frequencies. Both transducers were mounted flush with the interior wall of the resonator. As sketched in figure 5, they were both located at 45° from the north pole on the same great circle through the pole. One transducer drove the sound field within the resonator and the other one detected the amplitude and phase of the pressure at a point 90° away from the driver. (This 90° orientation was chosen to minimize the detector’s response to the (3,1) non-radial mode when measuring the resonance frequency of the nearby (0,2) radially symmetric mode.) Both transducers were commercially manufactured microphone cartridges (Bruel & Kjaer Type 4135, [29]). These microphones were constructed from materials which are compatible with clean gases (silicone treated quartz, high nickel alloys, and gold plating). The manufacturer provided voluminous data describing their mechanical and electrical characteristics. The moving element of each microphone was a flat, stretched, nickel diaphragm which was nearly flush with the front of the cartridge and served as one electrode of a capacitor. The other electrode was a monel backplate located 20 μm behind the diaphragm. The generator was excited with 60 V (RMS), at half the desired acoustic frequency. The motion of the detector’s diaphragm was measured with a commercially manufactured bias supply and preamplifier (Bruel & Kjaer Model 2660, [29]), whose output was connected to a lock-in amplifier. Under typical conditions [300 kPa, (0,4) mode] the acoustic pressure was 0.0023 Pa.

**Figure 6:**
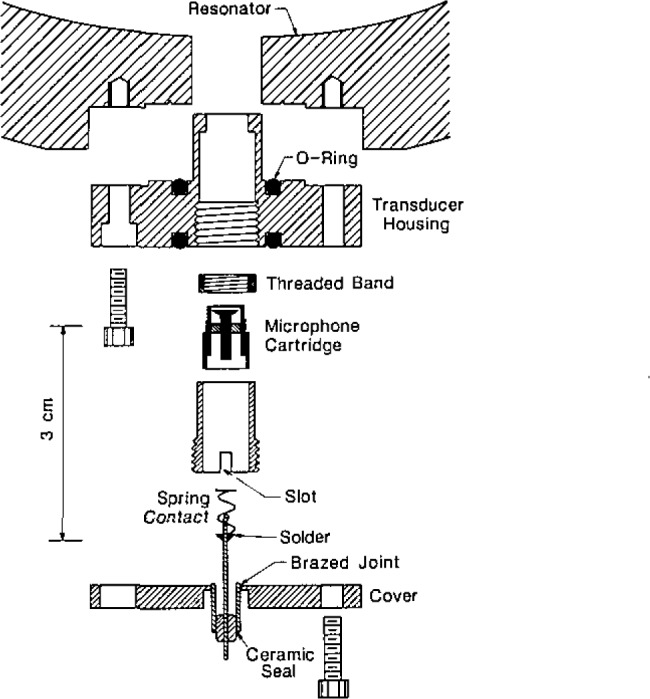
Exploded section of transducer housing assembly and electrical feedthroughs.

Transducer housing assemblies were used to support the microphone cartridges in precisely defined positions within the transducer ports of the resonator. The transducer housing assemblies also acted as hermetic seals and electrical feedthroughs. The components of a transducer housing assembly are shown in figure 6.

The manufacturer of the microphone cartridges supplied them with protective grids covering the diaphragms. These grids were unscrewed and replaced with threaded bands which were the key components for positioning the cartridges within the transducer housings. Each threaded band was pressed against the inside front surface of its housing by a tube threaded along part of its length. (A slot in the tube was used to screw the tube into the housing, thus, pressing the band against its housing’s surface.) The outside and inside diameters of the front of the housings were machined to tight tolerances (±6 μm) to minimize the volumes of the annular gaps exposed to the acoustic field within the resonator.

“Viton” [29] O-rings were used to seal the transducer housings to the resonator. Thus, the positions of the microphones’ diaphragms with respect to the interior surface of the resonator were determined by reproducible metal-to-metal contacts. The backs of the transducer housings were sealed to covers with “Viton” [29] O-rings. Commercially manufactured electrical feedthroughs were brazed to the covers. For the detector transducer, a shield (not shown) surrounded the outside of the feedthrough. Triaxial cable led from this shield and the feedthrough through the pressure vessel, up the interior of a tube through the water bath, through a triaxial feedthrough, and, with additional triaxial cable, to the preamplifier. The guard of this cable was driven by the preamplifier to compensate for loading of the detector by the large capacitance of the cable. For the driven transducer, coaxial cable led from the cover to signal source.

The volume in each transducer housing behind the microphone cartridge is approximately 0.5 cm^3^. This volume is connected to the interior of the resonator by a small pressure-equalization port within the cartridge and by the small gaps between the machined components of the transducer housing assembly. Such a volume is slow to pump out. Thus, extensive flushing procedures were used when changing the gas sample in the resonator, as described in section 7. Furthermore, to avoid stressing the diaphragms, the pressure within the resonator was never changed faster than 1 kPa/s and potential differences were never applied to the transducers while the pressure was being changed.

After their initial installation in the resonator, intermittent electrical arcing was detected in both transducers. The occurrence of arcing increased, eventually reaching several incidents within a minute. After a few hours of use, the detector transducer failed. Examination showed that its diaphragm was torn. The diaphragms of both transducers had numerous pinholes, presumably as a result of the arcing. Both damaged transducers were replaced. In subsequent use, a few isolated incidents of arcing have occurred. The transducers’ sensitivity remained unchanged after several hundred hours of operation. We have no certain explanation of the cause of the arcing, nor are we aware of any changes in procedure which have diminished its frequency.

### 3.7 Geometry of the Assembled Resonator

Moldover, Mehl, and Greenspan [9] have argued that some information concerning the geometry of the assembled resonator can be obtained by measuring the resonance frequencies of the nearly degenerate, non-radial modes. We made an effort to exploit this possibility; however, we were not able to obtain a satisfactory interpretation of the results.

We attempted to measure the three components of the (1,3) and (1,8) modes with argon in the resonator at 0.1 MPa. For both modes, only two components were found. For both modes, the two components had nearly equal amplitudes and scaled half-widths *g_N_*/*f_N_* within 3 ppm of the theoretical values. The components of the (1,3) and (1,8) modes were separated by 215 ppm and 214 ppm of their average frequencies, respectively. For both modes, the phase difference between the two components was within 0.5° of 180°. For the (1,3) mode, the phase of the lower frequency component was 8° from that interpolated between the adjacent radially symmetric (0,3) and (0,4) modes. For the (1,8) mode, the phase of the lower frequency component was 17° from that interpolated between the adjacent, radially symmetric (0,8) and (0,9) modes. Following reference [9], we attempted to interpret these observations in terms of smooth, axisymmetric imperfections in the resonator’s geometry. (The calculations use unpublished notes and eqs (67–69) of reference [9]. Note that eq (69) of this reference should be corrected to read λ_0_ = −1.)

The phase relations suggest that the lower frequency component of each mode results from gas motion with a symmetry axis which is nearly coincident with the line running from the north pole to the south pole of the resonator. The higher frequency component resulted from gas motion with a symmetry axis which is in the plane of the equator and which is also in the plane through the transducers and the poles. If this interpretation were correct, one would expect the unobserved third components of the (1,3) and (1,8) modes to be much weaker than the two observed components because the gas motion is primarily along the axis in the equatorial plane which is perpendicular to the plane of the transducers. The frequency of the third component would be nearly coincident with the higher of the two observed frequencies and the phase of the third component would be nearly coincident with the phase of the higher frequency component. The fit to the (1,3) and the (1,8) data is not improved by the addition of such a third component.

If the geometric interpretation of the splitting were correct, and if the resonator were modeled as either a prolate spheroid or as two hemispheres separated by a short cylindrical section, the effective polar diameter of the resonator would have to be about 530 ppm greater than the effective equatorial diameter.

A 530 ppm difference in diameters is not consistent with the dimensional measurements made on the hemispheres prior to their assembly (see sec. 3.4). In particular, with the assumption that there is no cylindrical extension, the volume of the resonator determined from dimensional measurements is (150±190) ppm smaller than the volume determined from the very accurate weighing and expansion measurements described in section 6. A cylindrical extension of 530 ppm would increase the volume determined from the dimensional measurements by 790 ppm leading to an inconsistency. (As discussed above, the thickness of the equatorial joint is less than 20 ppm of the diameter.) Furthermore, a cylindrical extension (or comparable ellipsoidal deformation) would cause a shift in the frequencies of the (0,*n*) modes in the second order of perturbation theory, that would range from 0.16 to 2.48 ppm for the (0,2) through the (0,6) modes. Such shifts would be manifest as a dispersion of 4.6 ppm in the values of *c*^2^ determined from these modes. No dispersion was observed. (See sec. 9 and especially fig. 17.)

### 3.8 Breathing Motion of the Shell

The effects of shell motion on the gas resonances have been discussed in reference [9] and in greater detail by Mehl [7]. In these publications, the shell motion was modeled as an isotropic “breathing” motion excited by radially symmetric oscillations of the gas within it. This model can be combined with the known dimensions of the resonator and the properties of the alloy it was fabricated from (see table 3) to predict that the frequencies of the gas resonances are perturbed by:

$$\frac{\Delta f_{\text{shell}}}{f}=\frac{-\gamma_{0}p\chi_{s,i}/3}{1-{\left(f/f_{\text{breathing}}\right)}^{2}}.$$ (3.1)

Here, *γ*_0_ is 5/3, *a* is the inner radius of the shell, χ_s,i_≡ (3/*a*)·(d*a*/d*p*)_i_ is the shell’s compliance to internally applied pressure which we have measured in connection with the volume determination (sec. 6.8.3), and the *f*_breathing_ is calculated to be 13.58 kHz from the data in table 3.

When helium was in the resonator at *T*_t_, the (0,3) acoustic resonance varied from 13.450 kHz to 13.516 kHz as the pressure was increased from 75 kPa to 1003 kPa; thus this gas resonance was strongly perturbed by the shell’s motion. Upon comparing the (0,3) resonance with the others, it was obvious that the dominant shell response occurred at a frequency well below 13.450 kHz. We were led to study the shell motion further with simple auxiliary experiments.

In one experiment, a piezoelectric transducer was clamped to the boss on the bottom of the shell and was used to shake the shell. A phonograph needle was placed in contact with the shell in several different positions to detect the shell’s motion. No resonance was observed at 13.58 kHz; however, at least three resonances occurred in the range 13.1–13.3 kHz. When the positions of the transducers were changed, the frequencies of the peak amplitudes varied slightly while the relative amplitudes varied dramatically. When the excitation was at the south pole and the detector was at the north pole, the most prominent component of this multiplet was at 13.220 kHz at 22.5 °C and at 13.190 kHz when the shell was warmed to 29.5 °C. At both temperatures, the half-width of this component was 18 Hz. While these measurements were conducted, the shell contained argon at a pressure near 0.1 MPa and the exterior of the shell was exposed to the ambient air.

**Figure 7:**
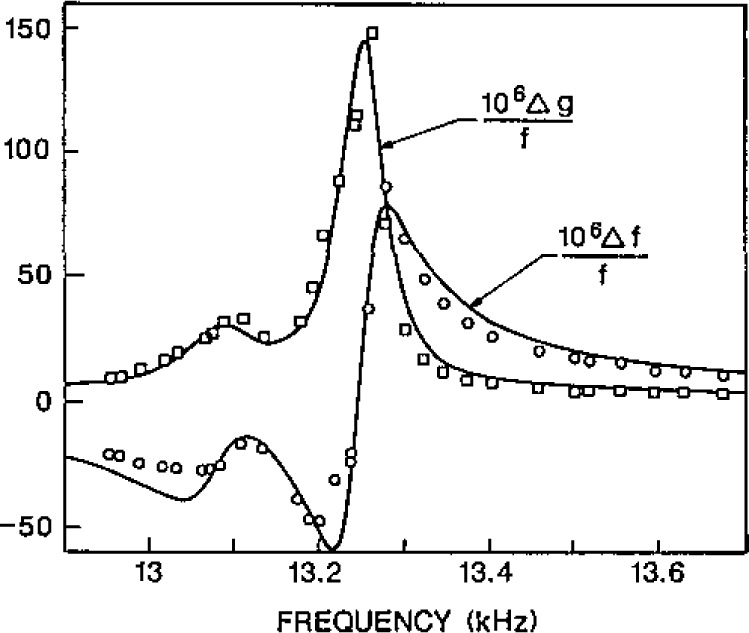
Perturbations to the frequency and half-width of (0,8) mode as a function of frequency, with argon in the resonator at 100 kPa. The frequency was swept by changing the temperature of the resonator, which changes the speed of sound.

A second experiment was conducted to determine which, if any, of these shell resonances couple to the radially symmetric oscillations of the gas within the shell. The frequencies and half-widths of the (0,2) and (0,8) modes were measured with argon in the resonator while the speed of sound of the argon was continuously changed by scanning the temperature of the shell from 0–35 °C. To interpret the results, it was assumed that the effect of the shell motion on the (0,2) mode is exactly given by eq (3.1). The measured perturbation of the real and imaginary parts of the (0,8) frequency is shown in figure 7. The smooth curve drawn through the data is the sum of two terms in the form of eq (3.1) with “*f*_breathing_” given by 13245 + 30*i* Hz and 13080+50*i* Hz and with amplitudes 0.3 and 0.08 times as large as that in eq (3.1). It is clear that at least two of the shell resonances which were directly observed do indeed couple to radially symmetric gas motion. In order to account for the measured static compliance of the shell, one must assume that there are other resonances which also couple to radial gas motions. They might be nearly degenerate and interfere with the observed resonances, or they might be too broad to be detected by these simple experiments.

The shell’s motion is more complex than an isotropic breathing. In lieu of a more accurate model, we have not based the measurement of *R* on data close to the predicted breathing mode. (The argon data for the (0,2) through (0,6) modes span the range 2.5–10.0 kHz.) We have used eq (3.1) with *f*_breathing_=13.58 kHz to approximate the effects of shell motion. If this approximation were seriously in error, the values of *c*^2^ determined with various modes would differ significantly from one another. They do not. If *f*_breathing_ in eq (3.1) were changed by 400 Hz, *R* would change by less than 1 part in 10^8^. If the compliance *χ*_s,i_ were changed by its estimated error, 6%, *R* would be changed by less than 1 part in 10^8^. Because the frequency shifts produced by the shell’s motion are a linear function of pressure, the errors in *χ*_s,i_ have a direct effect on *A*_1_, the first pressure coefficient of the square of the speed of sound. A 6% increase in the magnitude of *χ*_s,i_ leads to a 0.09% increase in *A*_1_.

## 4. Measuring Resonance Frequencies

We shall now describe the procedures we used for measuring the frequencies and the half-widths of the acoustic resonances. Upon assessing the accuracy of the results we shall argue that the data for the (0,7) mode should be ignored when either helium or argon is used in our resonator because it overlaps the (13,2) mode. As discussed above, the (0,3) mode should be ignored when the resonator is filled with helium because this mode is nearly coincident with the breathing resonance of the empty shell.

### 4.1 Procedures for Frequency Measurements

The strategy used to measure the resonance frequencies and the half-widths *g_N_* is the same one documented in earlier work [9,11]. Preliminary measurements were used to estimate *f_N_* and *g_N_*. Then the drive transducer was stepped through 11 synthesized, discrete frequencies starting at *f_N_−g_N_* and increasing in increments of *g_N_*/5 until *f_N_+g_N_* was reached. At each frequency, the in-phase voltage *u* and the quadrature voltage *v* produced by the detector transducer were measured with a tracking lock-in amplifier, scanner, and digital voltmeter, all operating under control of a microcomputer. Then the sign of the frequency increment was reversed and the voltages were measured again as the frequency was reduced in steps back to its original value. A function of the theoretically predicted form

$$u+iv=\frac{ifA}{\left(f^{2}-F_{N}^{2}\right)}+B+C(f-f_{N})$$ (4.1)

was fit to the 11 frequencies and 44 voltages. Here, *A, B*, and *C* are complex constants, and *F_N_*=*f_N_*+*ig_N_* is the complex resonance frequency of the mode under study. The parameters *B* and *C* account for possible crosstalk and for the effects of the “tails” of the modes other than the one under study. For all the argon data at pressures above 100 kPa, the inclusion of the parameter *C* in eq (4.1) is justified at a 95% confidence level by the *F* test for the statistical significance of the reduction in χ^2^. Its omission changes the mean value of *f_N_* by 0.1 ppm at 500 kPa and by 0.5 ppm at 100 kPa. The efficient algorithm used for fitting eq (4.1) to the data has been described elsewhere [30].

The dwell time at each frequency prior to the voltage measurements was the longer of 1.2/*g_N_* and 3.2 s. The former time was required for the sound field in the resonator to settle sufficiently close to its steady state and the latter time was eight times the post-detection time constant of the lock-in amplifier. The settling time for the frequency tracking circuitry of the lock-in amplifier was less than 0.4 s.

### 4.2 Random Errors of Resonance Frequency Measurements

When the resonator is filled with argon at a pressure *p*, and the post-detection time constant is 0.4 s, an approximate expression for the standard deviation of a measurement of *f_N_* is:

$$\sigma (f_{N})=10^{-7}f_{N}\cdot \left\{1+{\left(100\;\text{kPa}/p\right)}^{2}{\left(6\;\text{kHz}/f_{N}\right)}^{2}\right\}.$$ (4.2)

At pressures above 100 kPa the signal-to-noise ratio (*s/n*) is sufficiently high that the imprecision of a measurement is dominated by small, uncontrolled phase shifts in the measurement system (probably in the lock-in amplifier). The loss of precision at low pressures can be understood from the following considerations: The imprecision of a measurement of *f_N_* and *g_N_* is, within a factor of order unity, *g_N_*/(*s/n*) where *s/n* is the signal-to-noise ratio of a measurement of the acoustic pressure at the detector transducer. Under the conditions of these measurements, the source transducer is not heavily loaded. It generates an acoustic pressure *k*·*p*·*Q*, where *k* is a proportionality factor, *p* is the ambient pressure and *Q=f_N_*/(2*g_N_*) is the quality factor of the resonance under study. Because *g_N_* is dominated by the thickness of the thermal boundary layer, it varies as *p*^−1/2^. We have:

$$\frac{\delta f_{N}}{f_{N}}\approx \frac{g_{N}}{f_{N}(s/n)}=\frac{g_{N}}{f_{N}}\left(\frac{n}{kpQ}\right)=\frac{n}{2kpQ^{2}}\approx \frac{2n}{f_{N}^{2}kp^{2}}.$$ (4.3)

The effect of the signal declining as *p*^1.5^ and the effect of the resonance half-widths increasing as *p*^−0.5^ conspire to reduce the frequency resolution as *p*^−2^. At low pressures, the integration time was increased somewhat; however, at 25 kPa the standard deviation of a typical frequency measurement was 1 ppm. When the resonator is filled at some pressure *p* with helium instead of argon, the transducer’s characteristics are essentially unchanged [31]. The *Q* of any resonance is the same as the resonator would have had if it were filled with argon at the pressure *p*/2.7. One expects eq (4.3) to give the standard deviation of a measurement of *f_N_*, provided that the characteristic pressure in that equation is replaced with 270 kPa. This expectation is confirmed by the data.

### 4.3 Systematic Errors in Resonance Frequency Measurements

We consider several possible sources of systematic errors in the procedures for the measurement of the resonance frequencies. We show that systematic errors arising from the frequency standard, nonlinear effects, and the instrumentation for frequency measurement are negligible. The evidence presented in section 4.3.4 shows that the data for the (0,7) mode must be rejected because this radially symmetric mode happens to overlap a neighboring non-radial mode.

#### 4.3.1 Frequency Standard

Before and after the measurements reported here, the frequency synthesizer was compared with a standard oscillator which in turn is frequently compared with the signals broadcast by WWV. The comparison revealed that the oscillator within the synthesizer had a frequency 0.20 Hz higher than 10 MHz. The tabulated frequencies have been corrected accordingly.

#### 4.3.2 Nonlinear Effects

To minimize possible electrical crosstalk, the drive transducer was not operated with the usual dc bias voltage. Instead, it was supplied with an ac voltage (typically 60 V RMS) at half the desired acoustic frequency. This voltage was obtained by passing the output of the synthesizer through a transformer. Under typical operating conditions (300 kPa, argon, (0,4) mode) the acoustic pressure at resonance was 0.03_5_ Pa. The acoustic pressure at resonance varied quadratically with the drive voltage as one would expect with an unbiased drive transducer.

We have searched for possible systematic errors in the measurement of the resonance frequencies resulting from nonlinear behavior of the resonator, and/or the instrumentation and procedures of data acquisition and analysis. The resonance frequencies were unchanged (Δ*f/f*=−0.06±0.28 ppm) when the drive voltage was reduced by at least a factor of 3.2 thereby reducing the acoustic pressure by at least a factor of 10. These searches were made at pressures in the range 0.1–1.0 MPa and at the highest and lowest frequencies used and with helium and argon in the resonator.

#### 4.3.3 Ring-Down Experiment

In previous work [9], an important check was made of the data acquisition system and the numerical methods used to fit eq (4.1). A 1-liter resonator was filled with propane at 287.5 K and 0.52 MPa. Under those conditions, the (0,2) mode occurred at 2568 Hz and had a half-width of 0.0526 Hz. The mode was excited and then the source transducer was turned off. The detected voltage was measured as a function of time during the “ring-down.” The value of *g*_0, 2_ determined by fitting an exponential function to the voltage differed from the value determined by the cw methods described above by only 0.0004 Hz or 1.6×10^−7^*f*_0, 2_. This small difference is consistent with the limitations of the instrumentation mentioned above.

#### 4.3.4 Overlapping Modes

The original design for this determination of *R* included plans to measure the resonance frequencies of the six lowest radially symmetric modes (i.e. (0,*n*) with *n* = 2,3,…, 7). A complete set of data for these modes has been obtained; however, we shall now argue that the overlap between the (0,7) mode and the adjacent (13,2) mode is so great that inclusion of the data for the (0,7) mode in the final analysis would lead to a systematic error in *R* and an overestimate of the inconsistencies in the acoustic measurements. The problem is a consequence of the fact that the (13,2) mode is comprised of 27 partially resolved components; thus, its contribution to the detected signal is not a linear function of frequency on the scale of *g*_0, 7_ and eq (4.1) is not a satisfactory representation of the data. Because the widths of the resonances increase as *p*^−0.5^, the problem of overlapping modes is aggravated at low pressures.

**Figure 8:**
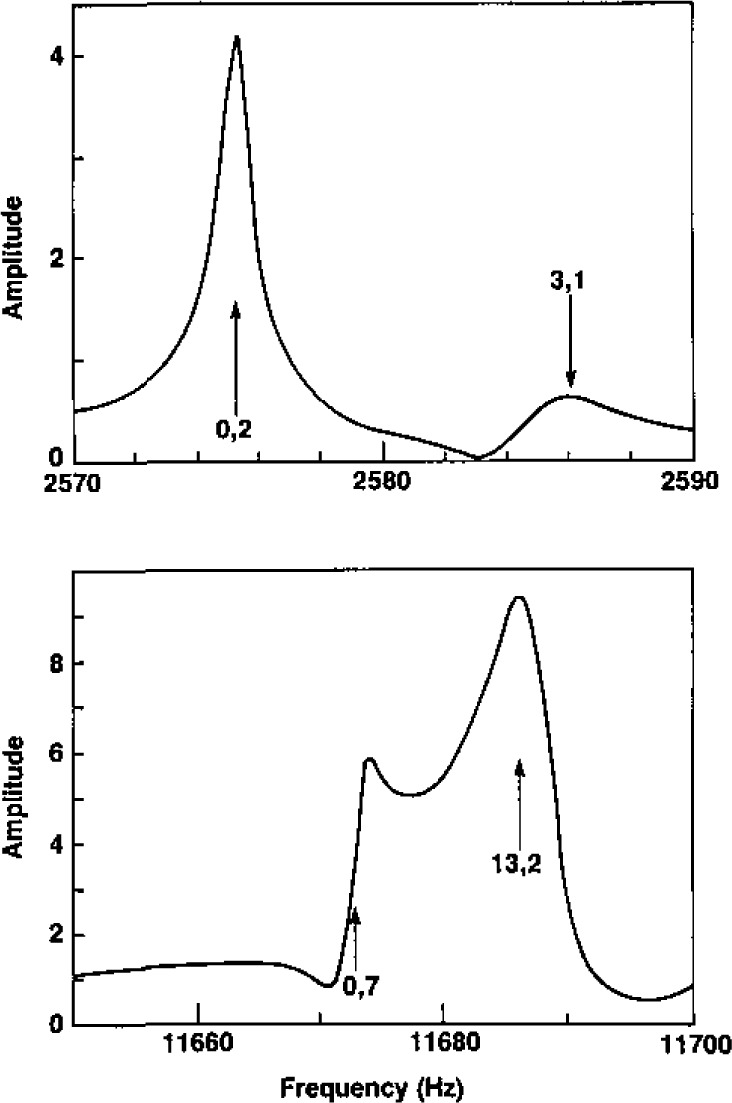
Relative amplitude of the acoustic pressure as a function of frequency in the vicinity of the (0,2) and (0,7) modes.

Figure 8 displays the amplitude of the acoustic pressure as the source transducer is swept through frequencies in the vicinity of the (0,2) and (0,7) modes. These data were taken with the resonator near 295 K and filled with argon at 100 kPa. (If the resonator were filled with helium at 270 kPa, the figure would be unchanged, except for a scale factor for the abscissa.) It is clear that the (0,7) mode overlaps the (13,2) mode; the (0,2) mode is better resolved from the (3,1) mode. The (0,3)–(0,6) modes are not displayed here; however, they are even better resolved from neighboring modes.

When eq (4.1) is fitted to the data for the (0,7) mode, there are three indications of problems. First, the value obtained for *F*_0, 7_ changes greatly when the number of fitting parameters is changed from 3 to 4 complex numbers (i.e., whether or not the constraint *C*=0 is imposed). In the case illustrated in the upper part of figure 8 (argon, 100 kPa), *F*_0, 7_ changes by (−4.1–4.5*i*) ppm upon changing from 3 to 4 complex parameters. In contrast, the changes for *F*_0, 2_ through *F*_0, 6_ are much smaller: (1.0+0.9*i*) ppm, (0.7+0.7*i*) ppm, (0.2+1.0*i*) ppm, (−0.1+1.1*i*) ppm, (−0.4+0.8*i*) ppm, respectively. A second indication of difficulty is that the values of *g*_0, 7_/*f*_0, 7_ resulting from 3 or 4 parameter fits exceed the theoretical value by 8.6 or 3.7 ppm. (For the other modes, the excess values of *g*_0_, *_s_*/*f*_0_, *_s_* are −0.1 to 1.7 ppm from 3 parameter fits; the excess values of *g*_0_, *_s_*/*f*_0_, *_s_* are 1.3 to 2.5 ppm from 4 parameter fits.) A third indication of the problem of overlapping modes is that the speed of sound determined from the (0,7) mode is not consistent with that determined from the (0,2)–(0,6) modes. For example, the speed of sound in argon at 100 kPa determined from a 4 parameter fit to the (0,7) mode is 5.7 ppm higher than the mean speed of sound determined from 3 or 4 parameter fits to the (0,2)–(0,6) modes. This 5.7 ppm difference corresponds to 6 standard deviations of the mean for 3 parameter fits to the other modes, and it corresponds to 11 standard deviations for 4 parameter fits.

From the evidence we have just reviewed, it is clear that the data from the (0,7) mode are systematically in error. Accordingly, we neglect them.

## 5. Thermometry

The temperature of the gas within the resonator was inferred from reading two 25 Ω, capsule-type, platinum resistance thermometers which were embedded in metal blocks attached to the bosses at the top and the bottom ends of the resonator. The imperfections in the measurement of the temperature of the gas contribute approximately 0.9 ppm to the standard deviation of the present determination of *R*. This includes an allowance of 0.22 mK (0.80 ppm) for imperfections in the measurement of the temperature and an allowance of 0.1 mK (0.37 ppm) for the effects of the temperature gradient that was present across the resonator during the acoustic measurements at *T*_t_ = 273.16 K. We shall now describe the factors which lead to these error estimates. We consider the resistance bridge, the history of the resistance thermometers, the thermal environment of the resonator, and miscellaneous observations which test our understanding of the temperature of the gas.

### 5.1 Thermometer Calibration, History, and Stability

The resistance bridge and the capsule thermometers together function as a transfer standard between a triple point cell and the resonator. Thus our primary concern is the long term stability of these elements. This has been established by periodically checking the thermometers in triple point cells. In table 4 we list the quantity *R* (*T*_t_, *i*→0) which is the resistance measured with the ac bridge and extrapolated to zero current at *T*_t_. The average change in *R* (*T*_t_, *i*→0) between our calibrations is equivalent to 0.22 mK. We believe that this is a sensible estimate of the errors in our temperature measurements. A standard deviation of all the measurements for each thermometer would be somewhat smaller; however, it might be a misleading measure of error for two reasons. First, the measurements show small correlated changes which plausibly could be attributed to drift in the internal standards of the resistance bridge we used. Second, there is evidence of a secular increase in *R* (*T*_t_, *i*→0) for thermometer 835B which we cannot explain. (During recalibration on April 16, 1986, this thermometer was found to be unstable. A subsequent examination showed that the glass seal of the thermometer had a crack which may have formed as it was removed from the resonator and installed in the probe used for insertion into the triple point cell. No instability was evident prior to removing this thermometer from the resonator.)

Table 5 lists values for the parameters α and δ which occur in the equations in the definition of the International Practical Temperature Scale of 1968. The values of δ for three of the thermometers were taken from calibrations made by the NBS Temperature Section in the years 1972–1978, long before the start of this project. The value of the parameter δ for thermometer 835B was assumed to be the same as δ for thermometer 1888002. The values for α were based on these values of δ in combination with the tabulated calibrations at *T*_t_ and additional calibrations of each capsule thermometer in a gallium triple point cell. Because all of the measurements of the volume and the resonance frequencies were carried out within 0.05 K of *T*_t_, the uncertainties in α and δ made negligible contributions to the uncertainty in *R*.

#### 5.1.1 Resistance Bridge

The thermometers’ resistances were measured using a 4-wire, ac resistance bridge designed by Cutkosky [32] and operated at 30 Hz. The bridge was built and tested by Robert S. Kaeser of the Temperature and Pressure Division of NBS and has been designated NBS/CAPQ Microhm Meter 5. The long term drift of the bridge arises from the drift of the internal standards and was measured at 1.7 ppm in 2 years, relative to a 10 Ω thermostatted Rosa standard. The bridge was normally operated with a measurement current of 1 mA. With our 25 Ω thermometers installed in the resonator, a typical standard deviation of a bridge reading was 3 μΩ which corresponds to 30 μK.

#### 5.1.2 Calibration Probes

For calibration, each capsule thermometer was placed in an extension probe similar to that described in Monograph 126 [33]. In the probe, the capsule is surrounded by a carefully fitted copper sleeve. The sleeve is attached with an O-ring seal to the end of a thin walled stainless steel tube designed for insertion into a triple point cell. There were appropriate extension leads and radiation shields inside the tube and a valve atop the tube permitted us to evacuate it and then backfill it with helium. The self-heating coefficients of the thermometers were measured in this configuration during recalibration of the thermometers at *T*_t_. These values are also listed in table 5. The self-heating coefficient of the thermometers changed by less than 10 μΩ/mA^2^ (0.1 mK) when the thermometers were installed in the resonator.

### 5.2 Temperature Measurements

The temperature attributed to the gas within the resonator was always calculated from the average of the temperatures indicated by the thermometers in the top and the bottom of the resonator. It was assumed that the combination of the thermometers and the bridge drifted linearly in time between calibrations. Thermometer #1888002 monitored the temperature of the top of the resonator. Thermometer 835B monitored the temperature of the bottom of the resonator until April 15, 1986. Subsequently, thermometer #1818362 was used to monitor the temperature of the bottom of the resonator. A third thermometer (#303 for the critical measurements) was mounted in a threaded copper sleeve which, in turn, was screwed into a tapped hole in the equatorial band on the resonator. A thin layer of vacuum grease was used to improve the thermal contact between the thermometers and their respective sleeves. The self-heating of the thermometers, when installed in the resonator, was within 10 μΩ/mA^2^ of that measured when the thermometers were in calibration probes.

It was found that the equatorial thermometer was perceptibly coupled to the temperature of the gas within the can (which in turn is coupled to the water bath) as well as to the temperature of the resonator. When the can was filled with argon at 0.1 MPa, the temperature indicated by the equatorial thermometer was approximately 0.94·*T* (resonator) + 0.06·*T* (bath).

### 5.3 Temperature Gradients

During the acoustic measurements used to obtain *R*, a small vertical temperature gradient existed along the resonator. (Typically, the north pole was 0.5 to 0.7 mK warmer than the south pole.) One can show that the resonance frequencies for the radially symmetric modes are determined by the volume average of the temperature distribution and that these frequencies are unaffected by the presence of a temperature gradient [34]. We believe our imperfect knowledge of the volume average of the temperature distribution is no more than 0.1 mK (relative to the calibrations of the thermometers); thus, the effect of the gradient increases the uncertainty in the measurement of *R* by 0.37 ppm. We now discuss this gradient.

As shown in figure 5, the resonator was hung from the lid of a cylindrical pressure vessel. The pressure vessel was surrounded by a carefully designed water bath. When the water bath was maintained near *T*_t_ for an extended period, a steady state developed such that the top of the resonator was 0.5 to 0.7 mK warmer than the bottom. A nearly identical gradient was measured just before and just after the thermometer calibration of April 1–3, 1986. After this calibration, the equatorial thermometer was installed and it indicated a temperature that differed from the mean of the thermometers by no more than 0.2 mK. Concurrently with the calibration, the bath was modified to greatly increase the circulation rate. The thermal grounding of the leads to the transducers, heaters, and thermometers was improved and the radiation traps on the tubes used to conduct the leads from the room through the water bath into the pressure vessel were improved. The gradient was not changed by these modifications. The gradient was unchanged when the depth of immersion of the top of the pressure vessel was increased from 5 to 11 cm and when the top of the bath was insulated. (The sides and bottom of the bath are 7 cm thick including 5 cm of closed cell foam insulation.) When the bath was maintained near the triple point of gallium (29.771 °C) the sign of the gradient reversed and its magnitude was significantly reduced. This suggests that the gradient is the result of a heat leak from the laboratory into the top of the resonator. Additional evidence that there was a genuine heat leak results from the observations that the bath had to be maintained approximately 6 mK below the temperature of the resonator to avoid temperature drifts (when the pressure vessel was filled with argon) and that when the bath and resonator were at the same temperature, the resonator’s temperature increased at the rate of 0.6 mK/h. When the pressure vessel was filled with argon, the thermal conductance between the resonator and the bath was 0.3 W/K. (2/3 of this conductance is the result of black-body radiation.) From these observations we estimate that the stray heat input was approximately 1.5 mW. We suspect that radiant heat transport down the filling tube leading from the gas manifold at room temperature to the valve on top of the resonator is responsible for this heat leak. We attempted to confirm this by heating a portion of the manifold; however, we did not see a change in the 0.2 mK gradient in an experiment which lasted 1 hour. If radiation were indeed responsible for the heat leak, the radiation would have been intercepted by the closed valve on the top of the resonator. The gas within the resonator would be affected only by the temperature gradient induced within the shell, which we have measured.

It is possible to model the temperature distribution that the resonator would acquire in a steady state using the parameters in the preceding paragraph, the dimensions of the resonator, and the known thermal conductivity of stainless steel. A straightforward calculation predicts that at *T*_t_ the equator will be 0.15 mK warmer than the south pole and that the north pole will be 0.35 mK warmer than the equator. The same model predicts the gradient will be multiplied by −1/3 at the gallium point. These model gradients are consistent with the observations at *T*_t_ and at the gallium point, leading us to believe that the volume average of the temperature of the gas within the resonator was known within 0.1 mK, relative to the thermometer calibrations.

We now mention three, negligibly small, sources of heat to the resonator. Under typical conditions used during the acoustic measurements (300 kPa, argon, (0,4) mode), the acoustic pressure at the detector transducer was roughly 0.0023 Pa and the acoustic power dissipated in the thermal boundary layer on the interior of the resonator was 2×10^−13^ W. The resistance bridge dissipated 25 μW in the capsule thermometers mounted in the resonator. This power was divided equally among the three thermometers because they were measured in a cycle which switched the bridge from one to another at 1 minute intervals. The heat conducted from the laboratory to the resonator through the electrical leads to the thermometers and transducers is estimated to be less than 1 μW because of careful thermal grounding of the leads to the lid of the pressure vessel.

### 5.4 Additional Observations Concerning Thermometry

A few hundred seconds after the valve in the filling port of the resonator was closed, the thermometer attached to the top of the resonator showed a temperature increase of 1–3 mK. This increase relaxed with a time constant of 1800 s, which is what one would expect for heat diffusion in the shell. The final temperatures indicated by the thermometers were identical with the ones before closing the valve, indicating that the top thermometer was responding to a small amount of energy supplied in its vicinity.

The thermal relaxation time of the resonator towards the bath temperature was approximately 33000 s when the resonator is within 0.1–0.01 K of the bath temperature. This is inconveniently slow. When acoustic measurements were made at several pressures with a given sample of gas, the data at the highest pressure were taken first. Then some gas was pumped out of the resonator and its surrounding pressure vessel (in such a way as to avoid large pressure differences across the resonator’s wall). As expected, the adiabatic cooling of the resonator was substantial. The resonator was then re-heated to a temperature near *T*_t_ using a 5 cm high foil heater which had been glued around the circumference of the equator for this purpose. The temperature inhomogeneities produced by this symmetrically applied heat pulse relaxed with a time constant of 720 s.

It was necessary to keep the valve in the fill port closed during the most precise acoustic measurements. Otherwise, temperature fluctuations in the room would be communicated to the gas in the manifold which in turn would cause the pressure in the resonator to fluctuate. These pressure fluctuations would be too small to change the speed of sound noticeably; however, the adiabatic temperature changes associated with the pressure changes are easily detected.

## 6. Determination of the Resonator’s Volume

### 6.1 Summary and Results of Volume Determination

In order to determine *R*, the volume of the resonator must be accurately known at temperatures near *T*_t_ = 273.16 K and at pressures in the range 0–1 MPa. The volume was measured by weighing the quantity of mercury required to fill the resonator when it was maintained at *T*_t_ and had equal external and internal pressures of *p*_0_= 101.325 kPa. Additional measurements were made of the thermal expansion of this volume and its compliance to internal and external pressures. In the following sections we shall describe the measurements, calculations, and diagnostic tests which have led to our conclusions: In the weighing configuration at *T*_t_ and at internal and external pressures of *p*_0_, the internal volume of the resonator was:

$$V_{R}(T_{t},p_{0})=(2943.1524\pm 0.0036)\;{\text{cm}}^{3}\;(1.21\;\text{ppm}).$$

At temperatures *T* between 273 and 303 **K** and at pressures *p* (equal inside and outside the resonator) between 0 and 1 MPa, the volume is given by

$$V_{R}(T,p)=V_{R}(T_{t},p_{0})[1-6.18\times 10^{-12}{\text{Pa}}^{-1}(p-p_{0})+47.6\times 10^{-6}K^{-1}(T-T_{t})]$$

with an additional variance which depends on *T* and *p*:

$${\left[0.6\times 10^{-6}{\text{Pa}}^{-1}(p-p_{0})\text{ppm}\right]}^{2}+{\left[0.1K^{-1}(T-T_{t})\text{ppm}\right]}^{2}.$$

Upon conversion of the configuration of the resonator from that used for weighing to that used for the measurement of the acoustic resonance frequencies, the volume was increased by

$$(0.0142\pm 0.0005)\;{\text{cm}}^{3}\;\text{or}\;(4.82\pm 0.17)\;\text{ppm}.$$

At *T*_t_ and *p*_0_, the uncertainty in the volume of the resonator in the acoustics configuration has important contributions from six sources: The dominant contribution is the uncertainty (1 ppm) in the integrated thermal expansion of mercury in the range 0–20 °C. The five additional contributions to the uncertainty are: the uncertainty which Cook placed on the density of NBS mercury at 20 °C (0.42 ppm), an allowance for possible density changes during storage (0.3 ppm), the random uncertainty in the average of our three volume determinations (0.29 ppm); the uncertainty in the mass of the counterweights (0.14 ppm), and the uncertainty in changing the configuration of the resonator from that used for the volume measurements to that used for frequency measurements (0.17 ppm).

### 6.2 Principles of Volume Determination

We determined the volume of our resonator from the mass of mercury required to fill it at *T*_t_. The accepted density of mercury is based on the classic work of Cook and Stone [35], who determined the mass of mercury displaced by a cube of known volume; and of Cook [36], who then measured the mass of mercury which filled a cube of known volume. The basic idea of our volume measurement is simply Cook’s second method run in reverse. To implement this idea with the required accuracy, we had to reconfigure the resonator to resemble a volume dilatometer. The valve in the north pole of the resonator was replaced with a glass capillary tube and expansion volume assembly. The electroacoustic transducers were replaced with carefully designed plugs.

Our techniques differ from Cook’s in other significant details. For instance, rather than weigh the resonator when filled with mercury (a total mass of about 60 kg), we weighed the deficit of mercury from a weighing bottle which was used to transfer mercury to the resonator. By transferring mercury to the resonator in two separate filling operations, the total mass of the filled weighing bottle was kept to less than 25 kg, thus allowing us to use a balance (described below) which has a standard deviation of less than 2 mg.

It is a fundamental assumption in this determination of *R* that liquid mercury completely filled the resonator during the determinations of its volume. We shall describe extensive tests which would have detected a bubble or a void in the mercury in the resonator if its volume were greater than 0.3 ppm of the resonator’s volume. We have also assumed that the volume of the resonator is a stable function of temperature and pressure. We expected that both the compliance and the thermal expansion of the resonator would be determined by its geometry and the properties of 316 stainless steel, without the influence of joints and seals. This expectation has been confirmed by the reproducibility of our volume measurements and by diagnostic tests described below.

The discussion of the volume determination proceeds in the following order. We recall the literature on the density of mercury. The methods we used for weighing mercury and for filling the resonator with mercury are described. The results of the weighings are presented and followed by measurements of the thermal expansion, the compliance, and diagnostic tests for bubbles and voids.

### 6.3 Density of the Mercury

Cook’s two measurements of the density of mercury differed by less than 0.5 ppm. Each measurement was subject to quite different known or suspected systematic errors; thus, the metrological community has placed great confidence in Cook’s results. Samples of mercury, directly traceable to Cook, have been used in precision manometry [37] and in the measurement of the so-called “absolute volt” [38]. The mercury we have used is also traceable to Cook. Indeed, it came from the same NBS stock described in reference [37].

Cook reported a total standard deviation for his measurement of NBS mercury as 0.42 ppm. Sloggett et al. [38] have recently conducted an extensive review of the density of mercury in connection with their redetermination of the absolute volt. They have used Cook’s results and have included an additional uncertainty component 0.3 ppm to account for possible changes in the mercury’s density during storage and manipulation. We shall do the same.

Cook’s measurements were done at a nominal temperature of 20 °C (measured on the IPTS-48 temperature scale) whereas our resonator was maintained at a nominal temperature of 0.01 °C. We must, therefore, correct Cook’s value to the temperature of our experiment. We used the expansion formula recommended by Cook [39]. Uncertainty in this correction has been estimated at 1 ppm [35]. Because our temperatures are measured on the IPTS-68, we use an amended thermal expansion formula [40] to correct our density to other temperatures we have measured. Thus, our mercury density is represented (with an uncertainty of 1.13 ppm near 0 °C) as:

$$\begin{array}{l} \rho_{\text{Hg}}=13.595078_{5}g\cdot {\text{cm}}^{-3}/(1+\alpha_{\text{Hg}}t_{r})\\ \alpha_{\text{Hg}}=181.5253\times 10^{-6}+5.898\times 10^{-9}t_{r}+3.1507\times 10^{-11}t_{r}^{2}+2.405\times 10^{-14}t_{r}^{3}, \end{array}$$

where *t*_r_ is *t*/°C. Cook’s densities are reported for mercury at a pressure of 101.325 kPa, while our volume measurements were made at various pressures within 500 Pa of 101.325 kPa and corrected to that pressure using the value 4×10^−11^ Pa^−1^ [41,42] for the isothermal compressibility of mercury.

The mercury we used was stored in soda-lime glass bottles as described in reference [36] and, we believe, had not been touched since the stock was set aside almost 30 years ago. The only preparation given to the mercury was to filter it through a pinhole in order to remove any surface scum.

A sample of the mercury we used was sent to CSIRO in Australia in order to be compared with a sample of their mercury (which was also standardized by Cook) using the high-precision method of Patterson and Prowse [43]. The comparison showed [44] that the CSIRO sample of mercury was (0.0056±0.0005) kg/m^3^ denser than ours. This compares favorably with the difference, (0.007±0.006) kg/m^3^, measured by Cook. If the densities of these mercury samples did change during the 30 years of storage following Cook’s measurements, the changes were identical (within 0.5 ppm) for the CSIRO sample and our NBS sample.

### 6.4 Weighing

Our guiding principle in design of the weighing aspects of the experiment was that all objects should, as much as possible, have the properties and characteristics of standards used in mass metrology. Volumes of the objects weighed were known well enough so that corrections for air buoyancy could be applied with negligible uncertainty. Temperature and relative humidity were measured within the balance enclosure and atmospheric pressure was measured just outside the balance. The CIPM-81 [45] formula was used to compute the air density.

The balance which we used is described in reference [46]. Its most important features are: 22.7 kg (50 pounds) capacity, single-pan construction (so that substitution, or “Borda” weighing must be used), an enclosure which permits two weights to be interchanged by remote control, and a standard deviation for a single measurement of less than 2 mg.

In practice, we need to know the difference in mass between the weighing bottle filled with mercury and the weighing bottle relieved of about 20 kg. The mass of the filled weighing bottle was balanced against a 50-lb brass standard with a long calibration history. Although its mass does not enter into the final calculation, it is important that the brass weight be stable and that its volume be known to sufficient accuracy [see eq (6.1)].

After partial emptying of its contents into the acoustic resonator, the weighing bottle, along with additional weights, was again balanced against the brass standard. These additional weights were, of course, necessary to make up the deficit in mass of the mercury. Owing to dimensional constraints within the balance, these “counterweights” to the missing mercury had to be specially constructed. The counterweights are crucial to the experiment because it is they which largely determine the mass of the mercury that has been transferred to the resonator. Therefore the counterweights must possess all the properties of a high-quality mass standard and be calibrated in terms of the SI kilogram.

The weighing bottle will now be described, after which we will discuss the counterweights.

#### 6.4.1 Weighing Bottle

**Figure 9:**
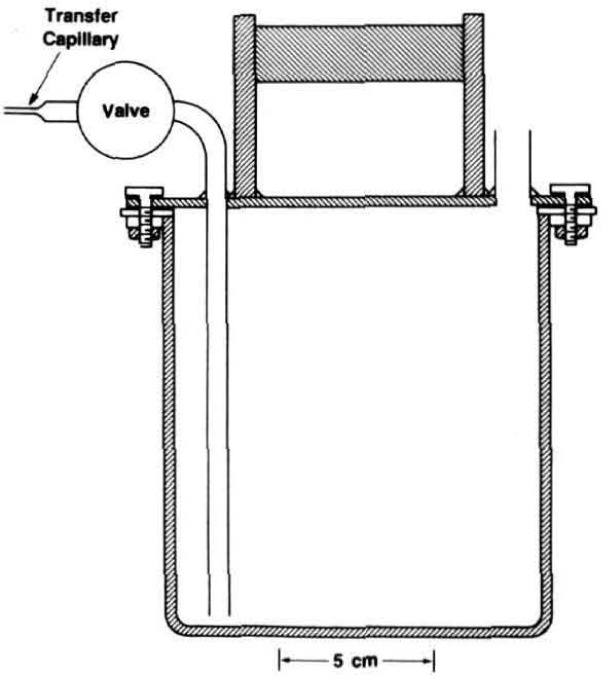
Sketch of weighing bottle.

The weighing bottle sketched in figure 9 was constructed of thin-walled austenitic stainless steel. A 2-L stainless-steel top loading, servo-controlled balance of 15-kg capacity and 0.5 g resolution. The balance was uncalibrated but found to be linear. Each counterweight (distinguished here by the subscript “*i*”) was placed on the balance (in air) and the reading *I*_a_, *_i_* recorded. Next, a plastic bucket filled with distilled water to which had been added 4 parts in 10^5^ (by volume) of non-ionic surfactant was centered on the balance pan. With the bucket in place, the balance was re-zeroed. Then, each counterweight was suspended by monofilament nylon line and lowered into the bucket. The weight, completely submerged, was not allowed to touch the sides or bottom of the bucket. The balance reading *I*_w_, *_i_* was noted, along with the temperature of the water. To the extent that the effects of surface tension on the nylon suspension and that the volume of submerged nylon are negligible, the density of the suspended weight is:

$$\rho_{\text{cw},i}=\frac{I_{a,i}}{I_{w,i}}(\rho_{w}-\rho_{a})+\rho_{a}$$

where *ρ*_w_ is the density of water in the bucket and ρ_a_ is the density of the ambient air. The results of the measurements, at 296 K are:

$$\begin{array}{l} \rho_{\text{cw},1}\;=\left(7.960\pm 0.005\right){\;g/\text{cm}}^{3}\;\left(14.49-\text{kg weight}\right)\\ \rho_{\text{cw},\;2}=\left(7.955\pm 0.012\right){\;g/\text{cm}}^{3}\;\left(5.55-\text{kg weight}\right). \end{array}$$

These results are in agreement with a handbook [47] which reports that both alloys have a density of 8.0 g/cm^3^. The uncertainty estimates were verified by measurements of other objects of known density using the same apparatus.

The masses of the counterweights were determined by direct measurement against NBS mass standards: The smaller counterweight was compared with a 5-kg mass standard plus an assembly of small standards. The larger counterweight plus another assembly of mass standards was compared with a 20-kg mass standard. This time, the 5-kg standard served as the largest weight in the assembly of standards. Finally, both counterweights were compared against the 20-kg mass standard plus an assembly of additional small standards. The three measurements were carried out on two different balances. Final results were arrived at by a linear least squares solution using a weighting scheme which takes into account the differing total variances of each datum [48]. The variances of each measurement were dominated by a combination of the standard deviation of the balance used and the calibration uncertainties of the 20-kg and 5-kg standards.

The mass of the summation of both counterweights is found by the above technique, to be

(20041.1783±0.0019)g(±0.09ppm).

The uncertainty does not include any estimate for the possible deviation of the NBS mass standards which we used from the SI unit as defined by the international prototype kilogram. Work in progress leads us to believe that this deviation could result in a lowering of all reported masses by no more than 0.15 ppm.

#### 6.4.3 Weighing Operations

In this subsection, we describe the sequence of weighing operations necessary to determine the mass of mercury which went into the resonator. The final weighing equation will be given, along with typical values for each term. Finally, we will demonstrate the long-term stability of our weighing procedures.

We began with a full weighing bottle. The filling port of the weighing bottle was capped by a teflon stopper. The weighing bottle was transported from the acoustics laboratory to the mass metrology laboratory indoors on a cart. Upon arrival in the metrology laboratory, the teflon cap was removed and inspected for mercury droplets. By referring to the weighing bottle as “full,” we mean that the level of mercury had been adjusted so that the total apparent mass of weighing bottle plus mercury nearly equaled that of the 50-lb brass weight. Additional, well-calibrated standards were then placed on the weighing bottle to bring its apparent mass to within 50 mg of the 50-lb weight. These additional standards always totalled less than 200 g. Their calibration, even though routine, was known well enough so that negligible error was added to our final result from this source. The 50-lb weight was stored in the balance enclosure throughout the measurements. The full weighing bottle was allowed to equilibrate for at least 3 h inside the balance, although it was kept at room temperature in both the mass metrology laboratory and the acoustics laboratory. During equilibrium, the weighing- bottle port was lightly capped; the cap was removed just prior to the start of weighing.

Weighing was accomplished by single-pan, double-substitution, using a scheme involving five observations [49]. The balance always showed a large drift during the first double-substitution. These measurements were discarded and the weighing continued until the drift rate reached acceptable beaker was modified by the addition of a flange around its rim. A top-plate was bolted onto the flange; the seal between them was made by a teflon gasket. The top plate had a stainless-steel handle welded on, a port for pressurizing or evacuating the interior, and an all stainless-steel bellows-type vacuum valve. A withdrawal tube extended from the valve to the bottom of the beaker. A long, 0.79 mm o.d., stainless-steel, transfer capillary connected to the outlet of the valve was inserted into the resonator when the mercury was transferred. The transfer capillary tube was bent in a loop around the outside of the beaker during weighing operations. As sketched in figure 9, the dimensions of the beaker were chosen so that the height to the flange approximates the diameter.

The volume of the weighing bottle must be known approximately in order to take account of the changes in air buoyancy [eq (6.1)]. For the same reason, the mass of the weighing bottle must be known approximately in order to estimate the mass and the volume of the mercury remaining in the partially emptied weighing bottle. The total mass of the empty weighing bottle was measured to be 1.3987 kg and its volume was calculated to be 191.7 cm^3^. The calculation assumed that the density of the stainless-steel beaker and lid was 7.95 g/cm^3^ and used separate measurements of the mass and volume of the phenolic knob on the vacuum valve. We estimate that the mass is known to 0.1 g and the volume to better than 10 cm^3^.

#### 6.4.2 Counterweights

Two weights were constructed to serve as counters to the 1.5 L of mercury removed from the weighing bottle. The first weight has a nominal mass of 14.49 kg and was initially constructed with the idea that it could be used to counterbalance mercury used to fill a 1-L resonator. This weight is made in the shape of a thick disc with diameter 20.5 cm and height of 6 cm. Two small handles were welded onto the top of the disc. The weight is made of austenitic stainless-steel (type 304) and has been buffed to a lustrous finish.

**Figure 10:**
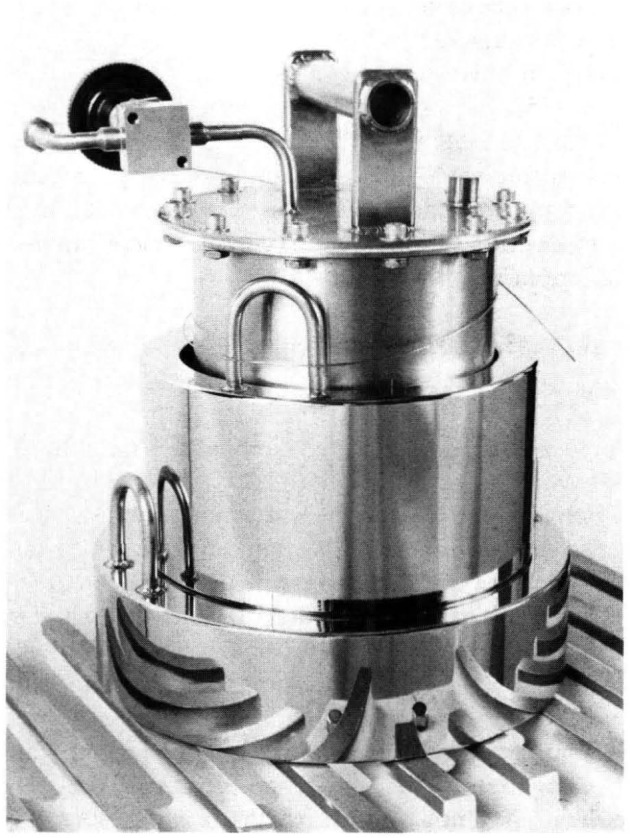
Photograph of weights and weighing bottle ready to be loaded into the balance.

The second weight has a nominal mass of 5.55 kg and is made of similar material (type 316L) with the same surface finish. This weight is tubular in shape, having a height of 9 cm, an inner diameter of 13.5 cm and a wall thickness of 2 cm. The dimensions of the weighing bottle and the two counterweights are such that they can be stacked as shown in figure 10. This assembly can just be accommodated by the balance.

Since the two counterweights are so important to the measurement, considerable care was taken in their calibration. First, their respective volumes were determined. Rather than rely on handbook values for the densities, we measured the density of each weight directly against distilled water. This can be a cumbersome technique for weights of this size. We managed, however, to develop a convenient method which relied on the availability of a levels. The average of three successive double substitutions was taken as the measured difference between the weighing bottle (plus small standards) and the 50-lb brass weight. The temperature inside the balance and the barometric pressure in the room were recorded midway during each double substitution. The relative humidity inside the balance enclosure was measured at the conclusion of the weighings.

Then the port of the weighing bottle was again capped tightly with the teflon stopper. The vessel was removed from the balance and returned to the acoustics laboratory where approximately 1.5 L of mercury was transferred to the resonator in an operation that will be described in detail below. The nearly empty weighing bottle was then capped and returned to the mass metrology laboratory. Here the weighing process was repeated for the weighing bottle and the counterweights. In all, the weighing bottle was weighed on 17 different occasions against the 50-lb brass weight. (Nine times it was full of mercury and eight times it was nearly empty with counterweights added.) Each time, three double substitutions were taken. The pooled standard deviation of one double-substitution was found to be 1.6 mg (34 degrees of freedom) so that the standard deviation of the average of three double substitutions is taken to be 0.9 mg. (The variance of each set of three double-substitutions was consistent with the pooled value, account being taken of the number of degrees of freedom in each variance.)

The mass of mercury actually transferred is given by

$$m_{\text{Hg}}=\frac{m_{\text{cw}}-\rho_{2}V_{\text{cw}}+(\rho_{2}-\rho_{1})(V_{c}-V_{P})+\epsilon_{2}-\epsilon_{1}+\rho_{1}V_{S1-\rho_{2}}V_{S2}+\Delta_{1}-\Delta_{2}}{\left(1-\rho_{1}/\rho_{\text{Hg}}\right)}$$ (6.1)

where

*m*_cw_: = the mass of the counterweights (20041.1783 g)
*V*_cw_: = the volume of the counterweights (2518.3 cm^3^)
*V*_c_: = the volume of the 50-lb, brass standard (2702.9 cm^3^)
*V*_P_: = the volume of the weighing bottle plus remaining mercury (~ 280 cm^3^)
*ϵ*_1_: = mass of small standards added to full weighing bottle (<200 g)
*ϵ*_2_: = mass of small standards added to nearly empty weighing bottle (<200 g)
*V*_S1_: = volume of ϵ_1_ (<25 cm^3^)
*V*_S2_: = volume of ϵ_2_ (<25 cm^3^)
Δ_1_: = balance difference for full weighing bottle (~±20 mg)
Δ_2_: = balance difference for nearly empty weighing bottle (~±20 mg)
*ρ*_Hg_: = density of mercury (~ 13.5378 g/cm^3^)
*ρ*_1_: = density of air during weighing of full weighing bottle (~1.18 mg/cm^3^)
*ρ*_2_: = density of air during weighing of empty weighing bottle (~1.18 mg/cm^3^).

Two such complete weighings were required in filling of the resonator. The total uncertainty in the mass of mercury transferred to the resonator is estimated to be 4.2 mg (0.10 ppm). This follows directly from a propagation of error in eq (6.1). This equation takes air buoyancy into rigorous account but ignores surface differences. For example, changes in relative humidity between runs could affect the mass of the stainless-steel counterweights and this is not accounted for by eq (6.1). We have made reasonable assumptions concerning the possible extent of these effects [50] and find them to be negligible.

In the course of the measurements, we made several observations which give us confidence that the weighings were accomplished to the stated accuracy. Firstly, we had an opportunity to weigh a full weighing bottle twice, 3 days apart. By chance, the ambient air density changed by 2% between the measurements. Such a change is unusually large but should be accounted for if buoyancy corrections have been correctly applied. Indeed, the apparent mass difference between the weighing bottle and the 50-lb brass weight changed by almost 20 mg but this difference was reduced to less than 0.5 mg after the buoyancy correction was applied. On another occasion, there was a 6-month hiatus between successive measurements of the same full weighing bottle. Again, results repeated to within one standard deviation. Finally, on returning a full weighing bottle to the acoustics laboratory, we accidentally allowed a few beads of mercury to escape past the teflon stopper. These were recovered and weighed. The weighing bottle was also reweighed and the results showed that we had recovered the spilled mercury within the precision of the weighings. This observation supports our assumption that close observation of the weighing bottle and its surroundings would have revealed other mercury spills of any significance.

### 6.5 Filling the Resonator with Mercury

In this section we describe how mercury was transferred to the resonator and under what conditions we know the resonator’s volume.

**Figure 11:**
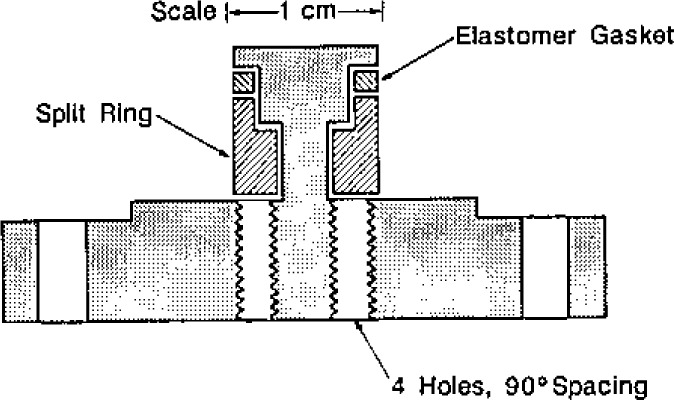
Plug used to seal transducer ports during the measurement of the resonator’s volume.

When ready for filling with mercury, the resonator was configured to look very much like a volume dilatometer. Since the electroacoustic transducers were incompatible with mercury, the ports in the wall of the upper hemisphere were replaced by plugs of special design (fig. 11). Note that the seal was made gas-tight by compression of a 1.6 mm-thick Viton [29] gasket of rectangular cross-section rather than by an O-ring. Our design has advantages over a conventional O-ring seal because the gasket completely fills the annular volume in which it is seated and its compliance does not determine the position of the inner face of the plug with respect to the inner surface of the resonator. This important distance is completely determined by metal to metal contact. A useful design detail is that the screws which compress the gasket were slotted lengthwise to facilitate helium leak testing of the seals.

**Figure 12:**
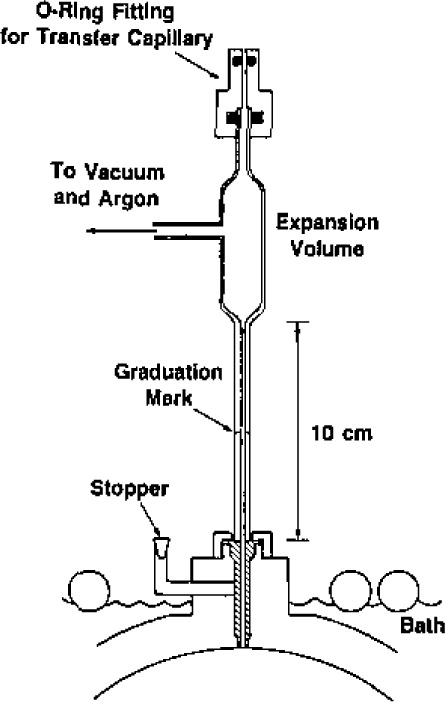
Expansion volume assembly installed on the resonator for the measurement of the resonator’s volume.

**Figure 13:**
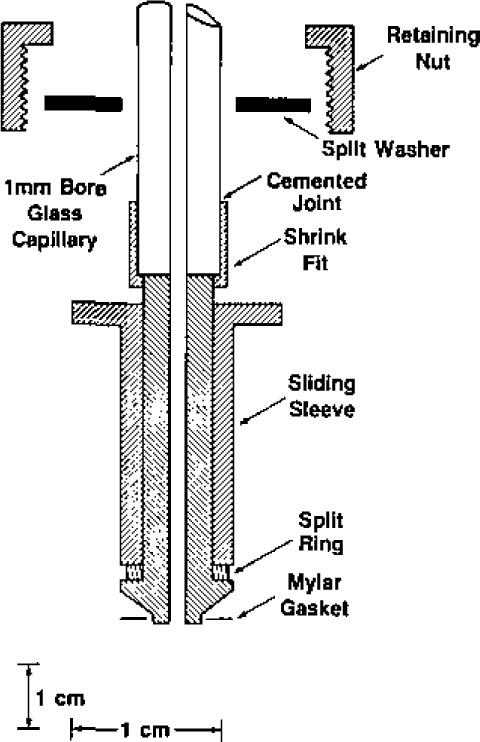
Fitting for sealing the resonator to the glass capillary and expansion volume assembly during transfers of mercury. **Note:** horizontal scale is magnified 2× compared with vertical scale.

As shown in figure 12, the valve at the top of the resonator was replaced with a glass capillary and expansion volume assembly. The assembly was sealed to the resonator by compressing a thin (50 μm) mylar gasket as shown in greater detail in figure 13. The expansion volume was fitted with a side arm, which was used to evacuate the resonator and to back-fill it with argon. A vacuum coupling at the top of the expansion volume assembly was used to seal the stainless-steel transfer capillary which carried mercury from the weighing bottle into the resonator. After the transfer capillary was withdrawn for the weighings, the coupling was sealed with a plug.

The resonator was immersed in a stirred bath of water and methanol whose temperature was controlled to ±1 mK at a nominal 273.16 K. The upper surface of the water was insulated with a layer of hollow polypropylene spheres. The temperature of the resonator at its equator was measured with a calibrated PRT. Thermal gradients about the outside of the resonator were found to be insignificant.

We now describe a typical sequence of operations necessary to fill the resonator with mercury and to determine the volume filled under precisely defined conditions of temperature and pressure.

To begin, plugs were installed in the transducer ports and tested for leaks using a helium detector. The resonator was backfilled with argon and was lowered into the constant-temperature bath. The weighing bottle was placed on a top-loading electronic balance and the stainless-steel transfer capillary was threaded through the expansion volume assembly (fig. 12) into the resonator. The system was evacuated for 15 min in this configuration in order that any mercury in the stainless-steel capillary would be blown into the resonator rather than into the glass expansion volume. The stainless-steel capillary was then withdrawn (sliding through the O-ring seal) as far as the expansion volume so as not to block the glass capillary. The resonator was then evacuated by a diffusion pump fitted with a trap cooled by a slurry of solid CO_2_ and alcohol. Evacuation was slow because of the low conductance of the 1.1 mm i.d. glass capillary tube. The minimum time required for sufficient evacuation was calculated to be 3 hours but we preferred to pump the system overnight.

After evacuation, the stainless-steel capillary was slid down through the glass capillary and into the resonator. The valve on the weighing bottle was opened, and mercury flowed into the resonator under gravity. This 3-hour long process was conveniently monitored by reading the digital balance on which the weighing bottle sat. When the balance indicated that 1.5 L of mercury had been transferred, the transfer was stopped and the half-full resonator was backfilled with argon. The stainless-steel transfer capillary was withdrawn from the expansion volume assembly and the mercury which it contained allowed to drain back into the weighing bottle. The expansion volume was then plugged and the resonator again evacuated and backfilled with clean argon. At this point, the weighing bottle was reweighed, refilled with mercury, and weighed again.

The weighing bottle was then reconnected to the resonator and the transfer of mercury continued in the same way as just described. As mercury finally appeared in the expansion volume, the stainless-steel capillary (with mercury still flowing) was withdrawn to a level just below the mercury surface. This tends to prevent the formation of voids in the glass capillary. The mercury flow was then turned off, the stainless-steel capillary completely withdrawn (as described above), and the volume above the mercury surface again backfilled with clean argon.

At this stage, the mercury level was in the expansion volume. The level was lowered until the meniscus was about midway in the glass capillary by withdrawing mercury using a syringe with special needle. The excess mercury was carefully returned to the weighing bottle. Then, the resonator was again sealed and the volume above the meniscus flushed with clean argon. The now nearlyempty weighing bottle was weighed for the last time, as described above.

Since the resonator was suspended in a water bath rather than in a vat of mercury, the pressures on the inside and outside of the resonator were not equal. However, by adjusting the pressure above the mercury, we could cause the pressures at one horizontal plane to be equal inside and outside the resonator. In order to take advantage of the symmetry of the resonator, we chose the plane of equal pressure to be that of the equator. Then the upper hemisphere was under compression while the lower hemisphere was under tension of equal magnitude. This symmetry suggests that the total volume is identical with the volume of a resonator jacketed by mercury and the suggestion is confirmed by the exact solution of the partial differential equation for the elastostatic equilibrium of a thick shell [51].

Equation (6.2) gives the exterior pressure at the equator and eq (6.3) gives the interior pressure at the equator.

$$p_{\text{ext}}=p_{\text{atm}}+\rho_{w}gd$$ (6.2)

$$p_{\mathrm{int}}=p+\rho_{\text{Hg}}g(h-h_{b}+a)+\frac{2\sigma }{r}|\cos\varphi_{1}|,$$ (6.3)

where

*p*_atm_: = ambient atmospheric pressure
*p*: = argon pressure above mercury meniscus
*d*: = depth of equator below surface of water bath
*ρ*_w_: = density of the bath liquid (water + methanol)
*h* − *h*_b_: = height of mercury meniscus from bottom of fill tube
*a*: = inner radius of shell
*σ*: = surface tension of mercury ≃0.46 N·m^−1^
*r*: = radius of glass capillary (0.557 mm)
φ_1_: = contact angle for mercury on glass (133°)

Notice that in eq (6.3), *h* is itself a function of *p*. In order to solve eqs (6.2) and (6.3) simultaneously, it is necessary to obtain *h* explicitly in terms of *p*. This was done by linear regression analysis of *h* against *p*, where both *h* and *h*_b_ were measured relative to a fiducial mark on the capillary tube using a cathetometer. Given that the result of this fit is

$$h=h_{0}+h_{1}p,$$ (6.4)

the pressure *p* at which the interior and exterior equatorial pressures are equal is:

$$p_{m}=\left(p_{\text{atm}}+\rho_{w}gd-\rho_{\text{Hg}}g(h_{0}-h_{b}+a)-\frac{2\sigma }{r}|\cos\varphi_{1}|\right)/(1+\rho_{\text{Hg}}gh_{1}).$$ (6.5)

In practice, the capillary was long enough to vary the pressure above the mercury from 50 kPa to 250 kPa, and the resulting curve was also extremely useful as a diagnostic tool, as we shall explain below.

### 6.6 Results of Weighing

The volume of mercury which fills the resonator is calculated from the following simple formula:

$$V_{R}=(m_{\text{in}}/\rho_{\text{Hg}})-V_{\text{tube}}(p_{m}).$$

Here *m*_in_ is the net mass of mercury transferred to the resonator from the weighing bottle during the two fillings; *ρ*_Hg_ is the density of mercury at the bath temperature (273.16 K) and at the equatorial pressure; *V*_tube_(*p*_m_) is the volume of mercury in the fill assembly when the pressure above the meniscus is *p*_m_. F_tube_ was determined as a function of *h* in a separate experiment with result:

$$V_{\text{tube}}(h)=-4.7{\text{mm}}^{3}+0.973{\text{mm}}^{2}(h-h_{b}).$$

(This expression is valid only for values of *h* within the glass capillary.) We measured *h* as a function of *p*, as discussed above, and we can use the experimentally determined parameters of eq (6.4) to find *V*_tube_ at *p*_m_:

$$V_{\text{tube}}(p_{m})=[-4.7{\text{mm}}^{3}+0.973{\text{mm}}^{2}(h_{0}-h_{b})]+0.973{\text{mm}}^{2}h_{1}p_{m}.$$

In all, the resonator’s volume was measured three times, with results shown in table 6. Note that measurements two and three were made about 1/2-year apart. The plugs were removed and replaced between volume measurements. We can use the range in the three measurements to estimate a standard deviation of 0.51 ppm for a volume measurement [52], giving an estimated standard deviation of 0.29 ppm for the average. This estimate does not yet include errors which are systematic to the three measurements. It is interesting to note that, although our volume is an order of magnitude larger than that used by Cook [36], our relative precision is not improved over that of Cook.

Besides measuring the mass of mercury necessary to fill the resonator, we also measured the mass of mercury withdrawn from the resonator when it was emptied. This was done for the first two fillings only and is also shown in table 6 as *m*_out_. Note that in both cases, a mass of about 40 mg was either left behind or lost. This corresponds to a fractional volume of 1 ppm, which would be produced by a remnant mercury droplet of radius less than 1 mm. To ensure that any such droplet was ultimately eliminated, the resonator was washed with alcohol after the first and third determinations of the volume and was evacuated at elevated temperature subsequent to the second determination of the volume.

In order to correct each result to a temperature of 273.16 K and an equatorial pressure of 101.325 kPa, it was necessary to know the thermal expansion and the compliance of the resonator’s volume. The experimental determinations of these quantities and measures of their accuracy are described in the next section.

### 6.7 Thermal Expansion

The volume coefficient of expansion of stainless-steel was determined from a handbook [47] value for α_L_, the linear coefficient of expansion in the interval 0–100 °C. The value is:

$$\alpha_{V}\equiv \left(\frac{1}{\Delta T}\frac{\Delta V}{V}\right)=3\alpha_{L}=47.7\times 10^{-6}K^{-1}.$$

We measured α*_V_* directly, because it is conceivable that a structure consisting of two bolted hemispheres with plugged holes might not expand exactly as a homogeneous metal shell. After the second measurement of the resonator’s volume and before the resonator was emptied of mercury, the bath temperature was raised to 302.927 K (the triple point of gallium). With the resonator now behaving like a thermometer bulb, mercury rose in the capillary and filled a portion of the expansion volume. Mercury was then withdrawn by syringe until the meniscus was once again about midway up the glass capillary. The mercury which had been withdrawn was weighed and the volume of the remaining mercury was determined by the methods outlined above. The result of this procedure yielded a value for the thermal expansion of the resonator between 273.16 and 302.93 K:

$$\alpha_{V}=47.59\times 10^{-6}K^{-1}$$

in remarkable agreement with the handbook value.

### 6.8 Compliance

The compliance of the spherical resonator was determined by three methods: 1. theoretically, based on equations for a thick, spherical shell [53] and published values for the elastic properties of 316 stainless-steel [47,54]; 2. experimentally, by measuring acoustic resonance frequency as a function of changes in the external pressure of the resonator; and, 3. experimentally, by measuring the functional dependence of *h* with *p* during volume determinations. The last technique used a published value for the compressibility of mercury which is accurate to about 0.5 percent at the pressure and temperature of interest [41,42].

#### 6.8.1 Theoretical Values of Compliance

The theory of elasticity can be used to calculate the changes in the interior volume of a thick-walled, isotropic, spherical shell in response to pressure changes [53]. The required data are: the inner radius (88.9 mm), the outer radius (108.0 mm), Young’s modulus (197 GPa [47,53]), Poisson’s ratio (0.297 [54]), and the initial and final interior and exterior pressures. We define the compliance of the shell as

$$\chi_{s}=\frac{3\Delta a}{a}\frac{1}{\Delta p},$$

where *a* is the nominal interior radius.

Based on this model, the two experimental determinations of compliance are expected to have significantly different magnitudes. In the first experimental determination, the internal pressure remains fixed while the external pressure is varied. In the second determination, the external pressure remains fixed while the internal pressure varies. The theoretical predictions for these two cases are:

$$\begin{array}{l} \chi_{s,o}=-3.631\times 10^{-11}{\text{Pa}}^{-1}\;(\text{outer pressure varies})\\ \chi_{s,i}=3.012\times 10^{-11}{\text{Pa}}^{-1}\;(\text{inner pressure varies}). \end{array}$$

In addition, we are interested in the case where the inner and outer pressures remain equal:

$$\chi_{s,e}=-6.19\times 10^{-12}{\text{Pa}}^{-1}\;(\text{equal inner and outer pressure})$$

(Note that χ_s, e_ = χ_s, o_ + χ_s, i_.) These calculations are subject to significant errors from the assumed value of elastic constants (about 2%) and, perhaps more seriously, from the assumption that the elastic properties are isotropic throughout the shell. The latter assumption leads to errors in χ_s, e_ which have been “optimistically” estimated at 10% for well-annealed structures of austenitic stainless-steel [55].

#### 6.8.2 Experimental Variation of the Outer Pressure

The compliance upon variation of the outer pressure was measured in a simple auxiliary experiment which exploits the fact that the acoustic resonance frequencies are proportional to the inverse cube root of the resonator’s volume. The resonance frequencies were measured with helium at a pressure of 438 kPa both inside and outside the resonator. With the pressure inside the resonator held constant, the exterior pressure was reduced to 36 kPa and the resonance frequencies were remeasured, Finally the exterior pressure was restored to its original value and the frequencies were measured a third time. The pressure change resulted in fractional changes in the resonance frequencies of four distinct acoustic modes ranging from −5.1 to −5.2 ppm. Upon restoration of the external pressure, the resonance frequencies averaged 0.1_4_ ppm below their initial value. (This very small, apparent hysteresis is most likely a result of incomplete thermal equilibration.) These results can be interpreted as a measurement of the compliance:

$$\chi_{s,o}=(-3.85\pm 0.13)\times 10^{-11}{\text{Pa}}^{-1}.$$

Thus the experimental compliance exceeds the theoretical compliance by about 6%; however, this difference is within the combined errors.

#### 6.8.3 Experimental Variation of the Inner Pressure

The compressibility of the resonator when the inner pressure is varied can be derived from the measurements of *h* as a function of *p*, which have been described above. When fit to a straight line, the slopes, measured for three separate fillings, have a weighted average value of −0.2098 mm/kPa with a pooled standard deviation of 0.0004 mm/kPa. Knowledge of the nominal volume of the resonator and the measured diameter of the glass capillary leads directly to the total compliance,

$$\chi_{T}=(6.93\pm 0.01)\times 10^{-11}{\text{Pa}}^{-1},$$

with a random uncertainty of only 0.2 percent. This total compliance includes the isothermal compressibility of the mercury which is (3.90±0.02) × 10^−11^ Pa^−1^ [41,42]. When it is subtracted from χ_T_, one obtains the compliance of the shell:

$$\chi_{s,i}=(3.03\pm 0.03)\times 10^{-11}{\text{Pa}}^{-1}$$

Remarkably, this result is only 0.6% greater than the calculated value for *χ*_s, i_.

The compliance determined in this experiment is subject to possible systematic errors from two sources. First, a trapped bubble of gas could have led to an erroneous result. Second, mercury may have penetrated unsuspected cracks and pores in the shell and its plugs. Such penetration will be a function of pressure and pore diameter [56] and could be misinterpreted as shell compliance. Because either of these effects might have led to a serious error in the determination of *V*_R_, it is useful to show that they have not occurred.

**Figure 14:**
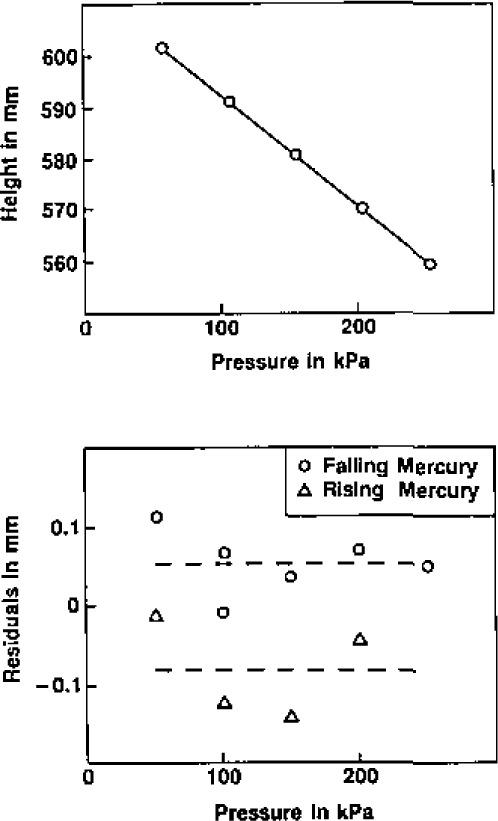
Top: Typical plot of the height of the mercury in the glass capillary as a function of the applied pressure. Bottom: Residuals from a linear fit to the height data.

Our best experimental evidence is based on an analysis of the *h* vs *p* curves. A typical plot is shown in figure 14 (top). Figure 14 (bottom) shows the residuals to the least squares fit to eq (6.4). One is immediately struck by the very small residuals and the clear evidence of hysteresis. All the residuals for points taken with the meniscus falling (caused by raising the pressure) lie above the residuals for the meniscus rising. The average difference between residuals amounts to 0.045 ppm of the resonator’s volume. This very small value of the hysteresis occurred for all three measurements and might be explained as an effect of incomplete thermal equilibration or as an effect of hysteresis in the mercury-glass contact angle, as discussed in the next paragraphs.

In taking data for figure 14, the pressure was incremented or decremented in steps of about 50 kPa. When the pressure is incremented by Δ*p*, the adiabatic temperature increment of the mercury is given by the expression

$$\Delta T=\frac{\Delta p\alpha_{\text{Hg}}T}{\rho_{\text{Hg}}c_{p}},$$

where *c_p_* is the specific heat capacity of mercury at constant pressure [41]. When Δ*p* is 50 kPa, Δ*T* is 1.3 mK. This temperature increment will relax towards the bath’s temperature with a time constant which we estimate using reference [57] to be in the range 270–350 s. (The range results from different approximate treatments of the effect of the shell on the heat flow problem.) As it happened, our experimental protocol called for an equilibration time of 600 s, the time found necessary for the meniscus height to attain equilibrium within 0.1 mm. The 0.14 mm hysteresis on figure 14 is consistent with this protocol and with the condition that, at the time of the height measurement, the average temperature of the mercury had relaxed to 0.13 mK from the bath’s temperature. Approximate solutions to the heat flow problem yield average temperature differences at 600 s in the range 0.08–0.14 mK; thus incomplete thermal relaxation is one mechanism with the correct sign and order of magnitude to explain the observed hysteresis.

Another mechanism which might explain the hysteresis is the documented difference between the “advancing” and “receding” contact angles between mercury and glass. Ellison et al. [58] report that at 25 °C, the maximum advancing contact angle of mercury on polished glass is 147°, the minimum receding contact angle is 122°, and the equilibrium contact angle is 133°. Contact angle hysteresis could appear on the *h* vs *p* curves as a hysteresis in *p* as large as (2*σ*/*r*)×(cos(122°)−cos(147°))=510 Pa, which corresponds to 0.11 mm on Figure 14 (bottom).

#### 6.8.4 Temperature Dependence of the Compliance

As mentioned in section 6.7, the thermal expansion of the resonator was measured between the temperature of the triple point of water and the gallium point. Measurements of *h* vs *p* made at the gallium point were also used to determine the temperature coefficient of χ_T_, the total isothermal compliance of the resonator filled with mercury. The measured value was in satisfactory agreement with our estimate of the sum of mercury and shell contributions. The estimate used the temperature dependence of the compressibility of mercury reported in reference [41] and the temperature dependence of the shell’s compliance calculated from the thermal coefficients of the elastic constants [54].

### 6.9 Tests for Bubbles and Voids

In this determination of *R*, we have assumed that liquid mercury completely filled the resonator during the determinations of its volume. This section describes the measurements and tests which give us a high degree of confidence that this assumption is justified.

The measurements of the meniscus height as a function of the applied pressure can put an upper bound on the total volume of any “large” bubbles that might have been present in the mercury in the resonator. We consider the question: “how large a bubble volume could exist, unseen below the glass capillary, when the pressure above the meniscus was 70 kPa (the approximate gas pressure at which the resonator’s volume was determined)?”.

The compressibility of a bubble can be estimated with the ideal gas law. The correction for surface tension can be neglected for “large” bubbles. The pressure inside a bubble depends upon its vertical position in the resonator; however, this consideration has only a small influence on the model calculations which follow.

To answer the question, we first computed *h* vs *p* curves for various sizes of bubbles in the resonator as the pressure above the meniscus varied from 50–250 kPa. We fit these curves to straight lines and examined the residuals from the fit as in figure 14. A total bubble volume at 70 kPa of 0.1 ppm of the resonator’s volume could not be distinguished from the residuals of figure 14; however, a bubble volume of 1 ppm would lead to residuals which were well off the vertical scale in both directions. We concluded that a total bubble volume of about 0.3 ppm of the resonator’s volume would certainly have been detected.

A similar argument can place upper bounds on the volumes of possible pores, small bubbles, or cracks in the walls of the resonator (such as would have resulted in the unlikely event that the extrusion of the wax from the equatorial seam were incomplete). Mercury intrusion porosimetry is a well-developed analytical technique [56]. At a pressure *p*, mercury will fill a pore of diameter *D* according to the relation

$$D=4\sigma |\cos\varphi_{2}|/p,$$

where *φ*_2_ is the contact angle of mercury on stainless steel [58]. Thus at 50 kPa all pores of diameter 27 μm and larger should already be filled. At the highest pressure, 250 kPa, pores down to a diameter of 5 μm will be filled. The filling of a single pore will be accompanied by a discontinuous jump in the *h* vs *p* curve. The pressure at which the jump occurs determines the diameter of the pore just filled and the magnitude of the discontinuity follows from the pore volume.

From the above single pore model and our compressibility data we concluded that the pore volume entered by the mercury between pressures of 50 kPa to 250 kPa was less than 0.1 ppm of the resonator’s volume. At the other extreme from a model of discrete pores, we can imagine a continuous distribution of pore diameters and volumes which would change the slope of the *h* vs *p* line instead of producing step-wise discontinuities. The agreement between the measured value of χ_s, i_. with that calculated from the theory of thick shells would not have been possible, however, if the volume contained in such a continuous distribution were greater than 0.1 ppm of the resonator’s volume.

Finally, we recall that in section 6.8.4 we reported that the measured temperature dependence of χ_T_ was in satisfactory agreement with the value calculated from published data for mercury and 316 stainless steel. The presence of a bubble of sufficient size would have destroyed this agreement. Based on the uncertainties involved in checking the temperature dependence of the compressibility, we put the size of a possible bubble at less than 0.3 ppm of the resonator’s volume. This limit is the same as was found in the analysis of the compressibility data at 273.16 K.

### 6.10 Corrections from Weighing Configuration to Acoustics Configurations

The small difference in volume between the calibration and operational configurations of the resonator was determined in auxiliary experiments and calculations. The difference results from three changes: 1. the expansion volume assembly is replaced with a valve, 2. the plugs are replaced with the electroacoustic transducers, and 3. the resonator is supported from the valve at the north pole instead of three bolts tangential to the equator.

The volume displaced by the valve stem (in the closed position) was compared with that of the filling-tube adaptor by simple mechanical measurements, These were performed using a dummy valve body fabricated to the same internal dimensions as the filling port of the resonator. The surface corresponding to the inner wall of the resonator was machined flat and served as a reference plane for the measurements. Using a calibrated dial gage supported in the chuck of a milling machine, the expansion volume assembly was found to extend forward of the reference plane by (0.20±0.01_3_) mm. This measurement was performed with a mylar gasket in place and with the retaining nut tightened so as to mimic the expansion volume assembly used during calibration of the resonator. Similarly, the firmly closed valve stem was measured to be (0.31 ± 0.01_3_) mm behind the reference plane and we therefore add (4.0±0.2) mm^3^ to the volume of the resonator to account for this difference.

**Figure 15:**
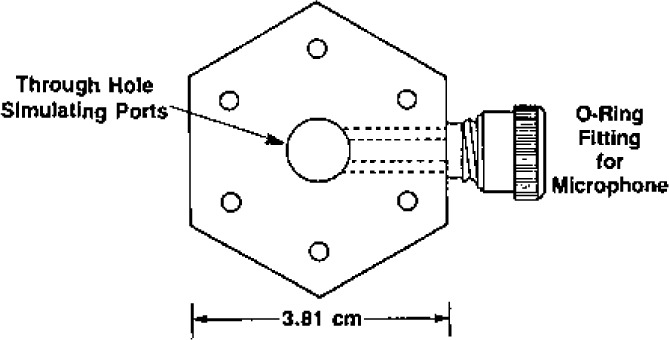
Acoustic coupler used to measure the difference between the volumes displaced by the electroacoustic transducers and the volumes displaced by the plugs which replaced the transducers during volume measurements.

The volume displaced by the transducer assemblies was compared with that of the stainless-steel plugs by measurements of acoustic pressure with a small sealed coupler. This scheme was adopted because the tensioned diaphragms on the transducers were too delicate to permit dimensional measurements using mechanical contacts. As shown in figure 15, the coupler consisted of a brass bar bored through to the diameter of the transducer ports. Each end of the coupler was provided with bolt holes so that either a plug or a transducer assembly could be installed. With both ends closed in this manner, the free volume within the coupler was approximately 370 mm^3^. A second hole bored through the side of the coupler and fitted with an O-ring seal accommodated a microphone (B&K type 4138) [29]. The microphone was used in conjunction with a preamplifier (B&K type 2660), a lock-in amplifier, and a digital voltmeter to measure the acoustic pressure. One end of the coupler was closed by a transducer assembly which was driven as a frequency-doubling source by a constant excitation signal at 500 Hz. The other end was closed either by the second (passive) transducer assembly or by one of the two plugs. The acoustic pressure was measured (with ambient air inside the coupler) in each of the three possible configurations. The plugs and transducers were sealed in the apparatus in exactly the same way as they would be in the spherical resonator. The roles of the active and passive transducers were then reversed in order that the displacement of the second transducer could be compared with that of the plugs. In each case, the free volume *V*_i_ was taken to be inversely proportional to the measured acoustic pressure *p*_i_ so that a comparison of two configurations i and j gives

$$\begin{array}{l} \left(V_{i}/V_{j})=(p_{j}/p_{i}\right)\\ V_{i}-V_{j}=V_{j}(p_{j}-p_{i})/p_{i}\approx 370{\text{mm}}^{3}(p_{j}-p_{i})/p_{i}, \end{array}$$ (6.6)

where the approximation is valid for small differences in volume. The results are summarized in table 7 and lead us to increase the measured volume by (12.4±0.1) mm^3^, where the small uncertainty is based on the close agreement between the different permutations of plugs and transducers; it does not include any assessment of systematic uncertainty.

The only significant systematic uncertainty results from the effects of the “annular” gaps which occur between the wall of the resonator or the coupler and the plugs or transducer housings. (Note: there was no centering mechanism to guarantee that the gaps were actually annular.) Beads of mercury were always found in the gaps when the plugs were removed from the resonator’s ports between volume measurements, leading us to assume that mercury filled the gaps during volume measurements. The volume in the gaps was found to be (1.40±0.10) mm^3^ from dimensional metrology. (The diameters of the plugs were 9.51_2_ and 9.50_0_ mm; the distances between their ends and the gaskets were 1.78 and 1.73 mm. The diameters of the ports were 9.54_5_ mm and 9.52_0_ mm.)

The geometric volumes of the gaps between the plugs and the hole through coupler adds up to 1.0 mm^3^. (The i.d. of the coupler is 9.52_5_ mm.) The geometric volumes of the gaps between the transducer housings and the hole through the coupler add up to 5.0 mm^3^. (One transducer housing extends into the coupler 8.68_2_ mm and is 9.50_2_ mm in diameter; the other extends 8.72_0_ mm and is 9.51_2_ mm in diameter.) We now consider the question: what is the effective volume of these gaps as measured by the acoustic field when the plugs or the transducers were in the coupler? In all these cases, the widths of the gaps were much less than the viscous and thermal penetration lengths (70 μm and 81 μm, respectively) for motions of air at the measuring frequency (1 kHz). Under such conditions, sound propagation in the gaps is essentially isothermal and the effective volume of the gaps can exceed the geometric volume by a factor as large as *γ* (*γ* ≈ 1.4 for ambient air), if attenuation in the gaps is neglected. If attenuation in the gap is considered, the effective volume can be much smaller than the geometric volume. The effective volume can be calculated for a long slot of depth *D* with plane walls separated by a distance *d*. The result is [27]:

$$V_{\text{eff}}=(-i\beta /kD)V_{\text{geometric}},$$

where *k* = 2*πf/c*_air_ is the propagation constant for sound in air and *β* is the specific acoustic admittance given by

$$\begin{array}{l} \beta \approx \{(1+i){\left(3\gamma \right)}^{1/2}/6(\delta_{s}/d)\}\\ \times \tanh\{(1+i){\left(3\gamma \right)}^{1/2}(\delta_{s}/d)kD\}. \end{array}$$ (6.7)

For each of the gaps under discussion, *V*_eff_ is a comparatively small complex quantity whose real part must be added to the much larger “free volumes,” (*V*_i_ and *V*_j_) in eq (6.6) to obtain the volume difference from the measurements of the amplitudes of acoustic pressures [*p*_i_ and *p*_j_ in eq (6.6)]. For each gap, *V*_eff_ was calculated twice using eq (6.7) with different assumptions representing two extreme cases. First, we assumed that the gap was exactly annular and that its width, *d*, was half the difference between the diameter of the hole in the coupler and that of the inserted object. Second, we assumed that the inserted object was in contact with the wall of the coupler somewhere on the circumference. This led to a gap whose width varied as *d*·sin^2^(*θ*) where *θ* measures the angle from the point of contact. In this case a numerical integration of eq (6.7) was necessary. Under these different assumptions, the real parts of the effective volumes for the plugs in the coupler totalled 1.3_2_ mm^3^ and 1.3_1_ mm^3^, respectively. For the transducer housings in the coupler, the effective volumes totalled 1.5_3_ mm^3^ and 2.7_6_ mm^3^. We shall take the average of the effective volumes computed under the two extreme assumptions as the best estimate of their values. For purposes of propagation of errors, we shall take 1/3 of their difference as an estimate of the standard error in the calculations. Another contribution to the error in these calculations results from the imperfect measurements of the average width of each gap (±0.0018 mm) and is added in quadrature.

Several other phenomena enter into the comparison of the volumes displaced by the plugs to the volumes displaced by the transducers; however these had negligible effects on the results. For example, the volumes were compared at room temperature; however their difference must be known at *T*_t_. The plugs were made of type 304 stainless-steel and the transducer housings were made of brass; however, the effect of the difference in their thermal expansions is very small (≈0.02 ppm of the resonator’s volume), A portion of the volume displaced by the transducers is bounded by a compliant diaphragm. The compliance increases the apparent volume displaced in the coupler by a very small amount which is proportional to (pressure/frequency) at frequencies well below the diaphragm’s resonance (≈ 100 kHz). From information provided by the manufacturer, the equivalent extra volume is on the order of 0.15 mm^3^ (0.05 ppm) for each transducer under the conditions of the comparison.

While the resonator’s volume was measured, the resonator hung in the water bath from three vertical bolts which were screwed into holes in the cylindrical portion of the resonator near the equator. While the acoustic measurements were carried out, the resonator was hung from the valve body attached to the north pole. In this latter configuration, the sagging of the resonator under its own weight increased its volume by 0.04 ppm over that determined in the weighing configuration. This increase was estimated from an exact solution to the partial differential equation for elastostatic equilibrium for a thick, homogeneous, spherical shell [51].

We shall now summarize the results of this section. The volume of the resonator determined by weighing the mercury required to fill it must be corrected in the following six ways. 1. A volume of (4.0±0.2) mm^3^ must be added to account for the replacement of the expansion volume assembly by the valve atop the resonator. 2. A volume of (1.4±0.1) mm^3^ must be subtracted to account for the mercury intrusion in the gaps between the plugs and the ports on the resonator. 3. The volume of (12.4±0.1) mm^3^ which was determined by acoustic measurements with the coupler must be added to account for the replacement of the plugs by the transducer assemblies. 4. The calculated volume (2.15±0.41) mm^3^ must be subtracted to account for the acoustic effects of the gaps between the transducers and the coupler. 5. The calculated volume (1.31 ±0.13) mm^3^ must be added to account for the acoustic effects of the gaps between the plugs and the coupler. 6. The calculated volume 0.04 ppm must be added to account for the sagging of the resonator under its own weight. The net correction is (14.2±0.5) mm^3^ out of 2943 cm^3^.

## 7. Determination of *M/γ*_0_

Our determination of *M/γ*_0_ is ultimately based on a sample of nearly monoisotopic Ar^40^ especially manufactured and analyzed for the present determination of *R*. We removed the chemically reactive impurities from this sample and measured the relative abundances of the remaining noble gas impurities sufficiently well to establish *M* for this sample within 0.7 ppm. As discussed below, we assumed *γ*_0_≡5/3 for this sample. A series of acoustic resonance frequency measurements was used to compare the standard sample to the working sample of argon (which was actually used for the determination of *R*). This series was optimized to determine the ratio of the speeds of sound between the two gases and it established *M/γ*_0_ of the working sample with a total inaccuracy of 0.8 ppm, as indicated in table 8.

We have independently estimated *M/γ*_0_ for the working sample of argon by assuming that it had the same relative isotopic abundance ratios as those measured by Nier in commercially supplied argon. This estimate, also included in table 8, has a precision of 2.0 ppm and is in agreement with the value of *M/γ*_0_ we obtained using the nearly monoisotopic Ar^40^. After we made this estimate, we learned that Cohen and Taylor [1] also reevaluated Nier’s data. Their value for *M/γ*_0_ and its error are the last entries in table 8. Cohen and Taylor’s conclusions are consistent with our own.

We had intended to redetermine *R* using both argon and helium samples; however, progressive contamination of the helium within the resonator prevented us from making reliable measurements. Common impurities (such as air and water) change the speed of sound in helium 10–30 times more than they change the speed of sound in argon. Thus the contamination that interfered with the helium measurements did not affect the argon measurements.

In this section, we shall describe the sources and analysis of the gases we used, the effects of impurities, the determination of the speed of sound ratios used to determine *M/γ*_0_ of the working gas, and finally our independent estimate of *M/γ*_0_ using published values of isotopic abundance ratios of argon.

### 7.1 Chemical Composition of the Gases Used

Information concerning the chemical composition of the gases we used was provided by the manufacturers and will be listed here. This information was supplemented by our analysis of certain gas samples subsequent to their use, particularly for trace amounts of other noble gases.

We have used argon samples from three different sources and helium samples from two different sources. The standard argon sample, denoted Ar-40, was purchased from the Mound Facility of the U.S. Department of Energy, Miamisburg, Ohio, [29] in 1984. This sample of nearly monoisotopic Ar^40^ was prepared for these measurements of *R*. The supplier provided a detailed report based on mass spectroscopy which showed the following abundances: Ar^36^<3 ppm; Ar^38^, 30.8±3 ppm; N_2_, 12±5 ppm; O_2_, 5±2 ppm; CO_2_, 4± 1.2 ppm; Kr<6 ppm; and Xe<7 ppm. Here, the error and detection limits are two standard deviations, based on four replicate analyses. As discussed in section 7.2, our measurements of the speed of sound in portions of this sample provided evidence that in May 1987 the gas in the supplier’s container included approximately 35 ppm of CO_2_, in serious disagreement with the supplier’s analysis. In July 1987, the supplier re-analyzed the remaining gas. The analysis showed that the relative abundances of Ar^36^ and Ar^38^ were unchanged and that CO_2_ and mass 28 (CO or N_2_) impurities were present. Such chemically reactive impurities were removed from this gas prior to its use as a standard by the procedures described below. Thus their presence in the supplier’s container did not degrade the accuracy of the redetermination of *R*.

The working argon sample, denoted Ar-M, was purchased for Matheson Gas Products [29] in 1984. From a lot analysis (lot E30 000 6D8, cylinder 45024T), the supplier provided the following upper bounds for impurities: N_2_<3 ppm; O_2_<1 ppm; H_2_O< 1 ppm; and total hydrocarbons <0.5 ppm. A third argon sample, denoted Ar-A, was purchased from Airco Inc. [29] in 1986. The supplier provided an analysis (test number AN28, cylinder number CC-58939) which stated: total impurities < 1 ppm, including traces of O_2_ and N_2_.

One helium sample, denoted He-M, was purchased from Matheson Gas Products [29] in 1985 (Lot No. G55-0158-B1). From a lot analysis, the manufacturer stated that the minimum purity was 99.9999 mole percent. Upper bounds were provided for certain impurities: O_2_<0.1 ppm; N_2_<0.4 ppm; Ar<0.1 ppm; CH_4_<0.1 ppm; CO <0.1 ppm; CO_2_<0.1 ppm; and H_2_O<1 ppm. The second helium sample, denoted He-BM was obtained from the Bureau of Mines Helium Research Center in about 1970 (Cylinder No. 139177) and has an unbroken chain of custody. Portions of this gas had been used to calibrate Burnett *PVT* apparatus. The Bureau of Mines provided the remarkable analysis: H_2_, 0.16 ppm; CH_4_<0.005 ppm; H_2_O, 0.5 ppm; Ne, 0.40 ppm; N_2_, 0.08 ppm; O_2_, 0.01 ppm; Ar< 0.005 ppm; CO_2_, 0.03 ppm.

Gas chromatographic analyses of three gas samples were conducted with the help of Mark Sirinides of the Quality Control Laboratory of Airco Industrial Gases, Riverton, N.J. [29]. Samples of Ar-40 and Ar-M which had been used for acoustic measurements were condensed out of the resonator into cylinders prepared for their storage. Portions of these “used” gases were analyzed in November 1986 with the following results. For Ar-40 we found: Ne, 0.9 ±0.3 ppm; Kr, 2.2 ±0.3 ppm; Xe, 1.3±0.3 ppm and N_2_, <4.5 ppm. The imprecision in the measurement of the xenon abundance is the largest source of the imprecision in the determination of *M/γ*_0_ for this standard sample. The calibration of the chromatograph for the important xenon analysis was based on two standard mixtures, one prepared by Mr. Sirinides, and a second prepared by us, independently. The comparatively coarse upper bound on any possible N_2_ impurity was a consequence of our inability to thoroughly purge the inlet to the chromatograph with the small sample of “used” Ar-40. The chromatographic analysis of Ar-M detected no impurities and established the bounds: Ne<0.3 ppm; Kr<0.3 ppm; Xe<0.3 ppm; and N_2_<2.5 ppm. The chromatographic analysis of He-M detected no impurities. Two bounds were established for He-M: Ne<0.3 ppm and N_2_<1.8 ppm.

In September 1987, the remaining Ar-40 in its original container was shipped to the Helium Field Operations Facility of the U.S. Bureau of Mines in Amarillo, Texas to be analyzed for possible helium content. This was accomplished with a special mass spectrometer which had been built to redetermine the relative abundance of helium in the atmosphere. [59] The analysis yielded the result (1.5 ±1.0) parts per billion ^4^He in the argon.

### 7.2 Effects of Impurities and Purification

#### 7.2.1 Effects of Impurities

For low densities, the speed of sound in a gas mixture can be estimated from the relation $c_{0}^{2}=C_{p}^{0}RT/(C_{V}^{0}M)$. For mixtures, $C_{p}^{0}$, $C_{V}^{0}$, and *M* are mole fraction averages of $C_{p}^{0}$, $C_{V}^{0}$, and *M* of the components. We have used these relations to calculate the derivative of $c_{0}^{2}$ with respect to mole fraction of various possible chemical impurities. The results for helium and argon, along with the values of *γ*_0_ which were used to calculate $C_{p}^{0}$ and $C_{V}^{0}$, are listed in table 9. From table 9 one can conclude, for example, that 1 ppm of water in helium will decrease $c_{0}^{2}$ by 3.93 ppm and that 1 ppm of water in argon will increase $c_{0}^{2}$ by 0.12 ppm.

When the resonator was filled with helium, we measured a slow decrease in the resonance frequencies. In a typical case the decrease was 9.3 ppm/100 h with 438 kPa of He-M in the resonator. In contrast, when the resonator was Filled with argon, we never observed a secular change in the frequencies. (In one case, a change of ±0.5 ppm/100 h would have been detected with Ar-M in the resonator at 100 kPa.) We speculate that slow desorption of impurities is responsible for these effects. Possible sources of water etc. are the “Viton” [29] O-rings which seal the microphone ports and the fill port of the resonator. From table 9 it is clear that the speed of sound in helium is much more sensitive to most impurities than the speed of sound in argon. For common impurities, levels below 1 ppm would be disastrous in helium yet they would be barely detectable by speed-of-sound measurements in argon.

One might imagine that a tiny droplet of mercury remained in the resonator after the volume determinations. Such a droplet would have a vapor pressure of 25 mPa near *T*_t_. If mercury were present at this partial pressure in argon under typical measurement conditions (300 kPa) and if it were not accounted for, the resulting value of *R* would be 0.3 ppm too small. In practice, the speed of sound was fitted to a function of pressure which included a *p*^−1^ term, primarily to account for the effects of the thermal accommodation length. This term would account for the effect of saturated mercury vapor, if vapor were present.

#### 7.2.2 Apparatus and Procedures for Purification

**Figure 16:**
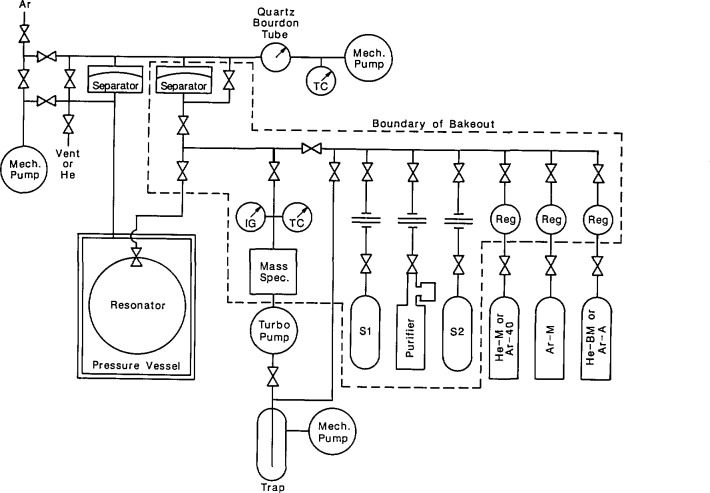
Schematic diagram of gas handling system.

The manifold used for loading the resonator, measuring the pressure, purifying the Ar-40, and collecting samples of gas for analysis, is diagrammed in figure 16. The portion of the manifold which is enclosed by the “Boundary of Bakeouf” on figure 16 was made entirely of metal and was baked at temperatures above 100 °C prior to use. The valves used metal bellows for stem seals and the demountable joints were sealed with nickel gaskets. Except for very short transition pieces, the tubing had a diameter of at least 0.95 cm (3/8 inch).

A purification procedure was followed with the Ar-40 used to standardize the Ar-M. The gas to be purified was admitted to a stainless-steel cylinder (10 cm i.d. and 55 cm high) hung from the manifold on a 4 cm i.d. tee. (See fig. 16.) The temperature of one side of the cylinder was increased to 80 °C using a heating tape, in order to force circulation of the gas within it. The side-arm of the tee atop the cylinder opened to a zirconium-aluminum alloy getter (Model GP50, S.A.E.S. Getters Inc. [29]) which was maintained at a temperature near 400 °C. Data provided by the manufacturer of the getter indicated that it is very effective in removing active gases (such as CO, CO_2_, O_2_, N_2_, and H_2_) from the noble gases and it is moderately effective in removing hydrocarbons.

In an early test of the purification procedure, about half the impurity (probably CO_2_) was removed from an Ar-40 sample at 500 kPa in 28 h. In subsequent use, the getter was maintained at a higher temperature, the convective mixing was improved, and the purification was conducted at pressures of 280 kPa or lower. With these changes, more than 90% of the impurities in an Ar-40 sample were removed in 26 h. This can be seen from table 10: in 26 h the ratio *c*(Ar-40)/*c*(Ar-M) changed 90% of the way from its value for unprocessed Ar-40 towards its value for Ar-40 purified for 120 h, or more.

The purification procedure we have described did not change the speed of sound in two Ar-M samples by more than 0.3 ppm.

The speed of sound in the unprocessed Ar-40 had a measurable dispersion which was eliminated by the purification. Purification for 120 hours increased the speed of sound in Ar-40, as determined from the (0,2)–(0,6) modes at 100 kPa by 9.2, 8.0, 7.5, 7.0, and 6.8 ppm, respectively. Purification also reduced the excess half-widths of the same modes by 2.4, 2.3, 2.0, 1.7, and 1.5 ppm of their respective frequencies.

The dispersion and the excess half-widths in the speed-of-sound data for the unprocessed Ar-40 can be quantitatively interpreted as effects of a relaxing impurity such as CO_2_. Simpson et al. [60] determined the relaxation time τ_m_ of a (0.1CO_2_+0.9Ar) mixture. Their results can be applied to other CO_2_ +Ar mixtures using the mole fraction dependence established by Kneser and Roesler [61]:

$$\frac{1}{\tau_{m}}=\frac{1-x}{\tau_{\text{Ar}}}+\frac{x}{\tau_{{\text{CO}}_{2}}},$$

with the parameters $\tau_{{\text{CO}}_{2}}=(8\pm 1)$ μs and τ_Ar_=(50±10) μs at 273 K and 100 kPa, and where *x* is the mole fraction of CO_2_. Consequently, the CO_2_ impurity in argon would increase the half-widths by δ*g* where

$$\frac{\delta g}{f}=\frac{2x}{15}\frac{\omega \tau }{1+{\left(\omega \tau \right)}^{2}}.$$

This formula fits the decreases in the half-widths produced by the purification of Ar-40 with the parameters τ_Ar_=46 μs and *x* = 35×10^−6^. These parameters can be used to predict changes in the resonance frequencies with the relation

$$\frac{\delta f}{f}=-\frac{2x}{15}\left[1+\frac{1}{1+{\left(\omega \tau \right)}^{2}}\right]-\frac{x}{20}.$$

The predicted frequency changes are 9.5, 8.3, 7.6, 7.2, and 7.0 ppm for the (0,2)–(0,6) modes. All are within 0.3 ppm of the measured changes. This agreement and the agreement of the measured relaxation time with the literature value for CO_2_ strongly suggested that the unprocessed Ar-40 contained much more CO_2_ than indicated in the manufacturer’s original analysis.

The study of the purification process and the determination of speed-of-sound ratios discussed in the next section were completed in May 1987. Then, the remaining Ar-40 was returned to the manufacturer in its original stainless-steel container. The manufacturer re-analyzed the gas in July 1987 via mass spectroscopy and reported the following abundances: Ar^36^, <3 ppm; Ar^38^, 35 ppm; CO_2_, 32±5 ppm; O_2_, 7±2 ppm; N_2_ + CO, 37±15 ppm. As one might expect, the relative abundances of the argon isotopes did not change. The lack of change in O_2_ demonstrates that air leakage was not a factor. The manufacturer speculated that CO_2_ and CO were being formed in the container by a reaction involving the metal oxide, adsorbed hydrocarbon films, and possibly adsorbed water. In any event, the presence of chemically reactive impurities in the supplier’s container does not degrade the accuracy of the re-determination of *R* because these impurities were removed prior to the determination of the ratios.

### 7.3 Determination of Speed of Sound Ratios

The last part of the laboratory work in the present determination of *R* was a series of measurements to determine the ratios of the speeds of sound in Ar-40 and Ar-A samples to the speed of sound in the working gas, Ar-M. The ratio determinations had an imprecision of only 0.2 ppm in c and were used to determine *M/γ*_0_ for the Ar-A and Ar-M samples from the value of *M/γ*_0_ computed for Ar-40 sample.

The speed of sound ratios were obtained from measurements of the acoustic resonance frequencies at temperatures near *T*_t_ when the resonator was sucessively filled with an Ar-M sample and another gas sample. The successive fillings were at very nearly the same pressure and temperature. Each pair of fillings and its associated resonance measurements were completed within 4–12 h. These precautions minimized the effects of drifts in the pressure transducers and in the resistance bridge used for temperature measurement. To minimize the effects of imperfections in the resonator’s geometry and imperfections in our model of its elastic properties, the speed of sound ratios were obtained from a mode by mode comparison of the resonance frequencies, after application of the usual corrections for the thermal boundary layer, the accommodation length, and the resonator’s compliance. (The mass dependence of the transport properties and virial coefficients can be neglected.) Typically these ratios determined from the (0,2)–(0,6) modes had an rms deviation from their mean of 0.1 ppm; thus, the 0.2 ppm error in the ratio determination results from another source, probably the thermometry. The results of the ratio measurements are listed in table 10. The frequencies that were used in the important comparison of Ar-40 to Ar-M are tabulated in Appendix 3. For these ratio determinations only, the frequencies were determined from 6-parameter fits of eq (4.1) to the voltage vs frequency data. Thus, we exploited the fact that 6-parameter fits yielded more precise frequency measurements than 8-parameter fits. Although the 6-parameter fits are influenced by the systematic effects of mode overlap, the mode by mode comparison of frequencies accounts for these effects.

### 7.4 Determinations of *M/γ*_0_ for the Working Gas

After purification, the Ar-40 sample was a mixture of argon isotopes and other monatomic gases. Thus, neither rotational nor vibrational degrees of freedom need be considered in the calculation of *γ*_0_. The lowest electronic state of argon is 11.7 eV above the ground state. It follows that at *T*_t_ and the densities of interest here, electronic contributions to *γ*_0_ are negligible. Thus we take *γ*_0_=5/3 for Ar-40. The mole fraction averaged atomic mass is calculated using the isotopic abundances provided by the manufacturer (Ar^36^=0±3 ppm; Ar^38^=31±3 ppm), the noble gas abundances measured by chromatography, (Ne=0.9±0.3 ppm; Kr=2.2±0.3 ppm; Xe=1.3±0.3 ppm) and the atomic masses from the 1983 atomic mass evaluation [62]. For the present purpose the most important value is 39.9623837 g/mol (±0.035 ppm) for Ar^40^ on the carbon-12 scale. The resulting value of *M/γ*_0_ for the purified Ar-40 sample is 23.97751_0_ g/mol with an uncertainty of ±0.7 ppm originating from the uncertainty in the chromatographic measurement of the xenon in the Ar-40.

To calculate *M/γ*_0_ for Ar-M from the value for the Ar-40 sample, we assumed the *M/γ*_0_ varies inversely as the square of the measured speed-of-sound ratio. The measurements were at nonzero pressure; thus we neglected the very small differences in the virial coefficients of the different argon samples. The mean value of the square of the ratio for the Ar-40 samples purified longer than 26 h (table 10) has an rms deviation of 0.4 ppm. This value leads to the *M/γ*_0_=23.96868_1_ g/mol for the working gas with a combined uncertainty of 0.8 ppm.

### 7.5 Estimating *M/γ*_0_ from Isotopic Abundance Ratios

For the Ar-M and Ar-A samples prepared commercially from liquid air, we can estimate *M/γ*_0_ using Nier’s measurements [63] of the relative abundances of the isotopes in argon from liquid air. Nier found slightly less Ar^36^ and Ar^38^ in argon purchased from a commercial supplier than in argon which he obtained by passing air over hot lithium metal. Nier’s “weighted grand mean” data for commercial argon yields abundance ratios: Ar^36^/Ar^40^=(3346±6)×10^−6^ and Ar^38^/Ar^40^= (630±1) × 10^−6^. (Here, the errors are Nier’s “probable errors” which we take to mean 0.674 standard deviations.) These abundances can be combined with the 1983 atomic masses to obtain an average molecular mass for Nier’s commercial argon of 39.947815 g/mol.

We must now address two issues. One is the accuracy to be assigned to Nier’s data, and the second is the likelihood that the Ar-M and Ar-A samples had the same relative abundances as Nier’s argon. Nier’s “probable error” of his “weighted grand mean” propagates into a standard deviation of 0.96 ppm in *M*, the average molecular mass. Nier used two different mass spectrometers which were calibrated with different standards which he made from isotopically separated samples. Nier’s table II and table III provide sufficient data that one can recalculate both the “grand mean” and its standard deviation. We have done so in a manner which makes a more conservative allowance for the few degrees of freedom present. (There are only three measurements of the important abundance ratio Ar^36^/Ar^40^ with each spectrometer.) On the basis of this recalculation, the most probable value of *M* is 39.94779 g/mol, 0.6 ppm smaller than Nier’s value. On the basis of our calculation, we have enlarged the estimate of the error in *M* to 2.0 ppm.

We now consider whether or not the Ar-M sample purchased in 1984 and the Ar-A sample purchased in 1986 are equivalent to Nier’s argon which was commercially supplied 35 years earlier. Evidence concerning the equivalence of “recent” argon and Nier’s argon is provided by abundance ratio measurements made in connection with Quinn, Colclough, and Chandler’s [64] 1976 remeasurement of *R*. The ratio of the abundance of Ar^36^ to the abundance of Ar^40^ was measured for two commercially supplied samples and one sample obtained by chemical removal of the reactive components of air. The abundance ratio was 0.4% and 0.6% smaller for the commercially supplied samples than the ratio for chemically purified argon. (The same abundance ratio for Nier’s commercially supplied argon was 1% smaller than the ratio for Nier’s chemically purified argon.) We conclude first, that the commercial process for extracting argon from liquid air does indeed change isotopic abundances. (Nier also measured comparable changes in relative isotopic abundances for other gases produced from liquid air.) We also conclude that the 0.5% difference between the two measurements of the abundance ratios is an independent assessment of certain possible errors in Nier’s mass spectroscopy. This difference is equivalent to 1.5 ppm in *M*. Finally, we note that the (0.2_8_±0.1_3_) ppm difference between speed of sound in Ar-A and Ar-M is equivalent to (0.5_6_±0.2_6_) ppm in *M*. Such a small difference could not have been resolved by Nier’s measurements.

In this assessment of *M* for commercially supplied argon, we have not used the more recent data of Melton et al. [65]. Melton et al. did not use synthesized standards to calibrate their mass spectrometer and they relied, in part, upon Nier’s data as evidence that fractionation did not occur within their mass spectrometer’s inlet system.

We note that Quinn et al. [64] have interpreted the published data concerning the relative abundances of the isotopes of argon in a quite different way. In particular, they have chosen to rely on the abundance data obtained by Melton et al. [65] with commercially supplied argon and they have accepted the conclusion of Melton et al. that the isotopic compositions of commercially supplied and atmospheric argon are the same. Thus, Quinn et al. adopted a value for *M* which is 4.8 ppm smaller than the one we have used and they attributed to it an overall uncertainty of 5 ppm.

After we completed this assessment, we learned that Cohen and Taylor [1] also reevaluated Nier’s data. Their conclusions concerning the value of *M/γ*_0_ and its error are the last entries in table 8. Cohen and Taylor’s conclusions are much closer to our own than those of Quinn et al. [64].

## 8. The Pressure and Other Thermodynamic and Transport Properties

The “working equation” discussed in sections 2.8 and 2.9 demonstrates that the present determination of *R* requires measurements of the pressure and values for the thermal diffusivity of the gases; however, these quantities need not be known to nearly the same accuracy as the primary quantities (resonance frequencies, volume, temperature, and *M/γ*_0_). From the information presented below, we conclude that the error in the measurement of the pressure makes a negligible contribution to the error in *R*. We also conclude that the uncertainty in the value of the thermal conductivity obtained from the literature contributes 0.30 ppm to the error budget for *R* for the argon data.

We shall also cite expressions for the viscosities of the gases used. The viscosity was useful for comparing the measurements of the half-widths of the acoustic resonances with the theory of the resonator and might have revealed systematic errors.

### 8.1 Measurement of the Pressure

From the “working equation,” one can see that the sensitivity of *R* to errors in pressure measurements is greatest through the dependence of the speed of sound upon pressure. For argon (1/*c*^2^)·(d(*c*^2^)/d*p*) = 0.002 MPa^−1^. It follows that pressure measurements must have an imprecision no greater than 100 Pa to insure that this imprecision contributes no more than 0.2 ppm to the uncertainty in *R*. This certainly was achieved in the present work. The accuracy required of the pressure measurements is on the order of 200 Pa at 100 kPa and declines at higher pressures. (For helium (1/*c*^2^)·(d(*c*^2^)/d*p*)=0.01 MPa^−1^, a value 5 times larger than for argon. To determine *R* with helium, the bounds on the allowable imprecision and inaccuracy of the pressure measurements are 5 times smaller than for argon, under corresponding conditions.)

The pressure measurements were made with a fused-quartz-bourdon-tube differential pressure gage. This gage (Model No. 6000-801-1, Ruska Instrument Corporation [29]) uses an optical readout of the bourdon tube’s position and a magnetic feedback system. The manufacturer’s calibration data indicated that the gage had a full scale range of 1 MPa and was linear to 1 part in 10^s^. The reference side of the gage was continuously evacuated by a mechanical vacuum pump. The pressure on the reference side was monitored with a thermocouple-type vacuum gage and was in the range 1–2 Pa. The zero-pressure indication of the gage was found to change by as much as 27 Pa between checks; however, changes were generally much smaller. At the conclusion of all the measurements, the pressure gage was compared with a calibrated barometer. The gage read 6 Pa higher than the barometer.

As indicated in figure 16, the gas in the resonator and the manifold was always separated from the pressure gage by a diaphragm. This arrangement was required when helium was the test gas. (If helium at high pressures were to come in contact with the bourdon tube it would diffuse into the fused quartz and change the calibration in a time-dependent manner.) The separator arrangement was also convenient for preserving the cleanliness of the test gases. The separating diaphragm was a variable capacitance differential pressure transducer (Type 315BD-00100 sensor head with Type 270B electronic display unit, MKS Instruments, Inc. [29]) constructed of stainless-steel and inconel. The full-scale range of this unit was 13 kPa. Between checks, the zero-differential-pressure indication of the separator was found to change by as much as 53 Pa; however the changes were generally much smaller.

The pressure of the test gas was always measured while the valve atop the resonator was being closed. This was always done when the resonator was sufficiently near thermal equilibrium that its temperature could be defined with the accuracy required for the pressure measurements. We discovered that while the valve was being closed the pressure increased, as one might expect, from compressing the bellows stem seal of the valve. A small correction was made for this effect by plotting the indicated pressure as a function of the position of the valve’s handle and extrapolating the plot to the closed position.

### 8.2 Thermal Conductivity and Viscosity of the Gases

An error in the thermal conductivity, λ, of the gas leads to an error in the determination of *R* through the thermal boundary layer correction. The correction to *R* varies as λ and is 125 ppm, averaged over the (0,2)–(0,6) modes for argon at 100 kPa. The 0.3% uncertainty in λ led to a 0.30 ppm contribution to the error budget for *R* when *R* was determined from the argon data described in the next section. This error propagation was established by numerical experiments in fitting the weighted speed of sound data on the isotherm *T*_t_.

We have used the expression

$$\begin{array}{l} \lambda /({\text{Wm}}^{-1}K^{-1})=(1.6382+0.0052\{(T-T_{t})/K\})\\ \times 10^{-2}+216\times 10^{-5}(\rho /\text{kg}\cdot m^{-3}) \end{array}$$

for the thermal conductivity of argon. The zero-density values of the thermal conductivity were obtained from the “HFD-B2” potential by Aziz and Slaman [66]. As discussed by these authors, the thermal conductivity calculated from the potential is in agreement with recent measurements of the thermal conductivity near 300 K, within experimental errors which range from 0.2–0.3%. The same potential is consistent with the dispersion coefficient, spectroscopy, scattering, and bulk data. Our confidence in the derived thermal conductivity is enhanced by the fact that the same potential was used to calculate the viscosity and diffusion coefficients near *T*_t_ and these transport properties also agree with direct measurements within experimental errors on the order of 0.3%. The density coefficient of the thermal conductivity of argon is the value tabulated for 27.5 °C by Maitland et al. [67] and is based upon direct measurements. From the same two sources, we have obtained the expression for the viscosity of argon:

$$\eta /(\text{Pa}\cdot s)=(2.0973+0.0064\{(T-T_{t})/K\})\times 10^{-5}+1.111\times 10^{-8}(\rho /\text{kg}\cdot m^{-3})$$

The corresponding expressions for helium are:

$$\lambda /({\text{Wm}}^{-1}K^{-1})=(1.4573+0.00368\{(T-T_{t})/K\})\times 10^{-1}+2.91\times 10^{-4}(\rho /\text{kg}\cdot m^{-3})$$

and

$$\eta /(\text{Pa}\cdot s)=(1.8638+0.0046\{(T-T_{t})/K\})\times 10^{-5}-1.064\times 10^{-8}(\rho /\text{kg}\cdot m^{-3}).$$

Here, the zero-density values were obtained from reference [68] and the density coefficient was obtained from reference [67].

### 8.3 Density and Heat Capacity of the Gases

In order to make the correction for the thermal boundary layer, we require the density and heat capacity of the gases as functions of pressure. To obtain the density, we inverted the virial equation of state. To obtain the pressure dependence of the heat capacities, we also used the virial equation to obtain the relations:

$$\begin{array}{l} C_{p}=\frac{5}{2}\cdot R-T\cdot p\cdot \frac{d^{2}B}{dT^{2}}\\ \gamma =\frac{5}{3}\left(1+2(\gamma -1)\frac{dB}{dT}\cdot \frac{p}{R}+{\left(\gamma -1\right)}^{2}\cdot T\cdot \frac{d^{2}B}{dT^{2}}\cdot \frac{p}{R}\right). \end{array}$$

The second virial coefficient, *B*, and its temperature derivatives were calculated at *T*_t_ from the HFD-B2 potential [66] for argon. The results are

$$B/({\text{cm}}^{3}\cdot {\text{mol}}^{-1})=34.25-1.170\times 10^{4}(K/T)-9.56\times 10^{5}{\left(K/T\right)}^{2}$$

including the leading quantum corrections. For helium, the expression given by Guildner and Edsinger [69] was used:

$$B/({\text{cm}}^{3}\cdot {\text{mol}}^{-1})=12.00-0.0044\{(T/K)-2.73.15\}.$$

## 9. Determination of $c_{0}^{2}$ in the Working Gas

In this section we describe our speed-of-sound measurements and their analysis for the working gas Ar-M along the isotherm at *T*_t_. These measurements were Fitted to various models and they established $c_{0}^{2}=A_{0}=(94756.17_{8}\pm 0.06_{5})$ m^2^/s^2^ for the working gas. The 0.68 ppm imprecision in *A*_0_ from the fit includes the effects of imperfect measurements of the resonance frequencies, random errors in the temperature measurements, and imperfect thermal accommodation between the gas and the shell. The values of *A*_0_ determined from the resonance frequencies of the (0,2)–(0,6) modes, each fitted separately, were consistent, within the precision stated; thus the final value of *A*_0_ is based on an average of the the data for these five modes. At the end of this section, we discuss a possible problem in defining the location of one transducer during the determination of *A*_0_. This possible problem has led us to add an additional error term of 0.59 ppm to table 1, which could have been eliminated if the opportunity to repeat the measurements at *T*_t_ were available.

### 9.1 Preparation of the Resonator

The measurements which determined *A*_0_ were performed during the period of time between the thermometer calibrations of March and April 1986, and thus also lie between the second and third determination of the volume of the spherical resonator.

After the second volume determination, the resonator was carefully cleaned and “baked” for 2 days under vacuum to remove the last traces of mercury. The valve mechanism and transducers were installed. The resonator was mounted in the thermostat and connected to the gas handling system where it was evacuated and “baked” for a further period of 48 h. During this time the resonator was maintained at a temperature of 60 °C (approximately 25 °C below the softening temperature of the vacuum wax in the equatorial joint) while all remaining components accessible to the experimental gases were maintained at or above 100 °C. Residual gases in the vacuum system were monitored using the ionization gage and mass spectrometer. Initially, a considerable number of impurities with mass numbers up to 200 were evident and these could be traced, by closing valves, mainly to the resonator. We suspect that heavy hydrocarbon impurities were evolved from the wax joint at the elevated temperature. Towards the end of the bakeout all the high-mass impurity peaks showĕd considerable reduction and when the system was cooled down to *T*_t_ the total pressure reading fell from about 0.5 mPa to below 4 μPa. At the same time almost all of the remaining peaks in the mass spectrum disappeared leaving resolvable peaks at *m/e* = 1, 12, 14, 16, 17, 18, 19, 28, and 44 amu/*e* only. Of these, hydrogen had a relative intensity of 111, mass numbers 19 and 28 had intensity 10, and all others less than 3. Finally, the resonator and gas handling system were flushed with the working gas, Ar-M, up to a maximum pressure of 500 kPa (venting through the vacuum pumps) before filling with Ar-M for the first set of measurements.

### 9.2 Speed of Sound Measurements

The measurements considered in this section were performed in three separate runs. The first started at a pressure near 500 kPa and proceeded to lower pressures in steps of approximately 100 kPa; the second commenced at 450 kPa and continued down to 50 kPa in decrements of 100 kPa; and, the third started near 100 kPa and ended near 25 kPa with decrements of 25 kPa. At each point on the isotherm, the resonator was allowed to approach to within a few millikelvins of the thermal steady state before the pressure was measured and the valve atop the resonator closed. A further period of time was then allowed for the thermal transient caused by this operation to decay before the measurements proceeded. In every case, the steady-state temperature was within approximately 5 mK of *T*_t_ and was stable to better than 0.4 mK (typically 0.2 mK) during the period of about 45 min required to measure a complete set of resonance frequencies. The temperatures reported here are always the mean of those measured at the north and south poles (for the present measurements these correspond to thermometers #835B and #1888002, respectively). Frequency measurements were performed on the lowest six radial modes starting with (0,2) and working up to (0,7); they were then repeated in reverse order, finally ending with (0,2). (As discussed in sec. 4.3.1, the data for the (0,7) mode were omitted from the analysis, because they overlapped with the (13,2) mode.) During the frequency measurements for each mode, the resistances of both thermometers were measured so that every resonance frequency is associated with a unique determination of the mean temperature. At the completion of such a set, the valve between the resonator and the gas-handling system was opened and the pressure measurement was checked. Because the external pipework was not thermostatted, small changes (up to 50 Pa) were observed; however, they were inconsequential. The pressure was then reduced by pumping gas out through the vacuum system, and the temperature of the resonator (which fell by adiabatic cooling during the expansion) was restored as close as possible to *T*_t_ by supplying power to the equatorial heaters. After about 2 h the system again attained a steady state and was ready for the next point on the isotherm.

### 9.3 Speed of Sound Results

In Appendix 1 we list the measured resonance frequencies and half-widths, together with the corresponding averages of the polar temperatures, for each pressure studied. Appendix 1 includes two measurements of frequency and half-width for each of six modes at each of 14 pressures for a total of 168 frequencies and 168 half-widths. Speeds of sound at *T*_t_ and the experimental pressures were obtained from these data as follows. First the data for the (0,7) mode were discarded because of the mode overlap problem discussed in section 4.3.4. The resonance frequencies for the (0,2) mode were multiplied by the factor 1 +0.7×10^−6^ to correct for the shape perturbation resulting from the unequal diameters of the hemispheres, as mentioned in section 3.4. Then, all the resonance frequencies were corrected for the thermal boundary layer using eq (2.32) and for coupling of gas and shell motion using eqs (2.20) and (2.22), as well as the transport and equilibrium properties from sections 8.2 and 8.3. The thermal boundary layer correction requires a value for the thermal accommodation coefficient *h* in eq (2.24). At this point in the analysis (and in Appendix 2) we assumed *h* ≡ 1; however, this assumption is relaxed below. The corresponding speeds of sound at the experimental temperatures and pressures were then obtained using the volume of the spherical cavity (at the temperature and pressure of the measurement) given in section 6.1 and, finally, these speeds were corrected with negligible additional uncertainty to exactly *T*_t_ according to

$$c^{2}(T_{t}/p)=(T_{t},T)c^{2}(T,p).$$ (9.1)

The corrected values for the speed of sound in Ar-M at *T*_t_ are listed in Appendix 2.

### 9.4 Analytical Representations of the Speed of Sound

We have chosen to fit the speed of sound to the physically motivated expression

$$V^{2/3}{\left(f_{0,n}/v_{0,n}\right)}_{\text{corr}}^{2}-A_{3}p^{3}=c^{2}-A_{3}p^{3}=A_{0}+A_{1}p+A_{2}p^{2}+A_{-1}p^{-1}$$ (9.2)

from which we obtain *A*_0_ for Ar-M at *T*_t_ and ultimately the value of *R*. In using eq (9.2), we have assumed *A*_3_= 1.45×10^−18^ m^2^·s^−2^·Pa^−3^, the value obtained by Goodwin [28] from speed of sound data at pressures up to 7 MPa where this term contributes 500 ppm to *c*^2^. (The best value of *A*_3_ is 1.2×10^−18^ m^2^s^−2^Pa^−3^ if Goodwin’s data are fit subject to the constraint that *A*_1_ and *A*_2_ equal the values we report in table 11.) In any case, the contribution of *A*_3_ to the fit to our data is so small that we could have assumed *A*_3_*≡*0. If we had done so, *A*_0_ and *R* would have been increased by 0.42 ppm which is only 0.6 of the standard deviation of *A*_0_ and only 0.25 of the standard error in *R*.

Equation (9.2) was fit to the data using the least squares procedures published by Bevington [70]. Before fitting, the two values of *c*^2^ derived at a given pressure from a given radial mode were averaged yielding a total of 70 observations corresponding to the lowest five radial modes at the 14 pressures studied. Each observation was weighted inversely by the square of its estimated standard deviation which was taken to be the sum of two terms added in quadrature. The first term, 1.414×10^−7^*c*^2^(1 + (10^5^ Pa/*p*)^2^(6 kHz/*f*_0_,*_n_*)^2^), is an estimate for the errors in frequency measurements from eq (4.2). The second term in the estimated standard deviation is 3.7×l0^−7^*c*^2^. It was chosen such that χ^2^= 1 for a “good” fit as judged by deviation plots. If the second term were entirely the result of random errors in the measurement of temperature on the time scale of 4–8 h, it would correspond to a temperature error of 0.1 mK, which is consistent with the experimental procedures.

**Figure 17:**
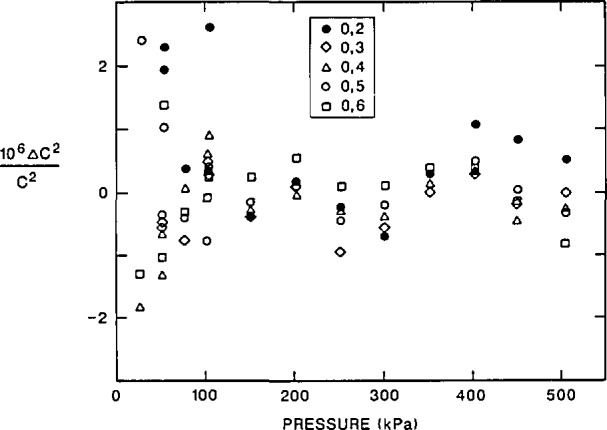
Fractional deviations of 70 observations of *c*^2^ from eq (9.2) with the parameters from column 1 of table 11. (Δ*c*^2^ = observed *c*^2^−calculated *c*^2^.) The symbols used to identify the various modes are indicated in the inset.

**Figure 18:**
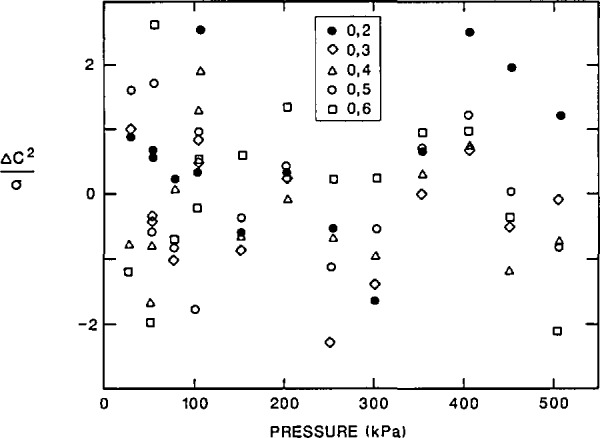
Deviations of 70 observations of *c*^2^ from eq (9.2) with the parameters from column 1 of table 11, in units of the pressure- and frequency-dependent standard deviation calculated in section 9.4. (Δ*c*^2^ = observed *c*^2^−calculated *c*^2^.) The symbols used to identify the various modes are indicated in the inset.

The fitted coefficients for eq (9.2) and their errors are listed in table 11, column 1. In each case, the error quoted is the diagonal element of the error matrix multiplied by the square root of *χ*^2^ divided by the number of degrees of freedom. In figure 17, we show fractional deviations of *c*^2^ for each mode from the fit. All but 13 of the 70 observations deviate by less than 1 ppm and 11 of these 13 are in the narrow region below 100 kPa where the signal-to-noise ratio is reduced. In figure 18 the deviations from the same fit are shown, now scaled by the pressure- and frequency-dependent standard deviation in the speed of sound. All 70 data fall within 2.6 standard deviations of the fit. There are no obvious trends in the deviations with pressure or frequency. The fit establishes *A*_0_ ( $c_{0}^{2}$ in table 1) with an imprecision of 0.68 ppm. This small imprecision is a direct consequence of the narrowness of radially symmetric resonances and the high signal-to-noise ratio obtained with the spherical resonator.

### 9.5 Discussion of the Isotherm Parameters *A*_−1_ *A*_1_, and *A*_2_

If the parameter *A*_−1_, is interpreted as arising solely from imperfect thermal accommodation, (and not from an impurity of fixed partial pressure) its value in column 1 is equivalent to an accommodation coefficient *h* =0.93±0.07. This value is consistent with the value *h* =0.84±0.05 obtained by Ewing, McGlashan, and Trusler [10] in studies of argon in an aluminum resonator at pressures ranging from 15–248 kPa. Shields and Faugh also report that thermal accommodation between heavy molecules and machined metal surfaces is very efficient [22].

We have used the value *h* =0.93 (obtained from the value of *A*_−1_ in table 11, column 1) to redetermine *c*^2^ from the resonance frequencies and then refit eq (9.2) to the revised values of *c*^2^. In doing so, we have relaxed the assumption *h* ≡ 1 made above. The changes in *A*_0_, *A*_1_, and *A*_2_ were negligible.

If the speed-of-sound data are fitted with the constraint *A*_−1_≡0, (or, equivalently, the accommodation coefficient is constrained to be exactly 1) then *A*_0_ is increased by 0.59 ppm and *χ*^2^ is increased by 0.2%. (Table 11, column 2.) As expected, this constraint greatly reduces the correlation between the remaining parameters; thus, statistical measures of their imprecision are much smaller; however, there is no a priori reason for assuming *A*_−1_ is exactly 0.

Before comparing the value of *A*_1_ with independent values, we mention two sources of systematic error in this parameter. First, in section 3.8 it was shown that uncertainty in the shell’s compliance (±6%) contributed an uncertainty of ±0.09% in *A*_1_. Second, we have not corrected the data for the compliance of the transducers. We now argue that this correction increases *A*_1_ by 0.13% and the systematic error in this correction is much smaller than ±0.13% of *A*_1_.

We used the value 1.1× 10^−10^ m·Pa^−1^ for the transducers’ compliance in argon at *T*_t_ throughout the pressure range 25–500 kPa. This compliance reduces the resonance frequencies by 0.83 ppm at 500 kPa and proportionately less at lower pressures. This value of the compliance is based on information provided by the manufacturer [31] and is correct when the transducers are in air at 20 °C and 100 kPa and are used at frequencies well below their resonance at 40 kHz. Under these conditions the air inside the transducers provides only 10% of their stiffness; the tension in their diaphragms provides the rest. Thus, replacing the air by argon and reducing the pressure below 100 kPa has little effect on the compliance. Increasing the pressure of the air from 100 kPa to 500 kPa reduces the compliance by 30% [31]. We neglect these effects, thereby introducing a systematic error in the compliance which is a fraction of the compliance. Our estimate of the compliance of the transducers is equivalent to 9.5% of the static compliance of the shell. We corrected the data in Appendix 1 for this additional compliance, recalculated the speed of sound from each frequency, and repeated the fit of eq (9.2) to the data. As expected c_0_^2^ was unchanged; *A*_1_ was increased by 0.13% to (2.2533±0.0035) × 10^−4^ m^2^·s^−2^Pa^−1^. The changes in *A*_2_ and *A*_−1_, were negligible. For comparison with other work we shall use this corrected value of *A*_1_ and we shall enlarge the estimate of its standard error to 0.19%. The standard error was calculated adding in quadrature the standard deviation of *A*_1_ from the fit (0.16%), the effect of the uncertainty in the shell’s compliance (0.09%), and 1/3 of the effect of the transducers’ compliance (0.04%). We note that all previous determinations of *A*_1_ from acoustic resonances were influenced by the compliances of the resonators and the transducers; however, the consequences of these compliances were usually ignored.

Several independent determinations of *A*_1_ exist and these may be compared by considering the, perhaps more familiar, second acoustic virial coefficient *β*_a_=(*M/γ*_0_)*A*_1_. Firstly there are direct acoustic measurements, secondly estimates based on the volumetric second virial coefficient *B*, using

$$\beta_{a}=2B+\frac{4}{3}T\frac{dB}{dT}+\frac{4}{15}T^{2}\frac{d^{2}B}{dT^{2}},$$ (9.3)

and finally calculations of *β*_a_ based on intermolecular pair potentials *U*(*r*) and

$$\beta_{a}=4\pi N_{A}\int_{0}^{\infty }[1-\{1+\frac{2}{5}(U/kT)+\frac{2}{15}{\left(U/kT\right)}^{2}\}\exp(-U/kT)]r^{2}dr.$$ (9.4)

(*N*_A_ is Avogadro’s constant.) In the first category, the results of Colclough et al. [13] yield *β*_a_=(4.84±0.07) cm^3^·mol^−1^, while the precise measurements of Ewing et al. [10] using a spherical resonator at pressures up to 250 kPa give *β*_a_=(5.24±0.06) cm^3^·mol^−1^. The difference between the present value *β*_a_=(5.401 ±0.010) cm^3^·mol^−1^ and that of Ewing et al. [10] is about twice the combined standard deviation but nevertheless still remarkably small.

Estimates of *β*_a_ from (*p, V*_m_, *T*) measurements were obtained by Rowlinson and Tildesley [71] and range from 4.9_6_ cm^3^·mol^−1^ to 5.9_9_ cm^3^·mol^−1^, encompassing the present result.

We have calculated *β*_a_ from eq (9.4) using the most recent determination of *U*(*r*) (the HFD-B2 function of Aziz and Slaman [66]) and also, for purposes of comparison, an earlier determination (the HFD-C function [72]). Contrary to the assertion of Rowlinson and Tildesly [71], we find that the leading quantum correction to *β*_a_ makes a small but significant contribution (0.16 cm^3^·mol^−1^) at *T*_t_. Accordingly, we have combined this term with the results obtained with eq (9.3) to give *β*_a_=5.15 cm^3^·mol^−1^ from the HFD-B2 and *β*_a_ = 5.51 cm^3^·mol^−1^ from the HFD-C. The uncertainty associated with these values is difficult to assess but must be on the order of the difference between them since the differences between the functions themselves are slight. We regard the agreement with our value as excellent.

Clearly, these independent estimates of *β*_a_ for argon at *T*_t_ are in close agreement by the standards of the most precise measurements of virial coefficients and all lie close to our determination. Nevertheless, the differences among the values corresponds to about 25 ppm in *c*^2^ at the lowest pressure of our experiment.

**Figure 19:**
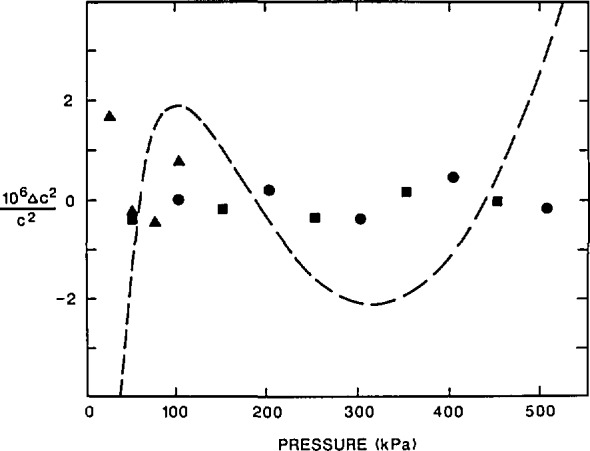
Fractional deviations Δ*c*^2^/*c*^2^ of *c*^2^ from eq (9.2) with the parameters from column 1 of table 11. The 14 points represent the deviations of the mean value of *c*^2^ computed for the five modes at each of the 14 pressures. Each of the three runs mentioned in section 9.2 is distinguished by a different symbol. The dashed curve represents eq (9.2) with the parameters which best fit the data, except for *A*_2_ which was constrained to be 6.96×10^−11^ m^2^s^−2^Pa^−2^, the value obtained in reference [13].

Our data constitute the most precise set ever obtained for *c*^2^ in argon at *T*_t_ and pressures up to 500 kPa; thus we must look to measurements at higher pressures for a value of *A*_2_ of comparable precision. We considered the measurements of Colclough et al. [13] which extend up to 1.3 MPa and yield *A*_2_ = (6.96±0.37)× 10^−11^ m^2^·s^−2^·Pa^−2^. Unfortunately, we find this value to be inconsistent with our data as shown in figure 19. Here the dashed curve represents the function obtained when *A*_0_, *A*_1_, and *A*_−1_ were fit to the 70 observations, with *A*_2_ constrained to be the value of Colclough et al. The deviations are large compared with our imprecision. We note that two groups (Colclough et al. [13] and Ewing et al. [10]) obtained values of *A*_2_ that are greater than the present value and values of *A*_1_ that are smaller. Perhaps these differences are correlated to some degree. Note added in proof: The same isotherm from which we obtained *A*_3_ yields *β*_a_=(5.464±0.008) cm^3^ mol^−1^ and *A*_2_= (5.084±0.014)× 10^−11^ m^2^s^−2^ Pa^−2^. This isotherm [28] extending to 7 MPa had less correlation between terms and is closer to ours.

Previous measurements of *A*_1_ and *A*_2_ are not accurate enough to use in extrapolating our measurements to the limit *p*= 0 without significant loss of accuracy in *A*_0_. Furthermore, one must assume that the accommodation coefficient is an apparatus-dependent parameter; thus the parameter *A*_−1_ must also be fit to our data. We conclude that fitting eq (9.2) to the data in Appendix 2 is the best method of determining *A*_0_.

### 9.6 Possible Transducer Location Problem

In the early sections of this manuscript, we have discussed three factors increasing the inaccuracy of *A*_0_ beyond the 0.68 ppm imprecision resulting from the fit to the isotherm. These factors were the uncertainty in the volume, the uncertainty in the thermal boundary layer correction arising from the uncertainty in the transport properties, and the errors in temperature measurements. The contributions to these items to the error budget appear in table 1. We now consider another possible source of systematic error which appears in table 1 as “possible error in location of transducers.”

Upon disassembly of the apparatus after the speed-of-sound measurements at *T*_t_ and just prior to the final volume determination, we found that a bolt used to hold one of the transducer assemblies in the shell was binding. (The bolt was too long for the blind tapped hole.) The binding may have prevented the transducer assembly from seating correctly in place, with a possible error in the volume of the cavity. Mechanical measurements on its location were difficult (in the absence of a proper reference plane) and ambiguous at the level of 1 ppm. Accordingly, after the third volume determination in April 1986, the carefully cleaned resonator was reconfigured for acoustic measurements with the transducers correctly installed. Because of the possibility of a volume change occurring as a result of heating, the resonator was “baked” at only 35 °C (the remainder of the system was baked as before). Six sets of measurements on three different samples of Ar-M (taken directly from the cylinder) were obtained near *T*_t_ and 100 kPa. For each set, the mean value of *c*^2^ was computed from data for the (0,2) through (0,6) modes weighted according to eq (4.2). The results are given in table 12 together with the fractional deviations of *c*^2^ from the four-term fit to eq (9.2). Also shown in table 12 are the results of the two sets of measurements near 100 kPa which were part of the isotherm at *T*_t_ used in the fit to eq (9.2). With one exception, the data after re-seating the transducer are within 1 ppm of the data used to fit eq (9.2). The average difference between the sets of data is 0.59±0.76 ppm, or about 0.87 standard deviation of *A*_0_. (If the first line of table 12 were omitted, the difference would be 0.34±0.45 ppm.) We conclude that the error in the volume, if any, is within the imprecision of our measurements and neglect it. However, we include a contribution of 0.59 ppm in the estimated uncertainty of *R* arising from this problem.

## 10. Other Tests for Systematic Errors

The present determination of *R* requires an understanding of the behavior of a spherical acoustic resonator to a degree which has not been demonstrated heretofore. Thus, any indication of possible systematic errors is relevant to evaluating the spherical resonator method. In this section we shall report the differences between the experimental and theoretical values for the half-widths of the acoustic resonances. At the lowest pressures, this measure of our incomplete understanding of the present spherical resonator approaches zero. In the range of the measurements it is smaller than, but comparable to, the uncertainty in *A*_0_ discussed in the preceding section. We shall also mention small internal inconsistencies of the resonance frequencies measured with helium in the shell. They do not indicate important systematic errors in the acoustic model.

### 10.1 Excess Half-Widths

**Figure 20:**
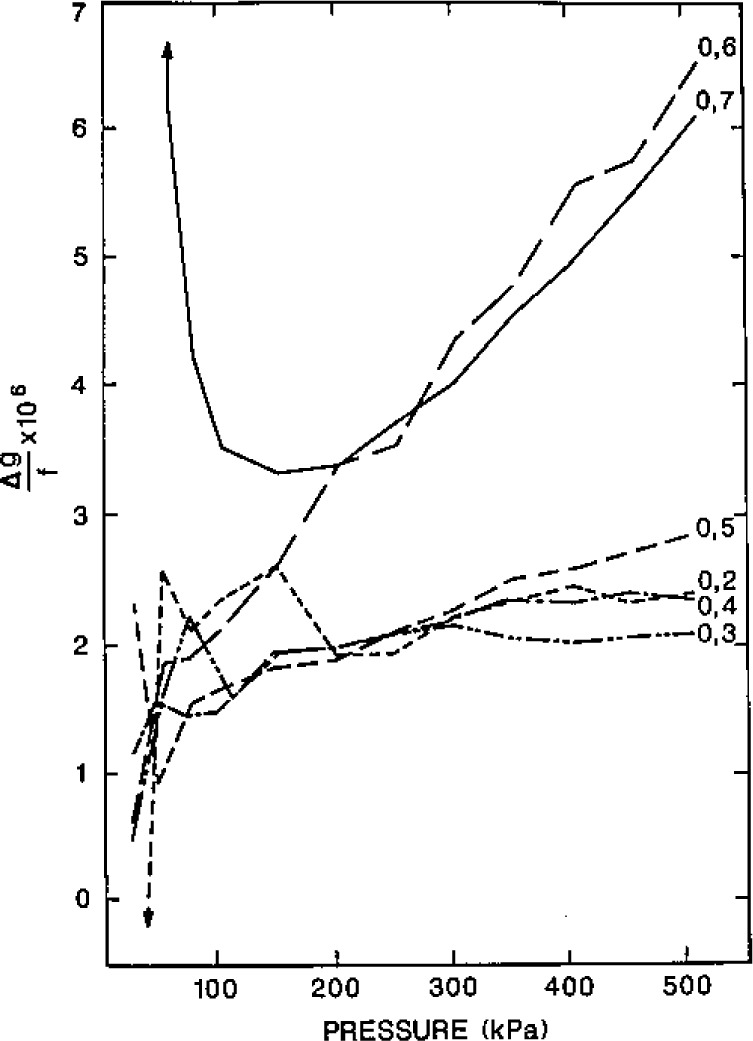
Excess half-widths of (0,*n*) resonances with argon in the resonator scaled by 10^6^/frequency. Δg= measured *g* minus calculated *g*.

The half-widths of the resonances are calculated without fitted parameters from eq (2.32) using the transport properties listed in section 8.2. The excess half-width, Δ*g*, is defined as the amount by which a measured half-width exceeds the calculated half-width. Figure 20 displays Δ*g* for measurements in Ar-M at *T*_t_ as a fraction of the measured frequency in parts per million. At low pressures, the Δ*g/f* of the (0,7) mode increases sharply as a result of the overlap of this mode with the neighboring (13,2) mode. For the other modes which were used in the determination of *A*_0_, Δ*g/f* decreases with decreasing pressure and appears to approach 1 ppm near zero pressure. To prepare figure 20, we used the same 8-parameter fits of eq (4.1) to the detector-amplitude vs frequency data which we used to determine the speed of sound and *A*_0_. If we had used 6-parameter fits, Δ*g/f* would be 0.5–1 ppm smaller for almost all the data displayed in figure 20. We have no explanation for the differing results with the differing fitting procedures and we did not encounter this difference when helium was in the resonator.

**Figure 21:**
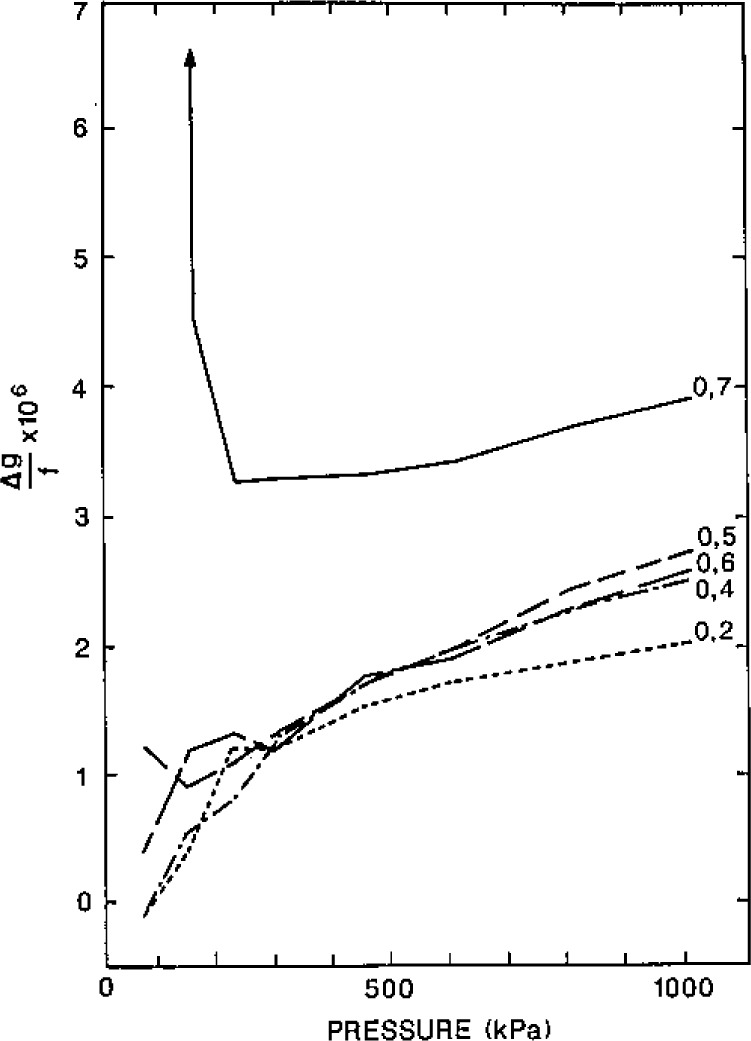
Excess half-widths of (0,*n*) resonances with helium in the resonator scaled by 10^6^/frequency. Δg = measured *g* minus calculated *g*.

Figure 21 displays the Δ*g/f* for five modes with helium in the resonator. The excess half-width of the (0,3) mode is not displayed; it varies from 5–17 ppm over the 75–1003 kPa pressure range studied. These unusually large values of Δ*g* for the (0,3) mode undoubtedly occur because the (0,3) resonance in helium at *T*_t_ is at 13.4 kHz, in accidental coincidence with the frequency of the breathing resonance of the shell.

The similarity of figure 20 to figure 21 is particularly striking upon recalling that the resonance frequencies are 3.162 times higher with helium than with argon. As for argon, Δ*g/f* of the (0,7) mode in helium increases sharply at low pressures and is consistent with our interpretation of this effect as being a result of the overlap with the (13,2) mode. As for the argon data shown in figure 20, Δ*g/f* of the well separated modes in helium decreases with decreasing pressure and never exceeds 3 ppm. In contrast with argon, Δ*g/f* for helium is the same for 6-parameter and 8-parameter fits to data for each resonance.

**Figure 22:**
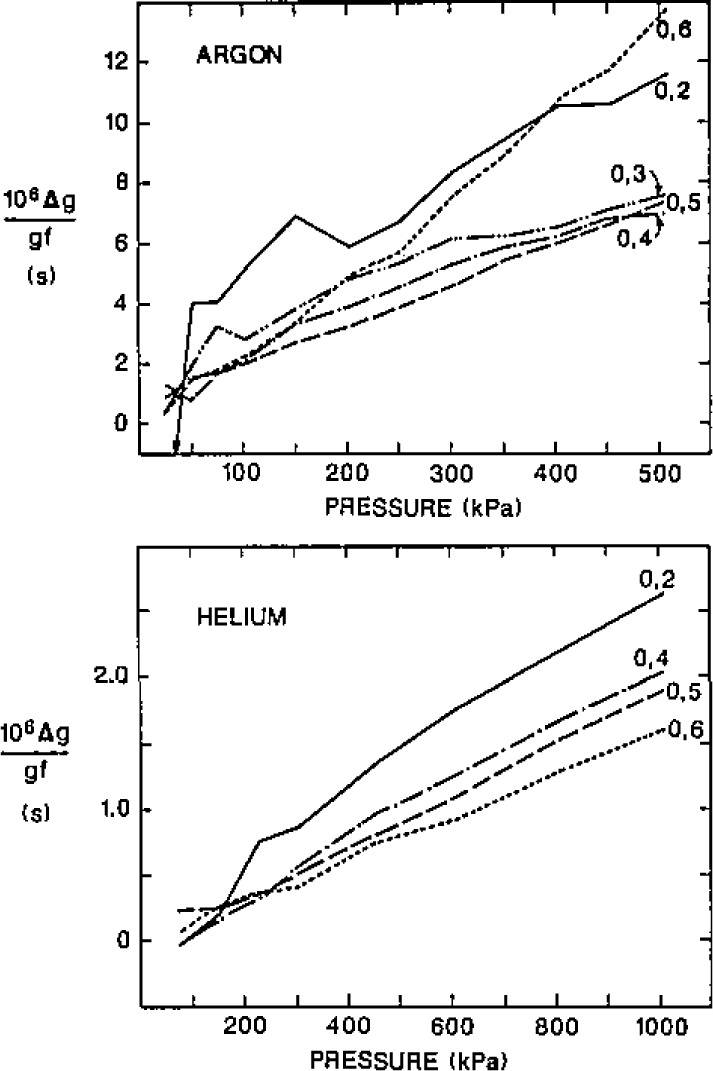
Excess half-widths of (0,*n*) resonances with argon (top) and helium (bottom) in the resonator scaled by 10^6^/(frequency × hulf-width). Δ*g* = measured *g* minus calculated *g*.

In figure 22, we have replotted Δ*g/f* scaled by 10^6^/*gf* and omitted the data for the (0,7) mode. It is encouraging that as the pressure is reduced, Δ*g/f* clearly approaches zero for both gases. We have no explanation for the pressure and frequency dependences of Δ*g*. These dependences are not consistent with the addition of a phenomenological damping term to the equation of motion for the shell or with a model for the acoustic radiation from the outer surface of the shell into the surrounding gas (which was nearly the same as the gas within the shell [9]).

We interpret the fact that Δg does approach zero for both gases as providing an important check of the boundary layer correction to the resonance frequencies. If either the acoustic model or the thermal conductivities were seriously in error, Δ*g* would not be small. The same model and data are used in the calculation of the thermal boundary layer correction; thus, we believe that the calculation has firm experimental support.

### 10.2 Acoustic Resonances in Helium

We have mentioned that the acoustic measurements with helium in the shell showed evidence of progressive contamination which made them unsuitable for the most accurate determination of *R*. The contamination rate was sufficiently low that *relative* frequency measurements could be used to test the understanding of the resonator’s performance at higher frequencies which straddle the breathing mode of the shell near 13 kHz. As the pressure was decreased from 1.0 MPa to 0.22 MPa, the rms deviation of *c*^2^ from its mean (as derived from the (0,2), (0,4), (0,5), and (0,6) modes) decreased from 2.4 ppm to 1.5 ppm, depending rather sensitively on the frequency used in the model of the breathing mode of the shell. The deviations were systematic. For example, the (0,6) mode consistently yielded values of *c*^2^ which were 2–3 ppm higher than the values of *c*^2^ from the (0,2) mode. We do not consider these systematic deviations to be a cause for concern. They were only 1/10 as large as the frequency shifts caused by contamination and probably indicate dispersion in the contaminated helium as well as failure of the simple, one-breathing-mode model of the shell’s elastic response at high frequencies, where the shell has many modes of vibration [7].

## 11. Summary

We have measured the volume of a spherical resonator at the temperature *T*_t_ by weighing the mercury required to fill it. The result was (2943.1524± 0.0036) cm^3^. Upon converting this volume from the weighing configuration to that used for acoustic measurements, the volume was increased by (0.0108±0.0005) cm^3^. The total uncertainty in the volume of 1.22 ppm includes all known systematic and random effects and it contributes just 0.80 ppm to the uncertainty in the redetermination of *R*. The compliance of the resonator was studied by the application of internal pressure when it was filled with mercury, by acoustically measuring the volume change upon reduction of external pressure, and by measuring some of the resonant frequencies of the shell. This well characterized spherical resonator was used to measure the pressure dependence of the speed of sound at the temperature *T*_t_ in the working gas, a commercially supplied sample of argon. The speed-of-sound data were fitted and extrapolated to zero pressure, thus determining *A*_0_=(94756.178±0.065) m^2^s^−2^ at *T*_t_ for the working gas. The error of 0.95 ppm includes a random component of 0.68 ppm from fitting the isotherm and a systematic component of 0.59 ppm from a possible problem in determining the location of the transducers during the measurements. An additional systematic uncertainty of 0.30 ppm results from the uncertain thermal conductivity’s effects on the correction to the measured resonance frequencies for the thermal boundary layer. The temperature of the speed-of-sound measurements is subject to possible systematic errors from calibration and from temperature gradients in the resonator. This contributes terms of 0.8 ppm and 0.4 ppm to the standard error in *R*. The heat capacity ratio at zero pressure, *γ*_0_, was taken to be exactly 5/3 for all of the argon samples used. The molar mass of the working gas was determined by comparing it to a special lot of argon whose chemical and isotopic composition was defined well enough to determine *M* to 0.7 ppm. The comparison used a series of extremely precise speed-of-sound ratio measurements at *T*_t_ and *p*_0_. The comparison showed that *M/γ*_0_= (23.968684±0.000019)g/mol for the working gas. Most of the 0.8 ppm uncertainty in *M/γ*_0_ came from gas chromatographic measurements of noble gas impurities in the special lot of argon. These results were combined to re-determine the universal gas constant *R* =(8.314471±0.000014) J·mol^−1^·K^−1^ with an uncertainty of 1.7 ppm.

We now consider an alternative statement of the uncertainty in the present redetermination of *R*. The form of the statement was inspired by an anecdote repeated by Dr. H. Ku, a statistician retired from NBS. Ku’s remarks at a “Round-table discussion on statement of data and errors,” [73] include:

“…In the 1930’s, C. H. Meyers et al. conducted an elaborate experiment to determine the specific heat of ammonia. After several years of hard work, they completed the experiment and wrote a paper reporting their results. Toward the end of their paper, Meyers declared: “We think our reported value is good to 1 part in 10000: we are willing to bet our own money at even odds that it is correct to 2 parts in 10000. Furthermore, if by any chance our value is shown to be in error by more than 1 part in 1000, we are prepared to eat the apparatus and drink the ammonia.”

Paraphrasing Meyers, we are willing to bet our own money at even odds that our reported value is correct to 5 parts in 10^6^, and if by any chance our value is shown to be in error by more than 10 parts in 10^6^, we are prepared to eat the apparatus, drink the mercury, and breathe the argon!

## Figures and Tables

**Table 1:** One-sigma uncertainties (in parts per million) from various sources in the redetermination of *R*

I (Volume)^2/3^	
density of mercury at 20 °C	0.28
storage and handling of mercury	0.20
thermal expansion of mercury (0–20 °C)	0.67
random error of volume measurements	0.20
corrections from weighing configuration to acoustics configuration	0.10
mass of counterweights	0.14
II Temperature	
random error of calibrations	0.8
temperature gradient	0.4
III *M/γ*_0_	
Ar-40 standard	0.7
comparison of working gas to Ar-40	0.4
IV Zero-pressure limit of (*f*_0_*_n_/v*_0_*_n_*)^2^	
s.d. of $c_{0}^{2}$ from 70 observations at 14 pressures	0.68
thermal boundary layer correction (0.3% of thermal conductivity)	0.30
possible error in location of transducers	0.55

Square root of the sum of the squares	1.7

**Table 2:** Key dimensions of the hemispheres

Blank	Outside radius, *r*_c_, mm	Inside radius, *r*_s_, mm	rms radial deviation $<\delta r_{s}^{2}{>}^{1/2}$, μm	Concentricity		Cylindrical extension *h*, mm
				δ*x*, *μ*m	δ*y*, *μ*m	
			After final polish			
#1	107.958	88.918	10	8	5	0.570
#2	107.963	88.938	4	1	1	0.509
			Before final polish			
#1	107.949	88.922	5	8	−4	0.537
#2	107.955	88.938	5	2	−5	0.497

**Table 3:** Properties of shell

Density	7.96 g/cm^3^
Internal radius	88.9 mm
External radius	108 mm
Young’s Modulus	197 GPa
Poisson ratio	0.297

**Table 4:** Thermometer calibration data at *T*_t_

Date	Thermometer	*R* (*T*_t_, *i*→0) Ω	Δ*R*/Δ*i*^2^ μΩ/mA^2^
June 5, 1986	1888002	25.541645	41
Apr. 17, 1986	1888002	25.541634	40
Apr. 2, 1986	1888002	25.541660	40
Mar. 25, 1986	1888002	25.541625	43
Oct. 10, 1985	1888002	25.541647	48
Apr. 16, 1986	835B	25.915752	72
Apr. 2, 1986	835B	25.915780	80
Mar. 25, 1986	835B	25.915751	79
Oct. 10, 1985	835B	25.915740	86
June 5, 1986	1818362	25.562715	116
Apr. 17, 1986	1818362	25.562703	115
Apr. 4, 1986	1818362	25.562750	116
June 5, 1986	303	25.475649	13
Apr. 17, 1986	303	25.475653	12
Apr. 1, 1986	303	25.475669	14

**Table 5:** Summary of thermometer characteristics

Thermometer serial number	1818362	1888002	835B	303
Mean self heating (μΩ/mA^2^)	116	42	79	13
*α*·10^3^	3.92719_6_^b^	3.92677_1_^b^	3.92575_8_^b^	3.92652_4_^b^
δ	1.49612^a^	1.49627^a^	1.49627^c^	1.49638^a^

a From calibration by NBS Temperature Section.

b From calibration by us at *T*_t_ and the Gallium Point.

c Assumed.

**Table 6:** Three measurements of resonator’s volume, *V*_R_

Fill #	1	2	3
Date	1985 Sept.	1985 Sept.	1986 Apr.
*T*(K)	273.159	273.158	273.158
*p*_equalor_(kPa)	101.8	101.1	101.0
*h*_1_(mm/kPa)	−0.2101	−0.2099	−0.2086
*m*_in_ (g)	40014.158	40014.153	40014.251
*m*_out_ (g)	40014.118	40014.117	
*V*_R_(*T*_0_, *P*_0_)(cm^3^)	2943.1540	2943.1515	2943.1518

**Table 7:** Data for correction of volume change from configuration change

Active transducer	Passive transducer	Plug	δ*V*, mm^3^
1	2	1	1.3_8_
1	2	2	1.0_3_
2	1	1	11.3_6_
2	1	2	11.1_4_

δ*V = V*(transducer) − *V*(plug).

**Table 8:** Values of *M/γ*_0_ for various gases

Gas	*M/γ*_0_ in g/mol
Ar-40 (standard, purified >26 h)	23.97751_1_ (±0.7 ppm)
Ar-M (working, commercial)	23.968684 (±0.8 ppm)
Ar-A (commercial)	23.96867_0_ (±0.8 ppm)
Ar-cominercial, from Nier^a^	23.96867 (±2.0 ppm)
Ar-commercial, from Nier^b^	23.96865 (±1.9 ppm)

a Re-evaluated in this work.

b Re-evaluated in reference [1].

**Table 9:** Sensitivity of $c_{0}^{2}$ to impurities

Impurity	*M* (g/mol)	*γ*_0_	$\frac{1}{c_{0}^{2}}\frac{d(c_{0}^{2})}{dx}$	
			in He	in Ar
H_2_	2	1.4	0.23	0.68
He	4	5/3		0.9
H_2_O	18	1.32	−3.93	0.12
Ne	20	5/3	−4.0	0.5
N_2_	28	1.4	−6.27	0.03
O_2_	32	1.4	−7.3	−0.07
Ar	40	5/3	−9.0	
CO_2_	44	1.4	−10.3	−0.37
Kr	84	5/3	−20.0	−1.1
Xe	131	5/3	−31.8	−2.3
Hg	201	5/3	−49.0	−4.0

**Table 10:** Speed of sound ratio determinations

Gas	Comment	$10^{6}\left(\frac{c(\text{gas})}{c(\text{Ar-M})}-1\right)$	Pressure (kPa)	Date
Ar-A		0.22	115	May 1, 1987
Ar-A		0.27	151	May 2, 1987
Ar-A		0.35	117	May 21, 1987
Ar-40	unprocessed	−191.5^a^	105	May 5, 1987
Ar-40	purified 26 h	−184.63	105	May 4, 1987
Ar-40	purified 120 h	−183.92	131	May 14, 1987
Ar-40	purified 240 h	−184.35	117	May 20, 1987
Ar-40	purified 240 h	−184.00	104	May 22, 1987

a The value listed is the mean determined from the (0,2)–(0,6) modes. The rms deviation from the mean for a single ratio was 1.0 ppm for the unprocessed Ar-40 and about 0.1 ppm for all other cases.

**Table 11:** Results of fits to speed-of-sound isotherm at *T*_t_

Parameter/Unit	1	2
*A*_0_/m^2^s^−2^	94756.178±0.065	94756.234±0.023
10^4^*A*_1_/m^2^s^−2^Pa^−1^	2.2502±0.0035	2.2476±0.0019
10^11^*A*_2_/m^2^s^−2^Pa^−2^	5.321±0.062	5.357±0.034
10^−3^/*A*_−1_/m^2^s^−2^Pa	2.7±2.9	0
χ^2^	1.30	1.30

**Table 12:** Measurements of the speed of sound in Ar-M at *T*_t_ near 100 kPa

*c*^2^/(m/s)^2^ −94779	*p*/kPa	Date	$10^{6}(c^{2}-c_{\text{calc}}^{2})/c^{2}$
Transducers correctly installed			
0.869	101.85	May 8, 1986	2.04
0.573	101.29	May 8, 1986	0.30
0.572	101.29	May 9, 1986	0.28
0.842	102.25	May 9, 1986	0.74
0.839	102.25	May 10, 1986	0.71
0.833	102.25	May 11, 1986	0.56

Transducer position uncertain			
0.293	100.26	Mar. 30, 1986	−0.10
0.607	101.37	Apr. 4, 1986	0.46

## Appendix 1. Resonance Frequencies and Half-Widths for Ar-M near *T*_t_

Data from three separate fillings of the resonator are tabulated. For each filling, the data were taken in order of descending pressure. The data from the first filling were taken March 29–30, 1986; the data from the second filling were taken March 31–April 1, 1986; and, the data from the third filling were taken April 4–5, 1986. Each of the 14 subtables are headed by the pressure and the average temperature at which the frequencies are measured. In each sub-table, the frequencies appear in the order in which they were measured.

**Table:**

Mar 29, 1986 *p*/kPa=504.177 *T*/K = 273.1637	
*f*/Hz	*g*/Hz
2477.6853	0.2098
4259.8189	0.2785
6012.7468	0.3368
7756.4083	0.3923
9495.9044	0.4757
11233.0132	0.5238
11233.0110	0.5237
9495.9051	0.4757
7756.4092	0.3923
6012.7475	0.3370
4259.8194	0.2782
2477.6853	0.2103

**Table:**

Mar 30, 1986 *p*/kPa=299.937 *T*/K = 273.1595	
*f*/Hz	*g*/Hz
2476.8988	0.2681
4258.4932	0.3567
6010.8968	0.4309
7754.0431	0.4974
9493.0404	0.5796
11229.6957	0.6392
11229.6960	0.6392
9493.0411	0.5796
7754.0440	0.4974
6010.8979	0.4309
4258.4941	0.3567
2476.8995	0.2681

**Table:**

Mar 29, 1986 *p*/kPa=401.361 *T*/K=273.1613	
*f*/Hz	*g*/Hz
2477.2870	0.2338
4259.1434	0.3101
6011.8041	0.3748
7755.2022	0.4345
9494.4431	0.5163
11231.3188	0.5668
11231.3164	0.5674
9494.4431	0.5172
7755.2033	0.4345
6011.8054	0.3751
4259.1466	0.3091
2477.2882	0.2340

**Table:**

Mar 30, 1986 *p*/kPa=199.894 *T*/K = 273.1646	
*f*/Hz	*g*/Hz
2476.5410	0.3250
4257.9010	0.4338
6010.0790	0.5236
7753.0041	0.6050
9491.7888	0.6958
11228.2544	0.7734
11228.2537	0.7730
9491.7879	0.6966
7753.0042	0.6049
6010.0782	0.5241
4257.9019	0.4341
2476.5411	0.3259

**Table:**

Mar 30, 1986 *p*/kPa= 100.261 *T*/K = 273.1630	
*f*/Hz	*g*/Hz
2476.0952	0.4583
4257.1896	0.6103
6009.1145	0.7388
7751.7880	0.8577
9490.3337	0.9815
11226.5840	1.1059
11226.5860	1.1062
9490.3381	0.9805
7751.7894	0.8588
6009.1144	0.7381
4257.1906	0.6087
2476.0971	0.4576

**Table:**

Mar 31, 1986 *p*/kPa=350.096 *T*/K = 273.1608	
*f*/Hz	*g*/Hz
2477.0926	0.2497
4258.8180	0.3303
6011.3492	0.4004
7754.6204	0.4634
9493.7385	0.5428
11230.5055	0.5990
11230.5037	0.6004
9493.7399	0.5427
7754.6220	0.4632
6011.3506	0.4003
4258.8197	0.3310
2477.0943	0.2489

**Table:**

Apr 1, 1986 *p*/kPa= 149.792 *T*/K=273.1650	
*f*/Hz	*g*/Hz
2476.3359	0.3768
4257.5694	0.5002
6009.6255	0.6043
7752.4332	0.6996
9491.1034	0.7987
11227.4662	0.8948
11227.4656	0.8955
9491.1025	0.7970
7752.4330	0.6994
6009.6259	0.6053
4257.5696	0.4989
2476.3366	0.3764

**Table:**

Mar 31, 1986 *p*/kPa=449.295 *T*/K = 273.1642	
*f*/Hz	*g*/Hz
2477.4801	0.2213
4259.4705	0.2939
6012.2592	0.3560
7755.7852	0.4130
9495.1496	0.4939
11232.1376	0.5437
11232.1351	0.5443
9495.1494	0.4920
7755.7849	0.4128
6012.2581	0.3560
4259.4698	0.2936
2477.4799	0.2217

**Table:**

Mar 31, 1986 *p*/kPa=250.250 *T*/K = 273.1585	
*f*/Hz	*g*/Hz
2476.7070	0.2919
4258.1715	0.3884
6010.4524	0.4696
7753.4758	0.5425
9492.3570	0.6248
11228.9056	0.6937
11228.9056	0.6956
9492.3579	0.6239
7753.4789	0.5423
6010.4551	0.4699
4258.1743	0.3895
2476.7085	0.2918

**Table:**

Apr 1, 1986 *p*/kPa=50.041 *T*/K = 273.1654	
*f*/Hz	*g*/Hz
2475.7768	0.6511
4256.7109	0.8687
6008.4901	1.0571
7751.0437	1.2330
9489.4574	1.4198
11225.5861	1.6461
11225.5763	1.6474
9489.4455	1.4162
7751.0412	1.2398
6008.4925	1.0564
4256.7098	0.8664
2475.7762	0.6501

**Table:**

Apr 4, 1986 p/kPa =101.371 7YK = 273.1740	
*f*/Hz	*g*/Hz
2476.1523	0.4570
4257.2843	0.6075
6009.2473	0.7337
7751.9632	0.8543
9490.5428	0.9702
11226.8343	1.1015
11226.8343	1.1015
9490.5459	0.9682
7751.9624	0.8545
6009.2471	0.7345
4257.2830	0.6053
2476.1560	0.4554

**Table:**

Apr 5, 1986 *p*/kPa=50.017 *T*/K = 273.1656	
*f*/Hz	*g*/Hz
2475.7762	0.6433
4256.7113	0.8696
6008.4930	1.0629
7751.0380	1.2289
9489.4720	1.4234
11225.5943	1.6433
11225.5867	1.6477
9489.4586	1.4182
7751.0401	1.2378
6008.4885	1.0606
4256.7119	0.8662
2475.7788	0.6504

**Table:**

Apr 4, 1986 *p*/kPa=75.154 *T*/K = 273.1615	
*f*/Hz	*g*/Hz
2475.9438	0.5300
4256.9563	0.7094
6008.8062	0.8554
7751.4103	0.9980
9489.8906	1.1392
11226.0768	1.2998
11226.0768	1.3003
9489.8899	1.1367
7751.4173	0.9980
6008.8090	0.8569
4256.9573	0.7059
2475.9475	0.5265

**Table:**

Apr 5, 1986 *p*/kPa=25.396 *T*/K = 273.1640	
*f*/Hz	*g*/Hz
2475.4560	0.8932
4256.2613	1.2318
6007.9202	1.5216
7750.3487	1.7909
9488.6706	2.0601
11224.7581	2.5322
11224.7521	2.5631
9488.6751	2.0703
7750.3874	1.8123
6007.9058	1.5123
4256.2608	1.2243
2475.4644	0.9006

## Appendix 2. Speed of Sound Squared in Ar-M at *T*_t_

The speed of sound squared in Ar-M at *T*_t_ is tabulated as a function of the pressure and the mode used for the measurement. The square of the speed of sound was determined from the frequencies in Appendix 1 and includes corrections for the thermal boundary layer, the elastic response of the shell, the unequal radii of the hemispheres (in the second order of shape perturbation theory), and the difference between the temperature of the measurement and *T*_t_. For the sake of definiteness, the correction for the thermal boundary layer uses the value 1 for the thermal accommodation coefficient.

**Table:**

*p*/kPa=504.177		*p*/kPa=401.361		*p*/kPa=299.937	
0,2	94883.396	0,2	94855.267	0,2	94828.439
0,3	94883.346	0,3	94855.194	0,3	94828.454
0,4	94883.321	0,4	94855.196	0,4	94828.471
0,5	94833.320	0,5	94855.214	0,5	94828.487
0,6	94883.270	0,6	94855.205	0,6	94828.516
		
*p*/kPa=199.894		*p*/kPa=100.261		*p*/kPa=449.295	
0,2	94803.326	0,2	94779.334	0,2	94868.238
0,3	94803.321	0,3	94779.347	0,3	94868.142
0,4	94803.308	0,4	94779.360	0,4	94868.116
0,5	94803.320	0,5	94779.230	0,5	94868.163
0,6	95803.362	0,6	94779.294	0,6	94868.147
		
*p*/kPa=350.096		*p*/kPa=250.250		*p*/kPa=149.792	
0,2	94841.578	0,2	94815.834	0,2	94791.066
0,3	94841.552	0,3	94815.768	0,3	94791.065
0,4	94841.563	0,4	94815.831	0,4	94791.076
0,5	94841.577	0,5	94815.814	0,5	94791.088
0,6	94841.587	0,6	94815.865	0,6	94791.125
		
*p*/kPa=50.041		*p*/kPa= 101.371		*p*/kPa=75.154	
0,2	94767.808	0,2	94779.808	0,2	94773.462
0,3	94767.572	0,3	94779.590	0,3	94773.355
0,4	94767.564	0,4	94779.648	0,4	94773.430
0,5	94767.723	0,5	94779.603	0,5	94773.390
0,6	94767.528	0,6	94779.585	0,6	94773.397
		
*p*/kPa = 50.017		*p*/kPa = 25.396			
0,2	94767.836	0,2	94763.099		
0,3	94767.576	0,3	94762.457		
0,4	94767.494	0,4	94761.858		
0,5	94767.586	0,5	94762.258		
0,6	94767.750	0,6	94761.909		
		

## Appendix 3. Resonance Frequencies and Half-Widths for Measurement of Speed of Sound Ratios near *T*_t_

The frequencies and half-widths in these tables result from fitting six parameters in eq (4.1) to the measured voltage as a function of frequency. In each table, the frequencies appear in the order in which they were measured. The last two entries for Ar-M on May 14, 1986 were influenced by intermittent arcing in a transducer. These entries were not used in the computation of the speed-of-sound ratios.

**Table:**

Isotopically enriched Ar-40					
May 14, 1986 *p*/kPa=130.565 *T*/K=273.1910		May 20, 1986 *p*/kPa=116.567 *T*/K=273.2014		May 21, 1986 *p*/kPa=103.570 *T*/K=273.2124	

*f*/Hz	*g*/Hz	*f*/Hz	*g*/Hz	*f*/Hz	*g*/Hz
2475.9112	0.4009	2475.8900	0.4239	2475.8761	0.4490
4256.8550	0.5322	4256.8292	0.5630	4256.8164	0.5977
6008.6264	0.6482	6008.5974	0.6853	6008.5866	0.7288
7751.1476	0.7481	7751.1160	0.7929	7751.1071	0.8419
9489.5342	0.8410	9489.5017	0.8921	9489.4967	0.9497
9489.5330	0.8414	9489.5032	0.8926	9489.4965	0.9494
7751.1458	0.7481	7751.1171	0.7924	7751.1062	.0.8419
6008.6246	0.6483	6008.5990	0.6858	6008.5858	0.7283
4256.8535	0.5320	4256.8304	0.5629	4256.8156	0.5980
2475.9097	0.4011	2475.8917	0.4230	2475.8754	0.4487

**Table:**

Working gas: Ar-M					
May 14, 1986 *p*/kPa=130.564 *T*/K=273.1883		May 20, 1986 *p*/kPa=116.838 *T*/K=273.2074		May 22, 1986 *p*/kPa=103.468 *T*/K=273.2158	

*f*/Hz	*g*/Hz	*f*/Hz	*g*/Hz	*f*/Hz	*g*/Hz
2476.3542	0.4011	2476.3759	0.4223	2476.3470	0.4502
4257.6170	0.5327	4257.6638	0.5625	4257.6243	0.5988
6009.7018	0.6481	6009.7753	0.6848	6009.7282	0.7292
7752.5341	0.7480	7752.6350	0.7916	7752.5792	0.8422
9491.2318	0.8420	9491.3607	0.8915	9491.2997	0.9506
9491.2305	0.8419	9491.3612	0.8913	9491.2998	0.9497
7752.5330	0.7486	7752.6347	0.7920	7752.5792	0.8428
6009.7008	0.6480	6009.7750	0.6854	6009.7275	0.7291
4257.6151	0.5321	4257.6641	0.5623	4257.6243	0.5990
2476.3522	0.4009	2476.3751	0.4225	2476.3469	0.4503
